# PBPK Simulation-Based Evaluation of Ganciclovir Crystalluria Risk Factors: Effect of Renal Impairment, Old Age, and Low Fluid Intake

**DOI:** 10.1208/s12248-021-00654-1

**Published:** 2021-12-14

**Authors:** Daniel Scotcher, Aleksandra Galetin

**Affiliations:** Centre for Applied Pharmacokinetic Research, School of Health Sciences, University of Manchester, Stopford Building, Oxford Road, Manchester, M13 9PT UK

**Keywords:** Chronic kidney disease, Physiologically based pharmacokinetic model, Kidney model

## Abstract

Dosing guidance is often lacking for chronic kidney disease (CKD) due to exclusion of such patients from pivotal clinical trials. Physiologically based pharmacokinetic (PBPK) modelling supports model-informed dosing when clinical data are lacking, but application of these approaches to patients with impaired renal function is not yet at full maturity. In the current study, a ganciclovir PBPK model was developed for patients with normal renal function and extended to CKD population. CKD-related changes in tubular secretion were explored in the mechanistic kidney model and implemented either as proportional or non-proportional decline relative to GFR. Crystalluria risk was evaluated in different clinical settings (old age, severe CKD and low fluid intake) by simulating ganciclovir medullary collecting duct (MCD) concentrations. The ganciclovir PBPK model captured observed changes in systemic pharmacokinetic endpoints in mild-to-severe CKD; these trends were evident irrespective of assumed pathophysiological mechanism of altered active tubular secretion in the model. Minimal difference in simulated ganciclovir MCD concentrations was noted between young adult and geriatric populations with normal renal function and urine flow (1 mL/min), with lower concentrations predicted for severe CKD patients. High crystalluria risk was identified at reduced urine flow (0.1 mL/min) as simulated ganciclovir MCD concentrations exceeded its solubility (2.6–6 mg/mL), irrespective of underlying renal function. The analysis highlighted the importance of appropriate distribution of virtual subjects’ systems data in CKD populations. The ganciclovir PBPK model illustrates the ability of this translational tool to explore individual and combined effects of age, urine flow, and renal impairment on local drug renal exposure.

**Graphical Abstract:**

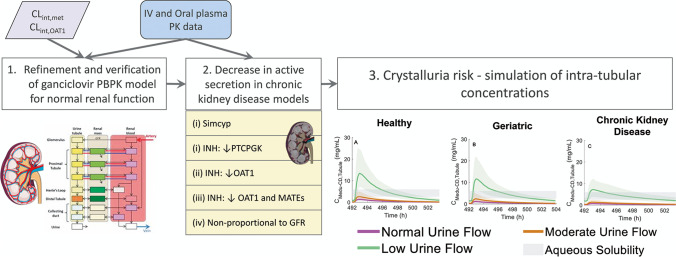

**Supplementary Information:**

The online version contains supplementary material available at 10.1208/s12248-021-00654-1.

## INTRODUCTION

The global prevalence of chronic kidney disease (CKD) and associated burden on healthcare is substantial and rising, concurrent with an increase in aging population [1]. Due to association of impaired renal function with co-morbidities, particularly cardiovascular disease and diabetes, CKD patients frequently require polypharmacy to support their health needs. As a result, the likelihood of drug-drug interactions is increased, which, in addition to impaired renal and hepatic drug elimination, increases the risk of adverse drug events for these patients, including drug-induced kidney injury [2, 3]. The above factors represent a challenge for drug developers, regulators, and prescribers to manage safely the drug dosing needs of CKD patients. Exacerbating the situation, CKD dosing guidance is often lacking from drug product labels because these patients are generally excluded from pivotal clinical trials, although recent efforts from clinicians and regulatory bodies aim to make trials more inclusive [4–8].

Physiologically based pharmacokinetic (PBPK) modelling and simulation is a useful translational tool to support drug development and clinical practice [9–13]. PBPK modelling allows exploration and identification of physiological and demographic characteristics, in addition to drug properties, that are the most important in determining drug systemic and tissue exposure [14–16]. By accounting for the combinatorial effects of multiple risk factors, PBPK simulations enable identification of patients at higher risk of adverse events. A recently published draft of the US Food and Drug Administration (FDA) Guidance suggested that PBPK simulations may inform early characterisation of pharmacokinetics (PK) in renally impaired subjects, to support the inclusion of these patients in late-phase clinical trials, with appropriate adjustment to dose, if required [5]. In addition, PBPK modelling may be useful to inform dosage adjustment in chronic kidney disease patients when labelling information is lacking.

**Fig. 1:**
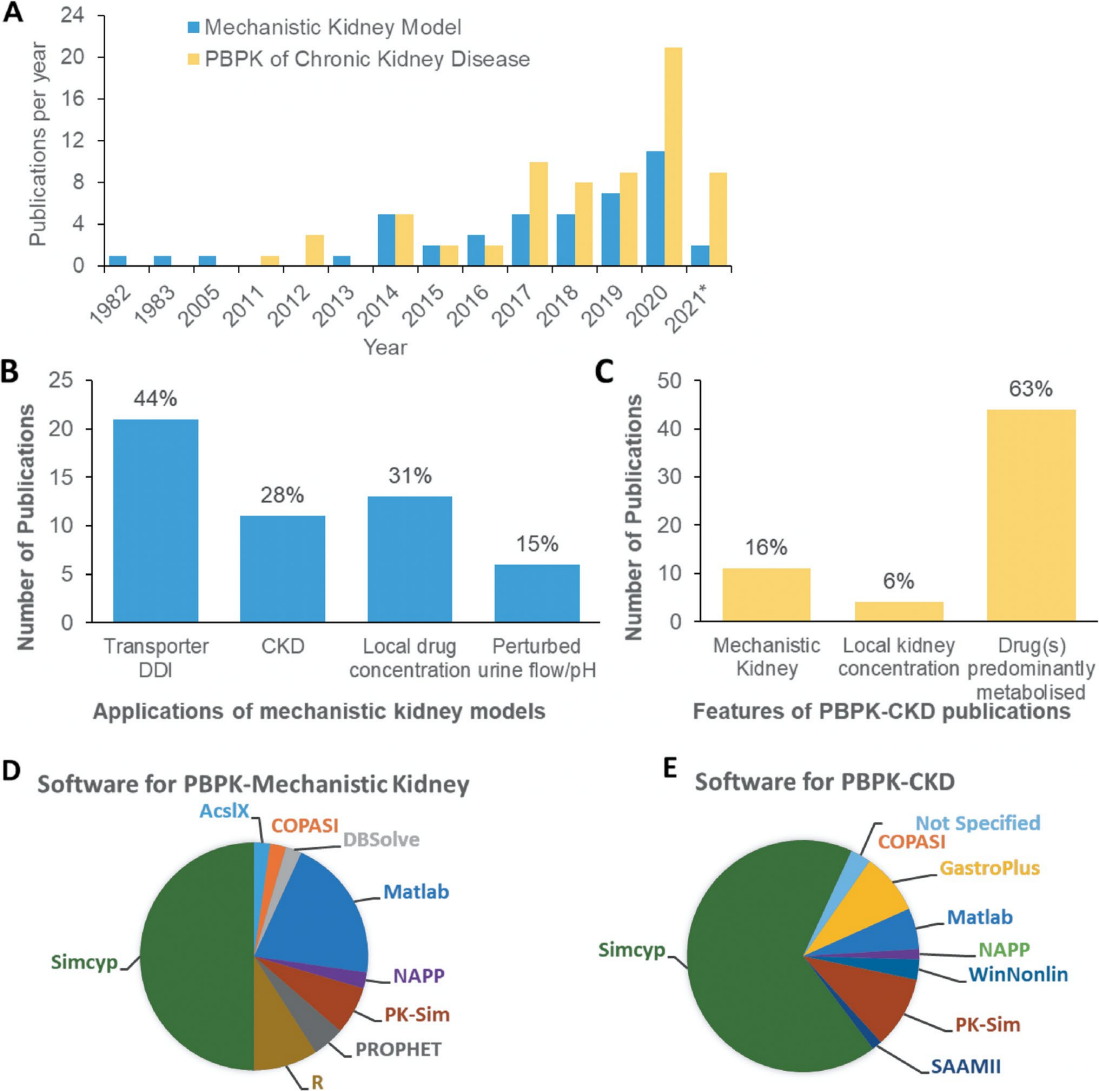
Analysis of publication trends in PBPK modelling applications with respect to mechanistic kidney models and chronic kidney disease following an extensive search of the literature. **A** Publications per year reporting PBPK models and their use, with clinical (human) applications (either theoretical or real-world situations). **B** Frequencies (non-exclusive) of selected applications for PBPK-mechanistic kidney models in literature. **C** Frequencies (non-exclusive) of selected features of published reports of PBPK modelling and simulation in chronic kidney disease. **D** and **E** Relative use of software in publications reporting PBPK-mechanistic kidney models (Panel D) or PBPK simulation in chronic kidney disease (**E**). Inclusion/ exclusion criteria of the literature search, references, and additional analyses are presented in Supplemental material, Sect. 1 (Tables S1–S3). *2021 is an incomplete year (→ April 2021)

Extensive critical analysis of the peer-reviewed literature data (details in Supplemental Material, Sect. 1) revealed a surge in publications reporting PBPK simulations in CKD over the last decade (Fig. 1A). Such models separate the contributions of glomerulus and various regions of nephron (e.g. proximal tubule) to local (e.g. intracellular, nephron luminal filtrate, urine) and systemic (e.g. plasma) concentrations of drugs and endogenous metabolites. These PBPK models have been applied to a variety of scenarios ranging from prediction of transporter-mediated drug-drug interactions to simulation of effect of perturbed urine flow or pH (Fig. 1B) on drug systemic exposure and elimination. Surprisingly, > 60% of reported PBPK-CKD modelling efforts focused on drugs that are eliminated predominantly by hepatic metabolism (Fig. 1C). In contrast, relatively lower attention has been given to simulating the effects of CKD on renal excretion using mechanistic kidney models (16%) and on local drug exposure in kidney (6%) (Fig. 1C), highlighting gaps in the current research. Although commercial PBPK software platforms are widely applied in the literature (Fig. 1E), a thorough evaluation of their capability to generate realistic virtual trial subjects for different stages of CKD is still lacking.

It is commonly acknowledged that both renal tubular secretion processes and glomerular filtration are affected by CKD [17]. However, the exact mechanism(s) responsible for the decline in tubular secretion function, and the relative extent of decline compared to glomerular filtration, are not fully characterised [14, 18]. Proposed mechanisms include (i) loss of functional proximal tubular cells; (ii) loss of transporter expression in each functional proximal tubular cell; (iii) renal transporter inhibition by uremic toxins; and (iv) perturbations to the (proposed) albumin-facilitated transport mechanism [14, 19, 20]. These proposed disease-related changes in active secretion may be either proportional (i.e. in line with the ‘intact nephron hypothesis’ (INH, [21]) or non-proportional to corresponding changes in glomerular filtration rate. The consistency of these various plausible mechanisms (and their extent) in explaining clinical PK data in CKD patients has been explored using both empirical and mechanistic/PBPK models [14, 20, 22–26]. In addition to plasma concentrations, the structural complexity of mechanistic kidney models enables simulation of local (intra-tubular) drug exposure and regional tubular differences and assessment of certain scenarios (e.g. risk of crystalluria in low urine flow conditions) [27–29]. However, such simulations have not yet been reported for CKD patients.

Treatment with several antivirals is associated with crystal precipitation of the drug in the nephron tubules, in some cases leading to nephrolithiasis (kidney stone formation), tubular obstruction, and subsequently acute kidney injury [30]. Precipitation occurs because of the urinary concentrating mechanism in the nephron tubule, resulting in high drug concentrations within the tubular filtrate that exceed the aqueous solubility. Patients prescribed these drugs are advised to maintain fluid intake to reduce risk of intra-tubular precipitation [31, 32]. Limited evidence suggests that pre-existing CKD could be a risk factor for this specific mechanism of kidney damage [33]. Clinical information on whether older age is intrinsically a risk factor for drug-induced nephrolithiasis is not clear, although older age is generally associated with reduced fluid intake.

The aim of the current study was to perform PBPK simulations of the local renal disposition of ganciclovir in patients with different severity of renal impairment and explore the relationship between ganciclovir plasma and renal exposure and various demographic and physiological properties. Ganciclovir was selected as a model drug as it is predominantly eliminated unchanged in urine, in part through OAT1 and OAT2-mediated renal active secretion [34–37], is hydrophilic with poor membrane permeability [38] (i.e. negligible tubular reabsorption expected), and has clinical reports of crystal precipitation in urine. Impaired renal function has been associated with significantly reduced ganciclovir renal excretion clearance (CL_R_), reflected in recommended dose adjustment in the drug product label for these patients [31]. In the current study, crystalluria risk was evaluated by PBPK modelling of different clinical settings (old age, severe CKD, and low fluid intake, in isolation and as combined risk factors) and by simulating medullary collecting duct concentrations of ganciclovir. In addition, robustness of CKD population models and distribution of physiological/system parameters in virtual patients were critically discussed.

## METHODS

Ganciclovir clinical pharmacokinetic data in healthy and subjects with varying severities of chronic kidney disease, together with corresponding demographics and trial design data, were collated from the peer-reviewed literature and FDA Clinical Pharmacology Reviews and Approved Drug Labels. In total, PK data for normal renal function were available for 64 subjects (5 clinical studies) after intravenous administration of ganciclovir and 82 subjects (5 studies) after oral administration of its prodrug valganciclovir (Supplemental Material, Tables S4–S6). Data from liver transplant recipients [39] were excluded from the current analysis due to (i) reported limitation of using serum creatinine as a renal function biomarker in these patients and (ii) potential pharmacokinetic differences compared with healthy subjects [40]. For CKD patients, clinical PK data were available for 8 subjects after IV administration of ganciclovir and 18 subjects following oral administration of valganciclovir (Supplemental Material, Tables S7–S10). Subjects with different severity of renal impairment were included, with a tenfold range between highest and lowest creatinine clearance and > 40-fold range in serum creatinine.

**Fig. 2:**
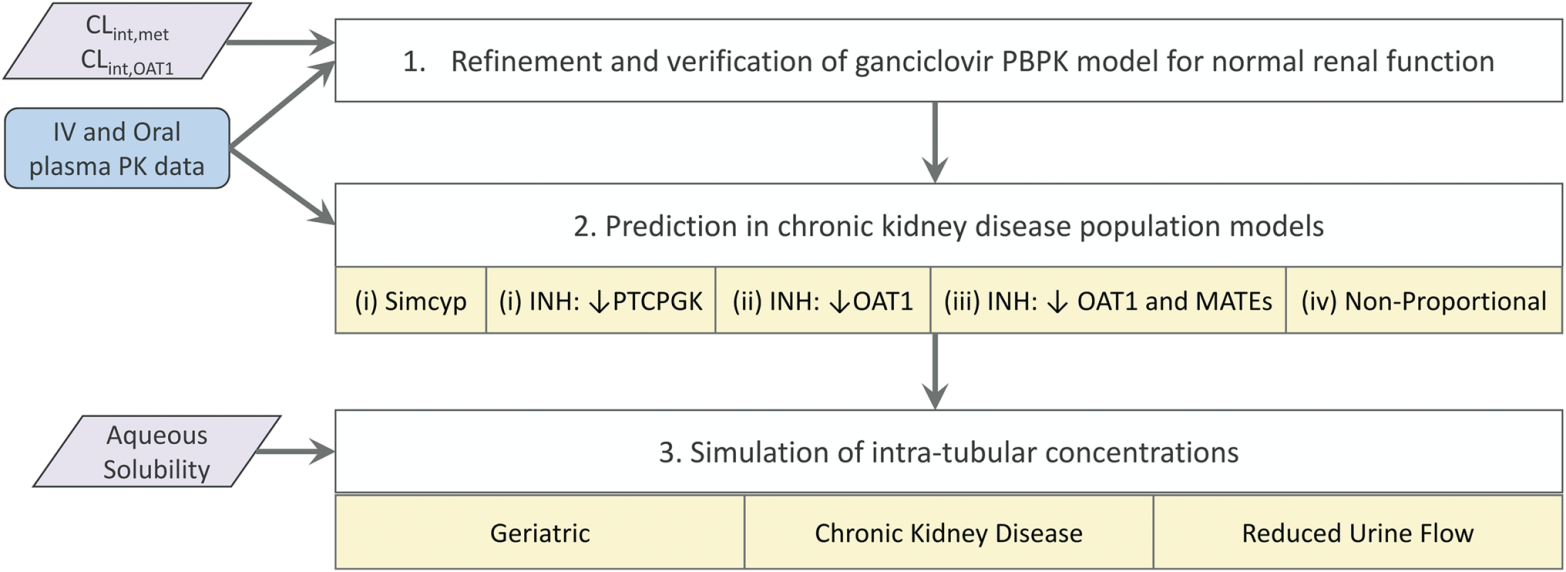
Workflow scheme for physiologically-based pharmacokinetic (PBPK) modelling and simulation of ganciclovir (GCV). [1] Previously published ganciclovir PBPK model for the Simcyp simulator platform [23] was refined for use in v19.1 of the platform and based on an updated literature evaluation of ganciclovir pharmacokinetics. [2] The ganciclovir PBPK model was verified by comparison of simulated and observed pharmacokinetic data for ganciclovir after intravenous (IV) administration of ganciclovir, or oral administration of the pro-drug valganciclovir (VGCV) in subjects with normal renal function. [3] Ganciclovir pharmacokinetics in chronic kidney disease patients were predicted with various chronic kidney disease population models; these models were (i) Simcyp renal impairment populations [48] that assume no impact of kidney disease on proximal tubule cellularity (PTCPGK) or transporter abundance, and modified Simcyp renal impairment populations assuming either (ii) PTCPGK is reduced in CKD proportionally to the decrease in glomerular filtration rate vs normal renal function, consistent with the intact nephron hypothesis (INH); (iii) renal organic anion transporter (OAT)1 abundance is reduced in CKD proportionally to the decrease in glomerular filtration rate vs normal renal function, consistent with the intact nephron hypothesis (INH); (iv) both OAT1 and renal multidrug and toxin extrusion (MATE) abundance are reduced in CKD proportionally to the decrease in glomerular filtration rate vs normal renal function, consistent with the INH, and (v) PTCPGK is reduced in CKD proportionally to the decrease in glomerular filtration rate vs normal renal function, consistent with the (INH), while OAT1 activity is reduced to mimic the impact of transporter inhibition by uremic solutes. [4] Ganciclovir concentrations in the medullary collecting duct are simulated in virtual populations with varying urine flow rate (0.1–1 mL/min) and compared with aqueous solubility data for ganciclovir (2600–6000 mg/L)

PBPK modeling and simulation were performed in a step-wise manner (Fig. 2) using the Simcyp simulator platform (version 19.1; Certara, Sheffield, UK). Whenever possible, simulations were performed following the specific trial designs reported in the clinical studies and with 100 virtual trials. Simulations of hypothetical scenarios (i.e. for which specific clinical data were not available for comparison) used at least 200 virtual subjects per virtual population to simulate inter-individual variability.

### Refinement of Ganciclovir PBPK Model: Subjects with Normal Renal Function

Drug-specific model parameters were previously reported for ganciclovir using an earlier version (v15r1) of the software [23]. The corresponding Simcyp compound file was obtained from the Simcyp online repository (https://members.simcyp.com/account/repository/). The ganciclovir model used first-order absorption model (oral administration) or zero-order injection/infusion (intravenous administration), whole-body distribution model with all tissues described as perfusion rate limited, with the exception of kidney [41, 42]. Ganciclovir elimination was predominantly by renal excretion, with a minor contribution from metabolism which was arbitrarily assigned to liver. The permeability-limited kidney model used here was reported previously [43, 44]; model scheme is shown in Supplementary Figure S1.

Verification of the drug-specific parameters was done to ensure the model performance in the current software version. Due to inconsistencies between simulated and observed PK data after intravenous administration of ganciclovir in subjects with normal renal function, the ganciclovir PBPK model was refined by reverse translation from the IV clinical data (e.g. using parameter estimation/ model fitting or sensitivity analysis) [9, 45] (Table S11). In particular, the assumed fraction of ganciclovir excreted in urine (fe) and CL_R_ was refined, through identification of the hepatic metabolic intrinsic clearance (CL_int,met_; not attributed to any specific enzyme) and organic anion transporter (OAT)1 intrinsic clearance (CL_int,OAT1_) parameters. Although in vitro evidence suggests that ganciclovir may also be substrate for OAT2 and OAT3 [37], delineating contributions of individual OAT transporters to ganciclovir uptake into proximal tubule cells were outside of scope of current study. Thus, transporter-mediated uptake into proximal tubule cells was considered as a single ‘lumped’ uptake transporter parameter in the model, termed herein as ‘OAT1’, consistent with the previous publication [23]. Oral absorption and hydrolytic conversion of the prodrug valganciclovir to ganciclovir were described by a first-order absorption rate constant (*k*_*a*_), as previously reported [23]. The pharmacokinetic data following oral administration of valganciclovir were used for validation of ganciclovir PBPK model. Sensitivity and uncertainty of drug-specific parameters were assessed to evaluate model reliability (details in Supplementary material, Sect. 6).

### Simulations of Ganciclovir Pharmacokinetics in Renal Impairment Populations

The pharmacokinetics of ganciclovir in subjects with impaired renal function was simulated using a series of virtual population models of renal impairment (Table I), adapted from previous reports [14, 19, 23, 46, 47]. Initial simulations used existing “Sim-RenalGFR_30-60” and “Sim-RenalGFR_less_30” virtual populations in the Simcyp simulator (v19r1) (Table S13 and Box S1). These virtual patient populations account for disease-related differences in demographic (e.g. age) and system parameters (e.g. GFR, kidney size, plasma protein concentrations) [48], with urine flow initially assumed to be unaffected by renal impairment. Subsequently, additional, biologically plausible, CKD virtual population models were investigated (Table I). Three models based on mimicking the INH [21, 22] assumed a decrease of either (i) proximal tubule cellularity, (ii) OAT1 abundance in proximal tubule cells, or (iii) OAT1 and multidrug and toxin extrusion (MATE) abundance in proximal tubule cells, in proportion to the disease-related decrease in GFR. Another assumption explored was that proximal tubule cellularity declines proportionally to GFR, while changes in OAT1 activity were disproportionate to these changes which would account for the effect of uremic solutes on OAT1 (Table I). Variances in proximal tubule cellularity and transporter abundances in CKD virtual population models were the same as for healthy, due to paucity of data.

**Table I:** Summary of modifications in system/physiological parameters in renal impairment virtual population models used for simulations of ganciclovir systemic and regional nephron concentrations

Population name	Description of assumptions	Mean (%CV) parameter values for moderate/severe renal impairment virtual populations		
		PTCPGK (10^6^ cells/ g kidney)	OAT1 abundance per 10^6^ PTC in the kidney (relative to healthy)	MATEs abundance per 10^6^ PTC in the kidney (relative to healthy) ^a^
Simcyp default CKD model	Changes to age distributions, serum creatinine/GFR, kidney weight, haematocrit, plasma protein concentrations [48] No change in PTCPGK or transporter abundance vs healthy	60 (30%)/60 (30%)	1 (60%)/1 (60%)	1 (60%)/1 (60%)
INH: PTCPGK ^b^	As per default CKD model, but also assuming that proximal tubule cellularity per gram kidney decreases proportionally to GFR	21 (30%)/10.2 (30%)	1 (60%)/1 (60%)	1 (60%)/1 (60%)
INH: OAT1	As per default CKD model, but also assuming that OAT1 protein abundance decreases proportionally to GFR	60 (30%)/60 (30%)	0.35 (60%)/0.17 (60%)	1 (60%)/1 (60%)
INH: OAT1 and MATEs	As per default CKD model, but also assuming that abundances of OAT1 and MATEs reduce proportionally to GFR	60 (30%)/60 (30%)	0.35 (60%)/0.17 (60%)	0.35 (60%)/0.17 (60%)
INH: PTCPGK ^b^ + Uremic Solutes	As per “INH: PTCPGK” population, but with additional consideration of inhibitory effects of uremic solutes on OAT1 ^c^ [19, 23]	21 (30%)/10.2 (30%)	0.73 (60%)/0.41(60%)	1 (60%)/1 (60%)

*GFR* glomerular filtration rate; *INH* intact nephron hypothesis; *PTC* proximal tubule cells; *PTCPGK* proximal tubule cellularity per gram kidney; *OAT1* organic anion transporter 1; *MATE* multidrug and toxin extrusion

^a^ Information on MATEs is included herein for completeness and INH-based changes were considered as a default. MATE CL_int_ was an insensitive parameter with respect to simulated plasma and intra-tubular concentrations (see Sect. 6 in Supplementary Material); therefore, consideration of changes in MATE beyond INH as previously reported [26] were not warranted for ganciclovir

^b^Total kidney CL_int,T,i_ is the same for “INH:PTCPGK” and “INH: OAT1 and MATEs” populations (for details see Table S11)

^c^Based on analysis of clinical data for 18 substrates for OAT1/3. Comparable, but slightly less pronounced changes (0.68 in moderate CKD, 0.59 in severe CKD) were reported for OAT2 based on the analysis of penciclovir data [26]

The simulated distributions of key demographic parameters for these CKD virtual populations were compared with those of the “Healthy Volunteer” virtual population (“Sim-Healthy Volunteers”), following a simulation 1000 virtual subjects for each population. The agreement of simulated plasma concentration–time data or PK parameters with clinical observations was evaluated for each CKD virtual population model. Those renal impairment population models that were inconsistent with the observed systemic pharmacokinetic data were excluded from subsequent analysis. Ganciclovir PBPK model performance was evaluated by comparing simulated and observed ganciclovir PK data vs. reported renal function endpoints on continuous scales and visual predictive checks.

### Effect of Age, Chronic Kidney Disease, and Urine Flow Rate on Simulated Ganciclovir Concentration in Nephron Tubule

Ganciclovir luminal concentrations in each region of the renal nephron (proximal tubule, loop of Henle, distal tubule and collecting duct) were simulated for different age groups (18–65, 18–95, and 65–95 years) and under various conditions of perturbed renal physiology, namely CKD, and reduced urine flow rate. The “Geriatric” (65–95 years) virtual population accounted for age-related changes in physiology such as reduced cardiac output and decline in renal function, while assuming no age-dependent change in proximal tubule cellularity or relative transporter abundances, and initially assuming no change in urine flow rate (1 mL/ min) compared with healthy volunteers. Simulations were performed with 900 mg valgancyclovir, administered either as single dose or twice daily for 21 days as per dosage guidelines for subjects with normal renal function [31]. Urine flow values were varied within physiologically realistic range (0.1–1 mL/min), as investigated previously for drugs with differing permeability properties [28], and recognising that urine production capacity is retained at almost normal levels in CKD patients until renal failure is reached [49]. Filtrate flow rates for various scenarios are listed in Table S14; comparison with those recently reported for a custom CKD model [49] is in Figure S5. Where appropriate, simulated concentrations in different regions of the nephron were compared with aqueous solubility values for ganciclovir (2600–6000 mg/L; [50]) to assess the possibility of precipitation and crystalluria risk. An important technical consideration when changing the urine flow rate in the Simcyp v19.1 from the default set values is that the same filtrate flow rates, including glomerular filtration rate, had to be applied to all virtual subjects simulated from that population file. As such, no inter-individual variability in glomerular filtration rate or any other tubular flow rate could have been simulated in this manner. In addition, GFR cannot be reduced below 50 mL/min/1.73 m^2^ (approx. 40% of healthy) if urine flow rate is also altered.

### Distributions of Simulated Virtual Subjects

The uni- and bi-variate distributions of demographic and systems data (e.g. age, body weight, kidney weight, GFR) were assessed for 1000 virtual subjects simulated from each of the “Healthy Volunteer”, “Sim-RenalGFR_30-60”, and “Sim-RenalGFR_less_30” population models provided with the Simcyp simulator. Age ranges and proportion of male/female were consistent with the defined populations. Further information is provided in Supplemental material, Sect. 8.

### Assessment of Model Performance

Predictive/descriptive performance of specific PBPK model simulations was assessed by comparing the observed data with the median, 5th and 95th percentiles of simulated trials. Overall performance was evaluated using three commonly used metrics, namely the average fold error (AFE; Eq. 1), geometric mean fold error (GMFE; also termed absolute average fold error; Eq. 2), and root mean square error (RMSE; Eq. 3).

1 $$AFE= {10}^{\frac{1}{n}\sum \mathrm{log}\left(\frac{{Predicted}_{i}}{{Observed}_{i}}\right)}$$

2 $$GMFE= {10}^{\frac{1}{n}\sum \left|\mathrm{log}\left(\frac{{Predicted}_{i}}{{Observed}_{i}}\right)\right|}$$

3 $$RMSE=\sqrt{\frac{\sum {\left({Predicted}_{i}- {Observed}_{i}\right)}^{2}}{n}}$$

For model validation, acceptance criteria were (i) individual studies within 99% confidence interval around the geometric mean for observed data [51] and (ii) GMFE and AFE within 1.25-fold (bioequivalence limits) and twofold (commonly used criteria for pharmacokinetic predictions [52]).

## RESULTS

### Refinement and Validation of Ganciclovir PBPK Model in Normal Renal Function

**Fig. 3:**
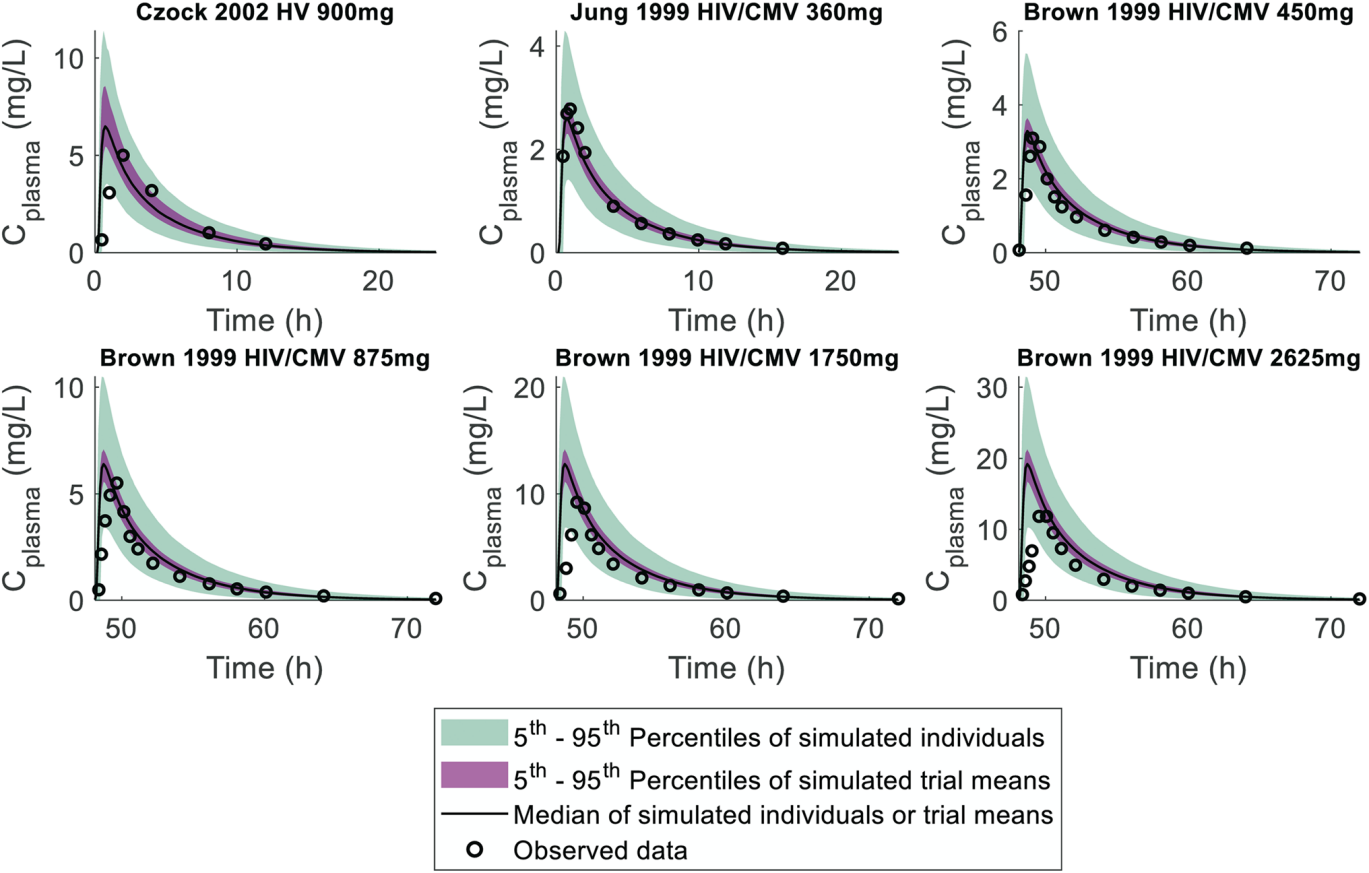
Predicted plasma concentration–time profiles for ganciclovir, after oral administration of valganciclovir, using refined PBPK model elimination parameters with comparison with observed data, for model verification. Median and 5th–95th percentile ranges of the simulated trial means, and of the individual profiles, were calculated from 100 virtual trials per clinical study. Study designs are listed in Table S4. C_plasma_ concentration of ganciclovir in plasma; HIV/CMV subjects seropositive for human immunodeficiency virus or cytomegalovirus; HV healthy volunteers

Initial PBPK simulations were performed for subjects with normal renal function using a previously reported model for ganciclovir [23]. Although the simulated plasma concentration–time profiles after intravenous administration of ganciclovir captured reasonably well the observed data (Figure S8 and S9), CL_R_ of ganciclovir was systematically over-predicted (AFE = 1.31) (Figure S10, Table S15). Therefore, the fraction of ganciclovir excreted in urine was refined from 1 [23] to 0.88 [53, 54]. Process of parameterisation of the ganciclovir PBPK model is detailed in Supplementary Material, Sect. 10. Following refinement of the ganciclovir model, the simulated pharmacokinetic profiles after IV administration were in good agreement with the observed data in subjects with normal renal function, with GMFE and AFE < 1.25 fold-error for all pharmacokinetic parameters (Figure S11-S13, Table S17). Validation of this ganciclovir PBPK model was performed using pharmacokinetic data following oral (valganciclovir) administration (Fig. 3, Figure S14). Predicted maximum plasma concentration (*C*_max_), AUC_0-inf_, AUC_0-t_, CL/F, V_d_/F, and CL_R_ were within 99% confidence interval and around the geometric mean for observed data for all studies (Table S18); AFE and GMFE were within twofold error for these parameters, as well as *t*_1/2_ (Table S18). *T*_max_ data were typically under-predicted (AFE = 0.47), while *k*_*a*_ was identified as a parameter with moderate uncertainty and high sensitivity toward *C*_max_ in medullary collecting duct (Figure S4).

### Predictions of Ganciclovir Pharmacokinetics in Chronic Kidney Disease

**Fig. 4:**
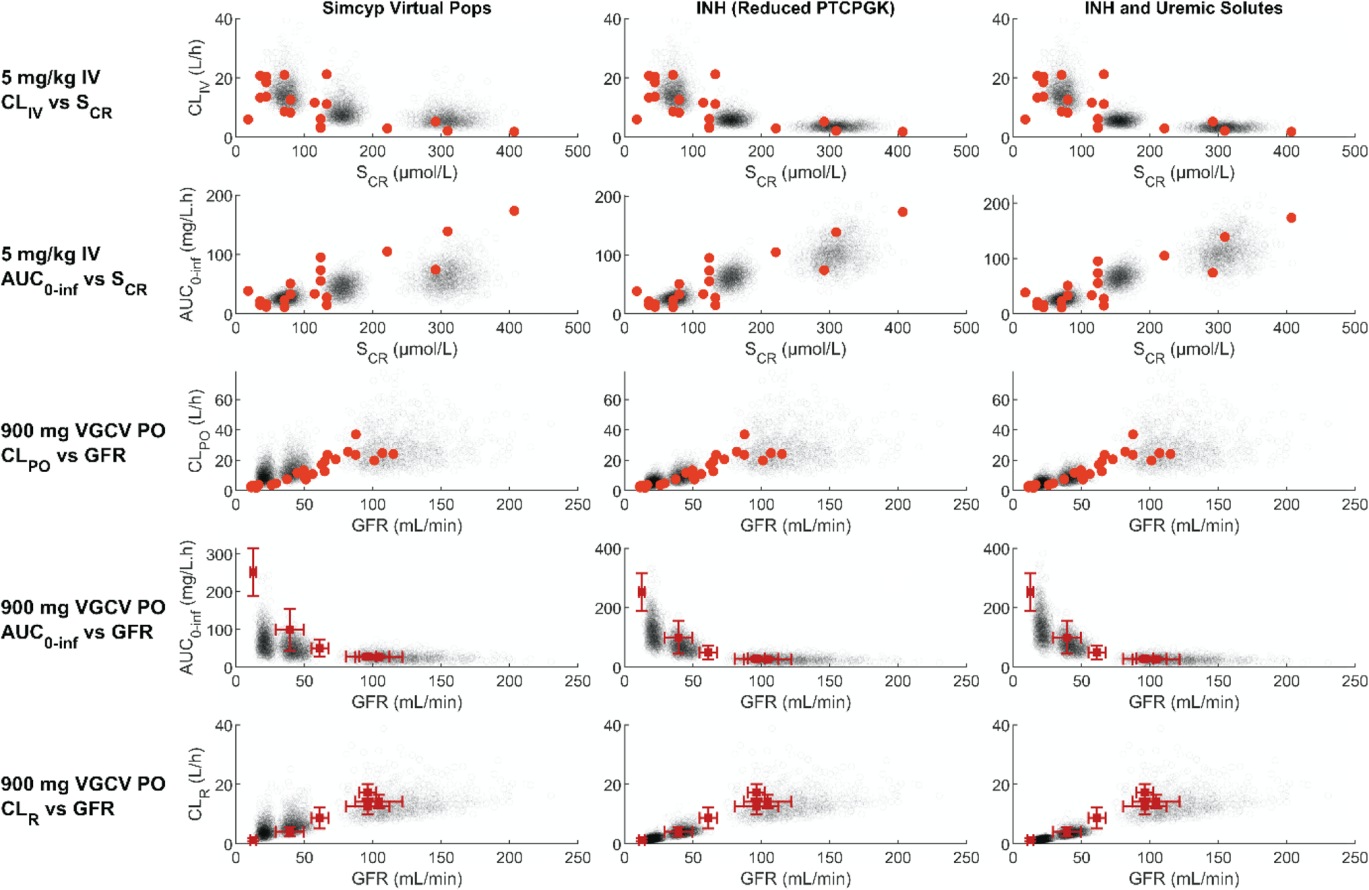
Simulated and observed pharmacokinetic parameters and glomerular filtration rate (GFR) or serum creatinine concentration (S_CR_), in subjects with normal and impaired renal function. Comparisons were made on basis of reported clinical endpoints in respective studies. The “Simcyp Virtual Pops” renal impairment population models did not consider disease related changes in proximal tubule cells per gram kidney (PTCPGK) or organic anion transporter (OAT)1 activity; the “INH (Reduced PTCPGK)” population models accounted for disease related change in PTCPGK in accordance with the intact nephron hypothesis (INH); the “INH and Uremic Solutes” population models accounted for both INH-based reduction in PTCPGK and reduced OAT1 activity in accordance with reported inhibitory effects of uremic solutes on OAT1 in renal impairment (Table I). Simulations (grey open circles) were performed with 1000 virtual subjects from each of the normal renal function (GFR > 90 mL/min), moderate renal impairment (30 mL/min < GFR < 60 mL/min) and severe renal impairment (GFR < 30 mL/min) populations, following the overall design of specific clinical studies (Table S7). Observed clinical data presented are for individuals (bright red filled circles) or mean ± standard deviation of multiple individuals (dark red filled squares and error bars). AUC_0-inf_ area under the ganciclovir plasma concentration–time curve extrapolated to infinity; CL/F oral plasma clearance; CL_IV_ Clearance after intravenous administration; CL_R_ renal excretion clearance; GFR glomerular filtration rate; INH Intact nephron hypothesis; IV intravenous administration; MATE Multidrug and toxin extrusion; OAT1 Organic anion transporter 1; PO oral administration; PTCPGK Proximal tubule cellularity per gram kidney; VGCV valganciclovir

Following successful optimisation of ganciclovir model in healthy, multiple CKD virtual population scenarios were explored, as detailed in the PBPK workflow (Fig. 2 and Table I). Exact representation of the clinical trials’ design in the CKD virtual populations was not possible, as the GFR for the most severe CKD patients in the clinical trials were above the allowed cap (15 mL/min/1.73 m^2^) in Simcyp version 19.1. As such, direct comparison of predicted and observed PK endpoints (e.g. AUC, CL_R_) and profiles for specific clinical trials in patients was not feasible (as performed for healthy, see above, and previous simulations in CKD [23]). Therefore, predictive performance was assessed using visual predictive checks with simulated data of 1000 virtual subjects for each discrete virtual population (Fig. 4).

**Fig. 5:**
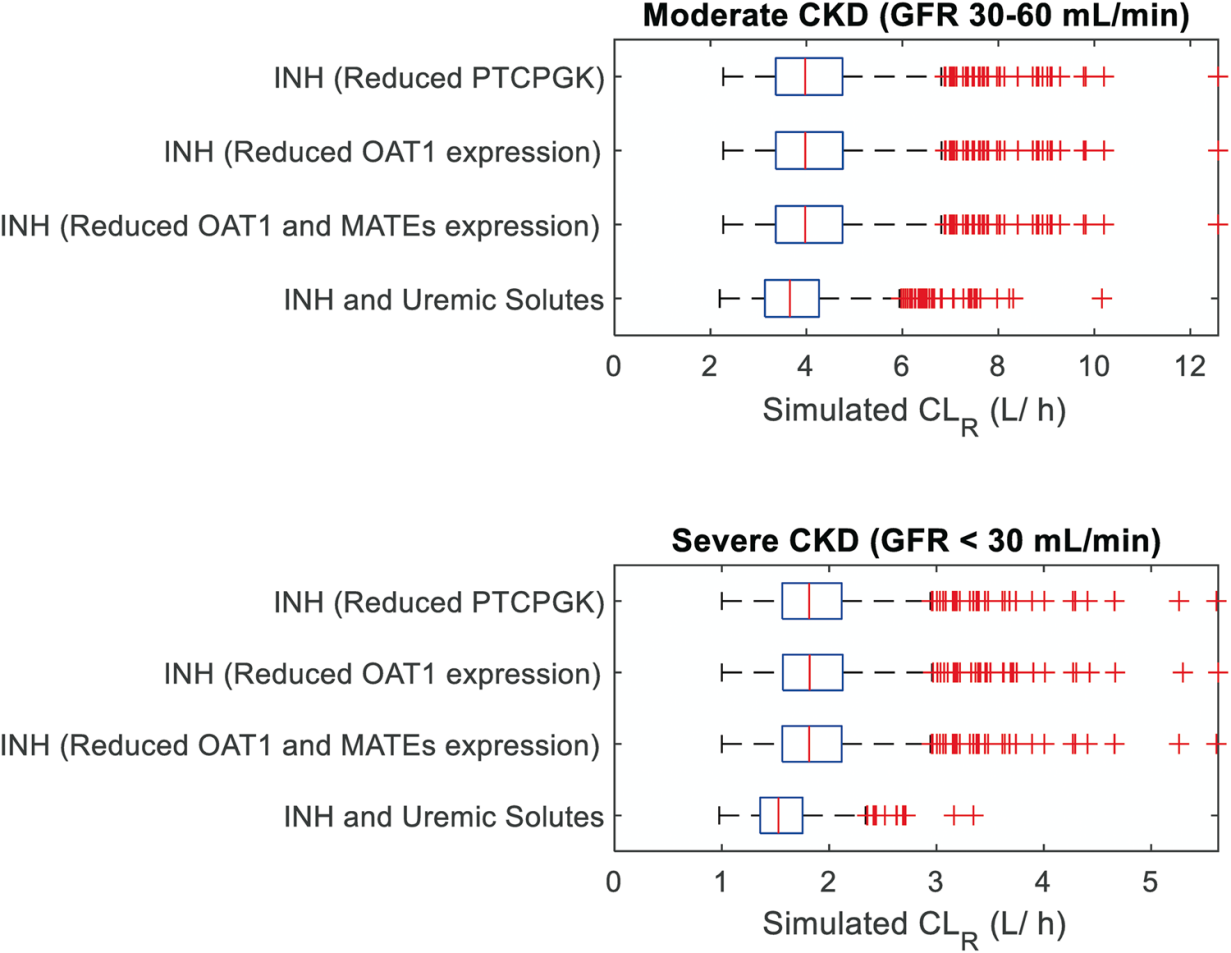
Distributions of simulated renal excretion clearance (CL_R_) in subjects with moderately or severely impaired renal function using different population models. The “INH …” population models accounted for disease related change in PTCPGK or transporter expression in accordance with the intact nephron hypothesis (INH); the “INH and Uremic Solutes” population models accounted for both INH-based reduction in PTCPGK and reduced OAT1 activity in accordance with reported inhibitory effects of uremic solutes on OAT1 in renal impairment (Table I). Simulations were performed with 1000 virtual subjects from each of the moderate (GFR 30–60 mL/min) and severe impairment (GFR < 30 mL/min) virtual populations, following the overall design of specific clinical study [53, 54]. MATE multidrug and toxin extrusion; OAT1 organic anion transporter 1

Simulated ganciclovir clearance (CL_IV_, CL_R_, and CL_PO_) using the default Simcyp CKD virtual populations under-predicted the AUC particularly for subjects with severe renal impairment (GFR < 30 mL/min) (Fig. 4). Modification of these default CKD virtual population models and inclusion of corresponding kidney systems parameters in line with the intact nephron hypothesis decreased the extent of clearance over-predictions. However, some disparity between predicted and observed CL_PO_ data still remained, in particular for the severe renal impairment sub-group (Figure S15). Between the three sets of INH-based models (GFR proportional decline in either PTCPGK, OAT1, or combined OAT1 and MATE), there were minimal differences in the simulated CL_R_; all scenarios predicted 86% decrease in median CL_R_ for severe CKD compared to healthy (Fig. 5). Consideration of differential changes in OAT1 activity (mimicking the effect of uremic solutes) together with decline in PTCPGK proportional to GFR resulted in 8% and 16% lower median simulated CL_R_ in moderate and severe renal impairment populations, respectively, compared with scenario in which only INH-based decrease in PTCPGK was considered (Fig. 5). Despite this further decrease in simulated CL_R_, consideration of both INH-based decline in PTCPGK and uremic solute-related effect on OAT1 activity did not lead to substantial improvement in predicting the overall trends in the observed plasma concentration–time data compared with the INH models (Fig. 4). As such, all CKD virtual population models were deemed plausible and considered consistent with the observed ganciclovir systemic PK data, with the exception of the Simcyp default renal impairment virtual populations.

### Simulations of Ganciclovir Concentrations in the Nephron Lumen and Crystalluria Risk

The median simulated ganciclovir concentrations in the lumen of each sub-region of the nephron (proximal tubule, loop of Henle, distal tubule and collecting duct) were compared following a simulation of a single dose of 900 mg valganciclovir in 100 virtual subjects from the “Healthy Volunteers” population. Simulated ganciclovir tubular concentrations were the highest in the medullary collecting duct (Figure S16), with *C*_max_ > 130-fold higher than in the proximal tubule. Therefore, subsequent simulations performed to assess the potential risk of crystalluria associated with either age, CKD, or variable fluid intake focused solely on the medullary collecting duct region of the nephron.

#### Impact of age

**Fig. 6:**
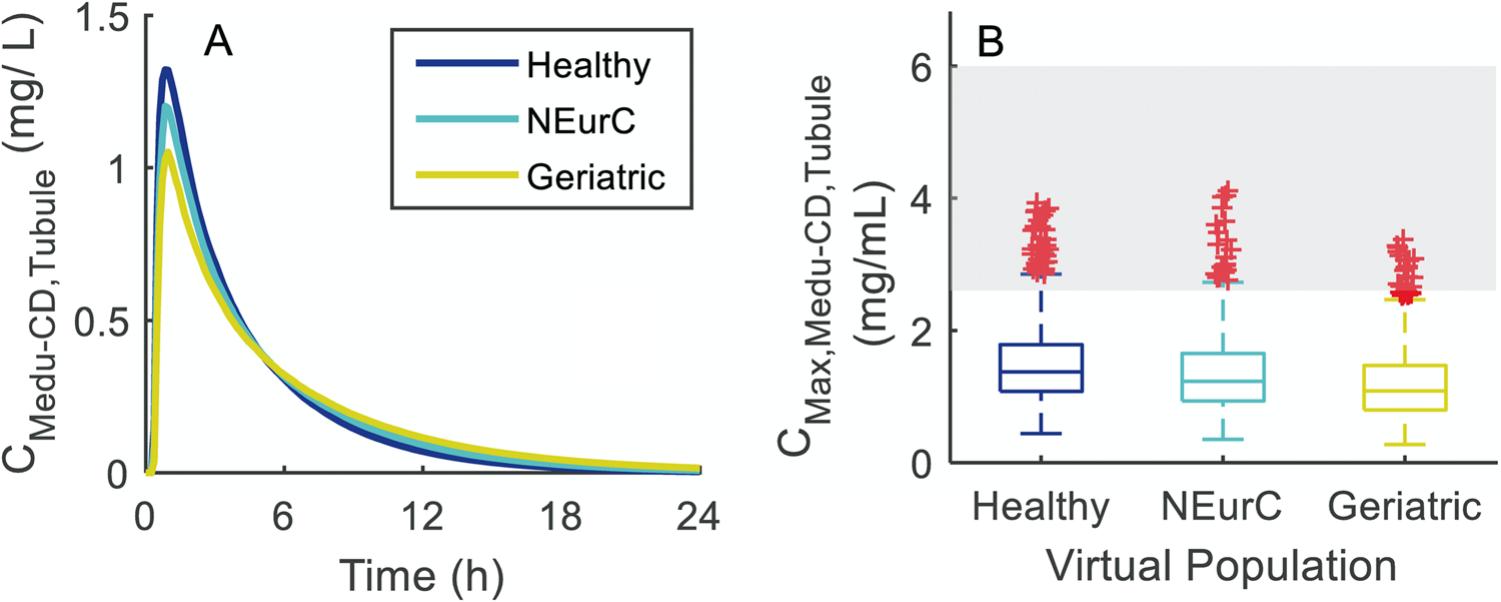
Simulated medullary collecting duct tubular concentrations (C_Medu-CD,Tubule_) of ganciclovir after single 900 mg oral dose of valganciclovir in 1000 virtual subjects from various virtual populations: younger adult (‘Healthy’), North European Caucasian (‘NEurC’), and Geriatric. **A** Lines represent median concentration–time profiles for each virtual population. **B **Boxplots of simulated maximum tubular concentrations (C_Max,Medu-CD,Tubule_) for each group of 1000 virtual subjects, compared with the aqueous solubility limit for ganciclovir (grey-shaded region; [50]). Distributions of key systems parameters of the simulation virtual subjects are presented in Figure S17; median simulated cardiac output and GFR were 19% and 45% lower in Geriatric compared with “Healthy Volunteer” virtual population

Evaluation of distributions of PBPK physiological/systems data for simulated virtual subjects from young adult (18–65 years) and geriatric (65–95 years) virtual populations revealed 19% and 45% lower median cardiac output and GFR in geriatric, while kidney weight and urine flow rate were comparable (Figure S17). Comparison of simulated ganciclovir concentrations in medullary collecting duct tubule in the young adult and geriatric virtual populations is shown in Fig. 6. As the ‘Geriatric NEC’ virtual population (65–95 years) was based upon the North European Caucasian (‘NEurCaucasian’; 18–95 years) virtual population, comparison with the latter was also performed. Overall, there was a minimal difference in the median simulated ganciclovir medullary collecting duct tubular concentrations between the healthy volunteers, “NEurCaucasian”, and geriatric virtual populations, albeit with slightly lower concentration for the Geriatric virtual population. Maximum simulated intra-tubular concentrations were typically below the aqueous solubility limit reported for ganciclovir (2600–6000 mg/L; [50]).

#### Impact of renal impairment

**Fig. 7:**
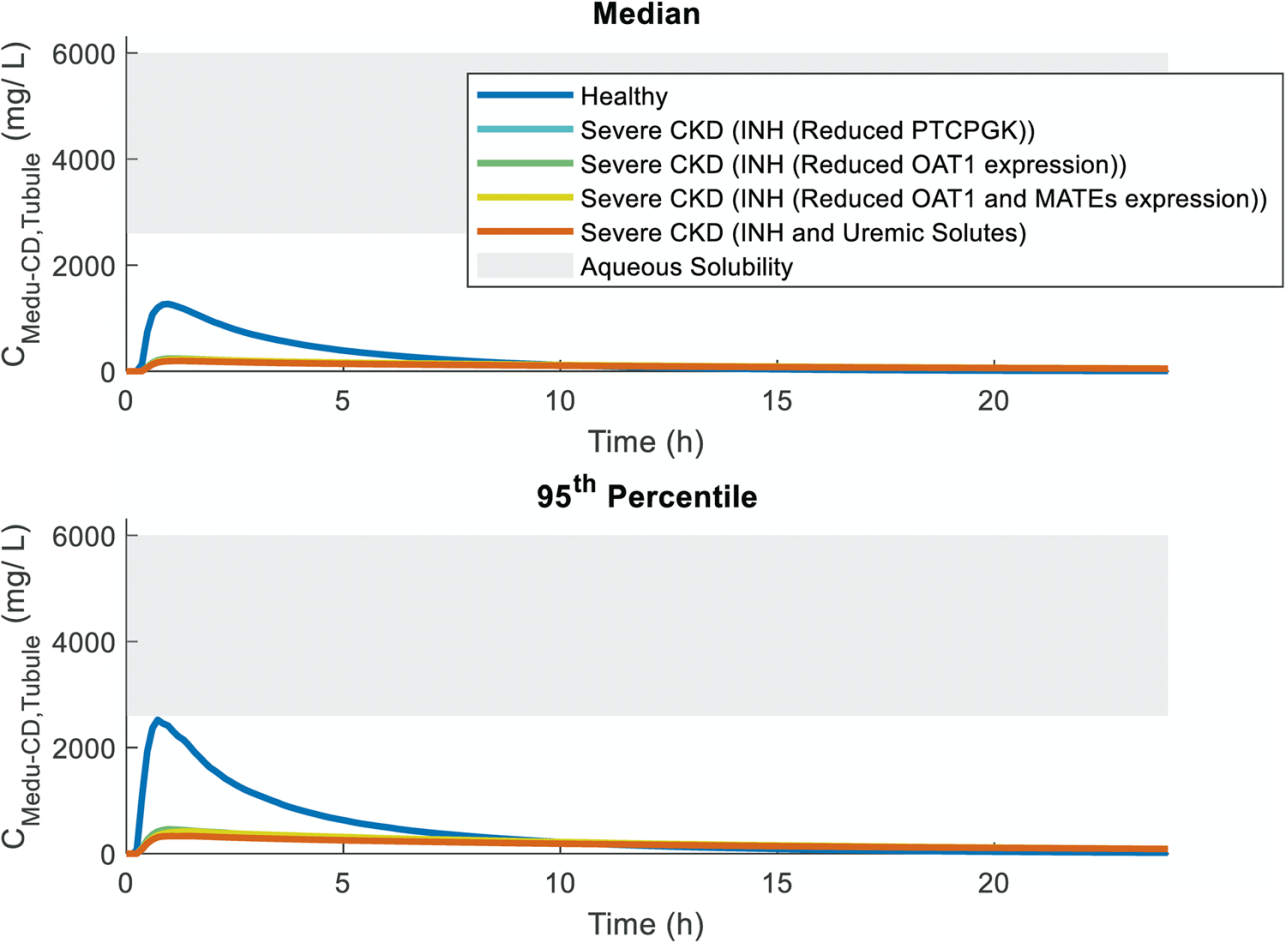
Simulated medullary collecting duct tubular concentration (C_Medu-CD,Tubule_) profiles of ganciclovir after single 900 mg oral dose of valganciclovir in various virtual populations with normal (‘Healthy’) or impaired (‘Severe CKD’; GFR < 30 mL/min) renal function. Lines each represent median or 95th percentiles of 1000 virtual subjects from each population; grey shaded area represents range of reported aqueous solubility for water and physiological conditions [50]. INH Intact nephron hypothesis; MATE multidrug and toxin extrusion; OAT1 organic anion transporter 1; PTCPGK Proximal tubule cells per gram kidney

Similar comparison was made between simulated ganciclovir concentrations in medullary collecting duct tubule in the ‘Healthy Volunteers’ and various ‘Severe CKD’ virtual populations; only those CKD population models that were consistent with the observed PK endpoint data were selected for these simulations. Overall, the median simulated tubular *C*_max_ in medullary collecting duct were up to 60% lower in the ‘Severe CKD’ populations than in the ‘Healthy Volunteers’ population when only disease-related changes in GFR were assumed. Consideration of decreased tubular secretion resulted in even lower simulated ganciclovir tubular *C*_max_ (81–85% lower than healthy), with no distinct differences between different mechanisms explored in the model (Fig. 7). Overall, median simulated tubular concentrations in medullary collecting duct in virtual CKD patients were > twofold below the range of aqueous solubility limit reported for ganciclovir (2600–6000 mg/L; [50]).

No clear intra-population relationships could be established between the high simulated ganciclovir *C*_max_ in medullary collecting duct and selected systems parameters (e.g. GFR, haematocrit, PTCPGK) (Figure S18). Notably, filtrate flow rate in medullary collecting duct was not associated with a change in maximum simulated tubular concentrations in that region under normal urine flow rate conditions, except when looking at between-population changes; these between-population changes in maximum simulated tubular concentrations were likely caused by changes in other systems parameters such as GFR.

#### Impact of dose

The recommended dose for valganciclovir for treatment of cytomegalovirus (CMV) retinitis in subjects with normal renal function is 900 mg twice daily [31]. As such, ganciclovir concentration in medullary collecting duct on the first and last day of 900 mg twice daily administration of valganciclovir for 21 days was also simulated in the healthy and renally impaired (assuming intact nephron hypothesis) virtual populations. Simulations suggest minimal ganciclovir accumulation over the treatment period in subjects with normal renal function, as the median simulated ganciclovir *C*_max_ in medullary collecting duct tubule on day 21 had increased by less than 6% compared to the first dose (Figure S19). While the corresponding changes in median ganciclovir *C*_max_ in medullary collecting duct tubule between first and last dose were 37% and 86% for the moderate and severe renal impairment, respectively, the 95th percentiles for these remained below reported aqueous solubility values (Figure S19).

#### Impact of urine flow

**Fig. 8:**
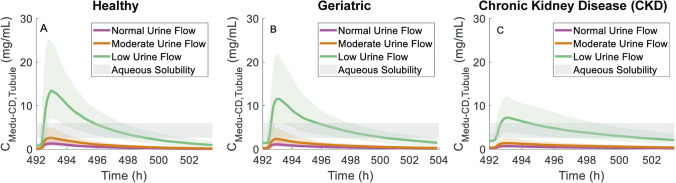
Simulated medullary collecting duct tubular concentration (C_Medu-CD,Tubule_) profiles of ganciclovir after last dose, following 21 days of twice daily 900 mg oral dose of valganciclovir, in virtual populations with normal (1 mL/min), moderate (0.5 mL/min) or low (0.1 mL/min) urine flow (UF) rates, respectively. Median and 5th and 95 percentiles were calculated from 200 virtual subjects for each urine flow rate scenario. Grey shaded area in each panel represents range of reported aqueous solubility for water and physiological conditions [50]. **A** Healthy volunteer virtual population. **B** Geriatric virtual population. **C** Chronic kidney disease population with GFR = 50 mL/min/1.73 m^2^

The virtual populations (both with normal and impaired renal function) described thus far have assumed normal urinary output (1 mL/min). However, reduced urinary output and increased susceptibility to dehydration are common in elderly subjects (among which CKD is more prevalent) [55]. Simulations of multiple dose valganciclovir regimen (900 mg twice daily) were performed in virtual subjects with normal (1 mL/min), moderate (0.5 mL/min), and low (0.1 mL/min) urine flow; selected urine flow rates were within physiologically realistic range [28]. Ganciclovir concentrations in medullary collecting duct tubule increased proportionally with decreasing urine flow rate (Fig. 8), with the median *C*_max_ at urine flow rates of 0.5 mL/min and 0.1 mL/min being twofold and tenfold higher relative to standard urine flow rate of 1 mL/min, respectively. The median simulated maximum intra-tubular concentrations (7338 mg/L) in a CKD virtual population with GFR and tubular secretion reduced by 60% compared with healthy and low urine flow rate exceeded the ganciclovir aqueous solubility threshold by > 20% (Fig. 8C), albeit to a lesser extent than for the healthy (13, 836 mg/L; Fig. 8A) and geriatric (11, 995 mg/L; Fig. 8B) virtual populations. The simulated ganciclovir intra-tubular concentrations exceeded the solubility limit at the lower urine flow rates only in the medullary collecting duct region; this trend was not evident in other regions of the nephron tubule irrespective of urine flow rate applied for simulations (simulations of other tubular regions are illustrated in Figure S20).

### Distributions of Demographic and Systems Data of Virtual Chronic Kidney Disease Patients

**Fig. 9:**
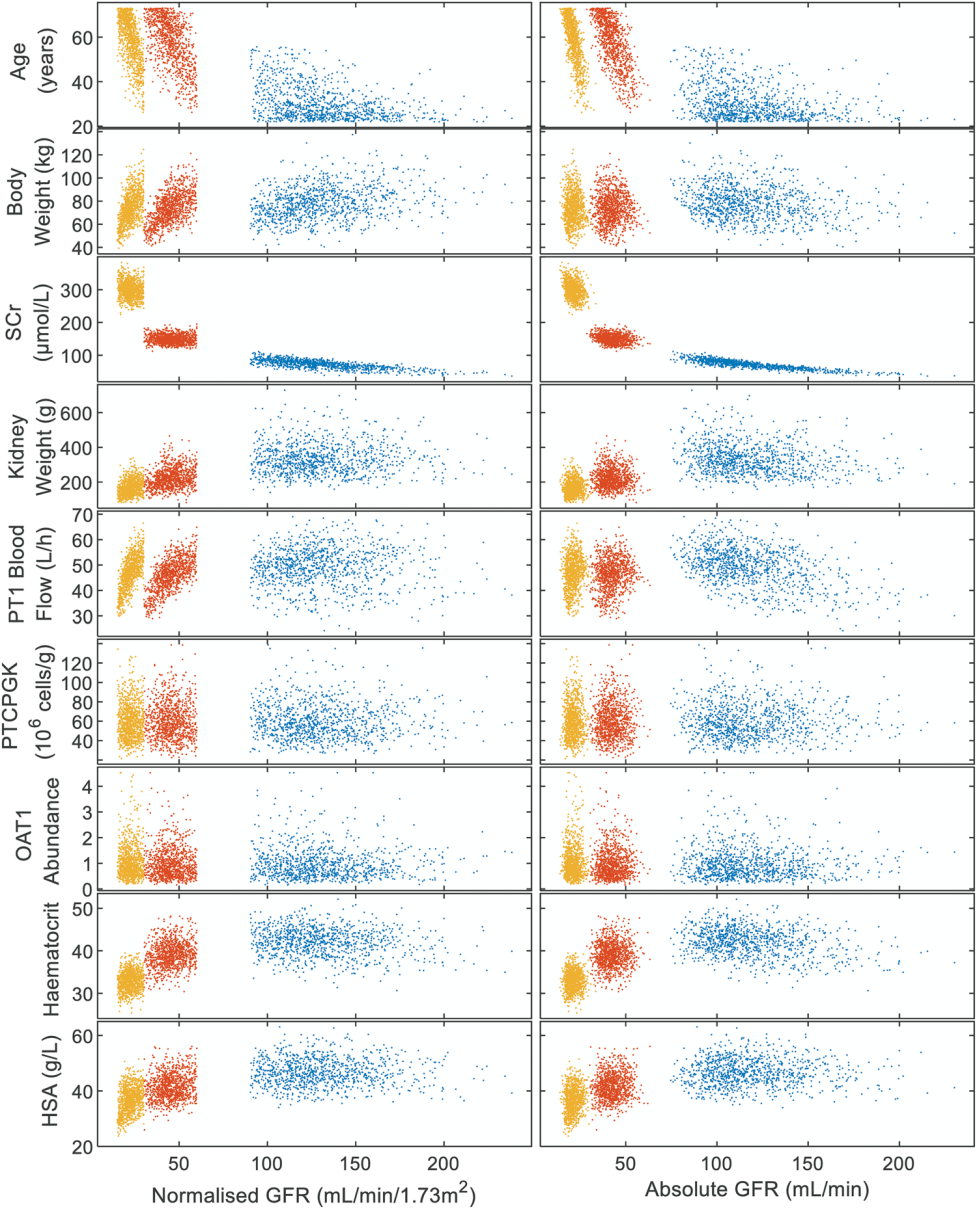
Relationships between various simulated systems parameters with either GFR normalised for body surface area (mL/min/1.73 m^2^) or absolute GFR (mL/min) for 1000 simulated subjects from Simcyp library populations “Sim-Healthy Volunteers” (blue), “Sim-RenalGFR_30-60” (red), and “Sim-RenalGFR_less_30” (yellow). CKD, chronic kidney disease; GFR, glomerular filtration rate; Hct, haematocrit; HSA, plasma concentration of human serum albumin; OAT1, organic anion transporter 1; PT1, proximal tubule, segment1; PTCPGK, proximal tubule cells per gram kidney; SCr, serum creatinine concentration

Univariate analysis indicated that distribution of parameters known to be altered with varying renal function had shifted when comparing between the populations (Figure S6). For example, median [90th percentile range] GFR in healthy and severe CKD were 114 [87.3–166] mL/min and 21.0 [16.9–26.6] mL/min, respectively; corresponding values for human serum albumin were 46.4 [38.9–54.6] µmol/L and 36.7 [29.2–44.2] µmol/L, respectively. Comparison across all different virtual populations simulated in the current study highlighted a multimodal distribution of parameters, particularly for GFR, but also for haematocrit. Bi-variate analysis revealed that as expected, within each CKD population, several systems parameters (e.g. age, proximal tubule blood flow) were correlated with GFR (Fig. 9, Figure S7). However, when comparing across the populations, gaps in the parameter space were evident that would not be expected if the moderate to severe CKD virtual populations are informed by a smooth and continuous underlying distribution of parameters.

## DISCUSSION

Increasing the use of PBPK modelling in organ impairment populations [16, 56] is captured in our comprehensive analysis of published examples (Fig. 1), with key points of individual studies summarised in Supplementary Table S2. Despite these encouraging research trends, organ impairment was still a relatively minor application in PBPK submissions to US FDA in 2018–2019 [57]. The latest FDA guidance on renal impairment advocates the use of PBPK modelling for early characterisation of the impact of renal impairment on drug pharmacokinetics, to enable inclusion of CKD patients in pivotal clinical trials [5]. Several studies in the literature trend analysis (Fig. 1) used mechanistic kidney models to explore the impact of impaired kidney function, focusing mainly on changes in systemic drug or biomarker concentrations [14, 23, 26, 58, 59]. Evidence of simulations of intra-tubular drug concentrations and/or comparison of these with drug solubility for crystalluria risk assessment were sparse [27–29]. However, none of these studies used modelling and simulations to explore relevance of CKD (in isolation and in combination with other risk factors) on the intra-tubular drug concentrations. Clinical drug concentration data that would allow direct evaluation and validation of these model-based simulations of tissue exposure are not available, as measurement of intra-tubular concentrations in human is not feasible. In such cases, indirect approaches (e.g. availability of PD data or data on the incidence of toxicity associated with a relevant tissue) can be used for validation of PBPK simulations of tissue exposure, as done previously for simvastatin [60] and simeprevir [16].

The current study aimed to simulate the effect of perturbations in renal physiological/system parameters caused by CKD, on the systemic and intra-tubular exposure of ganciclovir, building upon previous research that predicted renal clearance for tubular reabsorbed drugs [28, 61] and PBPK-CKD models developed for drugs with transporter-mediated renal disposition [14, 23, 26]. In addition, the effect of age and low fluid intake were assessed in isolation and in combination with disease-related changes implemented in ganciclovir PBPK model. Ganciclovir was selected due to clinical relevance of the scenarios investigated in the PBPK modelling, namely reported crystalluria risk. In addition, clinical plasma PK data for ganciclovir were available in subjects with both normal and impaired renal function after intravenous and oral drug administration.

In the case of drugs that are extensively excreted unchanged in urine or actively secreted (such as ganciclovir), modification of PBPK models to capture disease-related changes in expression/functional activity of renal transporters in proximal tubule cells is challenging, due to existing gaps in quantitative understanding of such physiological changes in patients with impaired renal function [18, 48]. Nevertheless, previous studies have used mechanistic PBPK kidney models to explore CKD-related changes on renally eliminated drugs. Majority of these CKD PBPK models have focused on OAT1/3 substrates (e.g. adefovir, oseltamivir carboxylate [23, 62]), with also some reports for substrates for OCT2/MATEs (e.g. metformin, atenolol, creatinine [26, 46, 63]) and OATP4C1/ P-gp (e.g. digoxin [14]). Some of these efforts used available clinical plasma and/or urinary drug concentration data to derive disease-related changes in physiological/systems parameters (e.g. changes in transporter protein abundance) [25, 46, 62]. Similar reverse translational approaches were applied in the current study, with an operational secretion model that did not distinguish the role of individual OAT transporters due to lack of informative data.

Step-wise PBPK model development and validation applied here allowed extension of ganciclovir model from healthy to CKD population, with consideration of additional covariates such as age and urine flow. Multiple possible CKD mechanisms of altered ganciclovir tubular secretion implemented in the model (decrease in proximal tubule cellularity, OAT1 abundance or OAT1/MATEs in proportion to the disease-related decrease in GFR, or with additional effect of uremic solutes on OAT1 activity) resulted in comparable performance and agreement with the observed ganciclovir systemic PK data. These findings are consistent with the empirical analyses reported in the Valcyte FDA Clinical Pharmacology Review [53], where a power model (i.e. deviation from proportionality) was only slightly better than linear model (consistent with INH) in describing relationship between ganciclovir oral clearance and creatinine clearance. Recent broader analyses suggest that INH-based approach may not be appropriate to inform dose adjustment in renal impairment patients [17, 22]. For drugs with a larger contribution of secretion to the total clearance than ganciclovir, the distinction between INH and non-INH assumptions in CKD-PBPK predictions is likely to be more evident. As there is still uncertainty arising from variable clinical data [19] and gaps in systems data [18, 64], the pragmatic approach taken here was to test a variety of CKD model assumptions in combination with parameter sensitivity analysis [65].

Predicted intra-tubular ganciclovir concentrations showed similar sensitivity to assumptions of reduced proximal tubule cellularity or reduced OAT1 abundance as mechanisms causing impaired active secretion in chronic kidney disease. In contrast, digoxin PBPK modelling in CKD demonstrated that disease-related change in OATP4C1 transporter abundance/activity and in proximal tubule cellularity resulted in significantly different predictions of digoxin concentrations in proximal tubule cells [14], although these disease-related changes could not be differentiated when predicting systemic exposure of digoxin. Consideration of disease-related decrease in MATE abundance had minimal impact on predicted systemic pharmacokinetics of ganciclovir in CKD, consistent with lack of sensitivity of MATE CL_int,u_ to *C*_max_ in medullary collecting duct (Supplementary material, Sect. 6). These results indicate that uptake into proximal tubule is the rate determining step for ganciclovir secretion. It contrasts with previous PBPK modelling of MATE and OCT2 substrates (e.g. metformin, creatinine) where the bidirectional, electrochemical gradient-driven OCT2 transport led to sensitivity of systemic drug concentrations to MATE transport rates [26, 43, 66].

Simulated ganciclovir exposure in the medullary collecting duct in CKD was > twofold lower than in healthy volunteers, under condition of normal urine flow. The geriatric model accounted for age-related changes in GFR, but unlike CKD, there were no age-related changes in tubular secretion. This resulted in modest age-related reduction in ganciclovir CL_R_ (~ 33% lower) in geriatric compared with healthy volunteers and marginal change in simulated medullary collecting duct concentration. As such, the expected risk of ganciclovir crystalluria does not increase for the geriatric and CKD populations compared to young adults with normal renal function (assuming normal urine flow rate). However, the PBPK simulations identified that low fluid intake in either older age or CKD patients increases the risk of ganciclovir-induced crystalluria. High predicted crystalluria risk under condition of decreased fluid intake/urine flow was also evident in simulations for healthy virtual subjects (normal renal function), consistent with clinical data [27, 28, 31, 32]. Further work is needed to confirm whether such findings extend to drugs with greater membrane permeability than ganciclovir. In the current study, solubility data measured in human urine were not available for ganciclovir unlike previous simulation study that investigated drug-induced crystalluria [29]. The aqueous solubility of ganciclovir (pK_a_ 2.2 and 9.4 [50]) is expected to be similar across the physiological urine pH range reported for health and disease states (pH 4.5–8.0) [67], although the potential for urinary salts to modulate ganciclovir solubility in urine compared to water cannot be ruled out. Mechanistic modelling of the precipitation processes could be considered to extend this research towards quantitative evaluation of drug-induced nephrolithiasis.

Increasing use of PBPK models highlights the necessity for robust mechanistic kidney models and virtual disease populations to support evaluation of pharmacokinetics in untested scenarios (e.g. disease-drug-drug interactions). Assessment of distributions of demographic and systems data of virtual CKD subjects, indicated potential misspecification of these virtual patient populations. For example, a number of virtual subjects aged 30–40 years had simulated GFR in ranges 25–30 and 40–60 mL/min/1.73 m^2^, yet between these groups (i.e. GFR 30–40 mL/min/1.73 m^2^), there were almost no subjects within this age band (Figure S7). Such non-continuous multimodal distribution is unlikely in reality. As such, further refinement of chronic kidney disease virtual population is required in order to describe disease-related changes in physiological/system parameters as continuous functions. In the current study, the misspecification of the virtual population prevented simulation of reported study design and direct comparison of simulated and observed data for some of the CKD patients. Despite these limitations, simulations using virtual CKD populations were deemed useful for exploration of potential risk factors on changes in systemic and tubular exposure, either in isolation or in combination.

## CONCLUSION

The current study has illustrated the use of mechanistic kidney models to evaluate multiple clinically relevant scenarios, individually or in combination. Interpretation of simulated tubular concentrations and solubility enable evaluation of crystalluria risk in these clinical scenarios and subsequently allow indirect verification of PBPK simulations against clinical reports. Knowledge gaps in CKD virtual populations were identified, highlighting that further work is needed for refinement of virtual disease populations to support evaluation of untested scenarios, including evaluation of the effect of multiple covariates (as done here) or model-based assessment of DDI risk in such patients.

## Supplementary Information

Below is the link to the electronic supplementary material.

Supplementary file1 (DOCX 6101 KB)
