# Supplementary material for: PBPK Simulation-Based Evaluation of Ganciclovir Crystalluria Risk Factors: Effect of Renal Impairment, Old Age, and Low Fluid Intake
Source: AAPS J. 2021 Dec 14;24(1):13. doi: 10.1208/s12248-021-00654-1 (PMC8816528; doi:10.1208/s12248-021-00654-1)
Supplement: Supplementary file 1 — Supplementary file1 (DOCX 6101 KB) [file 12248_2021_654_MOESM1_ESM.docx]

**Supplemental material for “PBPK simulation-based evaluation of ganciclovir crystalluria risk factors: effect of renal impairment, old age and low fluid intake”**

Daniel Scotcher, Aleksandra Galetin*

Centre for Applied Pharmacokinetic Research, School of Health Sciences, University of Manchester, UK (D.S., A.G.)

* E-mail: [Aleksandra.Galetin@manchester.ac.uk](mailto:Aleksandra.Galetin@manchester.ac.uk)

**CONTENTS**

[1. Analysis of publication trends in PBPK modelling trends: Mechanistic kidney models and chronic kidney disease 2](#_Toc78139759)

[2. Observed clinical pharmacokinetic studies for ganciclovir in subjects with normal renal function 12](#_Toc78139760)

[3. Observed clinical pharmacokinetic studies and data for ganciclovir in subjects with impaired renal function 16](#_Toc78139761)

[4. Structure of permeability-limited kidney model (“MechKiM”) in Simcyp v19r1 21](#_Toc78139762)

[5. Ganciclovir PBPK parameters 22](#_Toc78139763)

[6. Sensitivity analysis 24](#_Toc78139764)

[7. Summary of relevant differences between PBPK population models 29](#_Toc78139765)

[8. Distributions and covariances of simulated systems parameters from CKD population models 33](#_Toc78139766)

[9. Simulation of ganciclovir pharmacokinetics using literature PBPK model parameters (IV) 36](#_Toc78139767)

[10. Refinement of ganciclovir PBPK model (elimination parameters) 39](#_Toc78139768)

[11. Simulation of ganciclovir systemic pharmacokinetics in subjects with normal and impaired renal function, using different population models for renal impairment 48](#_Toc78139769)

[12. Simulated ganciclovir concentrations in lumen of nephron sub-regions 49](#_Toc78139770)

[13. References 52](#_Toc78139771)

# Analysis of publication trends in PBPK modelling trends: Mechanistic kidney models and chronic kidney disease

Table S1 Inclusion/ Exclusion criteria applied in search of literature that report use of PBPK modelling with mechanistic kidney models and/ or chronic kidney disease population

| Inclusion Criteria | Exclusion Criteria |
| --- | --- |
| Relates to human | Only relevant to CKD patients on haemodialysis |
| Modelling applied to in vivo pharmacokinetics | Insufficient demographic information about CKD population(s) |
| Includes use of a (reduced or whole-body) PBPK model | Modelling applied only to in vitro or ex vivo systems |
| Full text available | Modelling does not include pharmacokinetics application (e.g., modelling of kidney (patho)-physiological processes) |
| English Language |  |

Table S2 Articles reporting use of PBPK modelling with mechanistic kidney models and/ or chronic kidney disease population

| **Modelling description/ application**  ***Drugs/ compounds of interest*** | **Software platform** | **Mechanistic Kidney Model?** | **Virtual Populations** | **Reference** |
| --- | --- | --- | --- | --- |
| Effect of altered urine pH on systemic & renal drug disposition ^a, b^  *Methamphetamine, amphetamine* | Matlab | Yes | Healthy adult only | (1) |
| Effect of altered urine flow and pH on systemic & renal drug disposition ^a, b^  *Caffeine, chloramphenicol, creatinine, dextroamphetamine, nicotine, sulfamethoxazole, and theophylline* | Simcyp | Yes | Healthy adult only | (2) |
| Creatinine-drug interactions ^c^  *Various inhibitors of renal transporters* | Matlab | Yes | Healthy adult only | (3) |
| Creatinine-drug interactions ^c^  *Various inhibitors of renal transporters* | Matlab | Yes | Healthy adult only | (4) |
| Drug-induced crystal nephropathy, rat dog human ^a^  *> 20 approved and in-house drugs* | Matlab | Yes | Healthy adult only | (5) |
| Renal excretion clearance prediction (*46 drugs)*  Effect of urine flow/ pH on renal clearance (*Memantine, salicylic acid*) ^a^ | Matlab | Yes | Healthy adult only | (6) |
| Pharmacogenomic of OATP1B transporters  *Rosuvastatin* | Simcyp | Yes | Healthy with OATP1B1 polymorphisms | (7) |
| Effect of urine flow on renal excretion clearance ^b^  *Butabarbital, chloramphenicol, creatinine, ethanol, theophylline, and urea* | PROPHET system | Yes | Healthy adult only | (8) |
| Effect of urine flow on renal excretion clearance ^b^  *Theophylline* | PROPHET system | Yes | Healthy adult only | (9) |
| Description of kidney model and systems data | Simcyp | Yes | Healthy adult only | (10) |
| DDI involving CYP3A4, BCRP and OAT1 ^c^  *Fimasartan, hydrochlorothiazide* | Simcyp | Yes | Healthy adult only | (11) |
| PBPK-PD for SGLT2 inhibitors, effect on urinary glucose excretion ^a^  *Dapagliflozin, canagliflozin and empagliflozin* | R | Yes | Healthy adult; type 2 diabetes mellitus patients | (12) |
| DDI involving OCT2 and MATE transporters ^c^  *Cimetidine, metformin* | Simcyp | Yes | Healthy adult only | (13) |
| DDI involving OAT transporters ^c^  *Pemetrexed, NSAIDs* | Simcyp | Yes | Healthy adult only | (14) |
| DDI involving OAT transporters ^c^  *In-house compound, probenecid, tenofovir and ciprofloxacin* | Simcyp | Yes | Healthy adult only | (15) |
| PBPK-PD for SGLT2 inhibitor, effect on urinary glucose excretion ^a^  *Canagliflozin* | Simcyp | Yes | Healthy adult; diabetes patients | (16) |
| DDI involving OAT3 transporter ^c^  *Baricitinib, probenecid, ibuprofen, diclofenac* | Simcyp | Yes | Healthy adult only | (17) |
| DDI involving OCT/ MATE transporters  *Cimetidine, metformin* | Numeric Analysis Program for Pharmacokinetics | Yes | Healthy adult only | (18) |
| Toxicokinetics  *Perfluorooctanoic acid* | AcslX | Yes | General US population | (19) |
| Toxicokinetics  *Perfluorooctane sulfonate* | R | Yes | Healthy adult only | (20) |
| DDI in paediatrics ^c^  *Methotrexate and 6-mercaptopurine* | Matlab | Yes | Healthy adult; paediatric | (21) |
| DDI in paediatrics ^c^  *Methotrexate and 6-mercaptopurine* | Matlab | Yes | Healthy adult; paediatric | (22) |
| DDI involving OAT transporters ^c^  *Tenofovir, probenecid* | Simcyp | Yes | Healthy adult only | (23) |
| Pregnancy  *Tenofovir, lamivudine, emtricitabine* | Simcyp | Yes | Pregnancy | (24) |
| Alzheimer’s disease  *Digoxin* | Simcyp | Yes | Healthy adult; geriatric | (25) |
| Prediction of human PK  *Training and test sets of 69 and 18 compounds, respectively* | R | Yes | Healthy adult | (26) |
| PBPK-PD for SGLT2 inhibitors, effect on urinary glucose excretion  *Dapagliflozin* | PK-Sim | Yes | Healthy adult; diabetes patient | (27) |
| PBPK-PD for SGLT2 inhibitors, effect on urinary glucose excretion  *Dapagliflozin, canagliflozin, ipragliflozin, empagliflozin, and tofogliflozin* | DBSolve Optimum | Yes | Healthy adult | (28) |
| Effect of DDI on systemic disposition by inhibitor of OAT3, UGT1A9, MRP4 and OATP1B1  *Probenecid, furosemide and rifampicin* | PK-Sim | Yes | Healthy adult | (29) |
| PBPK-PD for SGLT2 inhibitors, effect on urinary glucose excretion  *Sitagliptin, teneligliptin, canagliflozin, dapagliflozin, ipragliflozin* | Simcyp | Yes | Healthy adult | (30) |
| Effect of DDI and food effects for OAT1/3, BCRP, MRP2/4, OATP2B1 substrate  *Furosemide, probenecid* | Simcyp | Yes | Healthy adult | (31) |
| Inter-species extrapolation of drug concentration in tissues  *Metformin* | COPASI | Yes | Pre-clinical species; healthy human adult | (32) |
| Effects of DDI and food effects on PK  *Furosemide, probenecid* | R | Yes | Healthy adult | (33) |
| Complex drug-drug-disease interactions ^c^  *Rivaroxaban* | Simcyp | Yes | Healthy adult; CKD | (34) |
| Effect of CKD and DDI on systemic and renal disposition for OAT substrates ^a, c^  *Oseltamivir carboxylate, cidofovir and cefuroxime* | Simcyp | Yes | Healthy adult; CKD | (35) |
| Effect of hepatic and renal impairment on PK for OCT/ MATE substrate  *Metformin* | Simcyp | Yes | Healthy adult; CKD; hepatic impairment | (36) |
| Effect of CKD and DDI on systemic and renal disposition for OATP4C1 and P-gp substrate ^a, c^  *Digoxin* | Simcyp | Yes | Healthy adult; CKD, geriatric | (37) |
| Effect of CKD and DDI on systemic PK of OAT substrates ^c^  *Adefovir, avibactam, entecavir, famotidine, ganciclovir, oseltamivir carboxylate, and sitagliptin* | Simcyp | Yes | Healthy adult; CKD | (38) |
| Effect of CKD on systemic PK of OCT/ MATE substrate  *Metformin, ranitidine* | Simcyp | Yes | Healthy adult; CKD | (39) |
| PBPK-PD for anticoagulant; effect of CKD  *Idarucizumab, dabigatran* | PK-Sim | Yes | Healthy adult; CKD | (40) |
| Effect of CKD on urinary and systemic disposition  *Renally excreted/ reabsorbed drugs (n=20)* | Matlab | Yes | Healthy adult; CKD | (41) |
| DDI and effect of CKD on PK of substrate for OATP1B and OAT3 ^c^  *Cyclosporine and development compound* | Simcyp | Yes | Healthy adult; CKD | (42) |
| Creatinine-drug interactions in CKD ^c^  *Creatinine, trimethoprim, cimetidine, famotidine* | Matlab | Yes | CKD | (43) |
| Complex drug-drug-disease interactions ^a, c^  *Veliparib* | Simcyp | Yes | Healthy adult; CKD | (44) |
| Effect of ethnicity, DDIs, paediatrics, CKD and hepatic impairment on PK of CYP2D6 substrate ^c^  *Atomoxetine* | Simcyp | No | Healthy adult (Chinese, Japanese, and Caucasian); CKD; hepatic impairment; | (45) |
| Effect of CKD and hepatic impairment on PK of CYP substrates  *Clozapine, sildenafil* | Simcyp | No | Healthy adult; CKD; hepatic impairment | (46) |
| Effect of CKD on PK of thiol compounds  *Captopril, tiopronin, dalcetrapib and its active form, prasugrel and its active metabolite, R-138727* | Phoenix WinNonlin | No | Healthy adult; CKD | (47) |
| Effect of CKD on PK using empirical scaling  *Isepamicin, zanamivir, cefepime, cidofovir, enprofylline, carumonam, meropenem, tomopenem, cefotetan, batanopride, cyclophosphamide, lidocaine* | Phoenix WinNonlin | No | Healthy adult; CKD | (48) |
| PK prediction in Parkinson’s disease patients with CKD for OCT2 substrate  *Pramipexole* | GastroPlus | No | Healthy adult; CKD | (49) |
| PK prediction in paediatric patients with CKD for renally excreted drug  *Ertapenem* | GastroPlus | No | Healthy adult; paediatric; CKD | (50) |
| Effect of CKD on PK for renally excreted drugs  *4 FDA cases + oseltamivir carboxylate, cidofovir, cefuroxime* | Simcyp | No | Healthy adult; CKD | (51) |
| Effect of CKD on PK for renally excreted drug  *Ceftazidime* | Simcyp | No | Healthy adult; CKD | (52) |
| Effect of CKD on PK for CYP2C8 and OATP1B substrates  *Rosiglitazone, pioglitazone, pitavastatin, repaglinide* | Simcyp | No | Healthy adult; CKD | (53) |
| Effect of CKD on PK for metabolised drugs  *Nifedipine, sildenafil, zidovudine* | Simcyp | No | Healthy adult; CKD | (54) |
| Effect of CKD on systemic PK of OCT/ MATE substrate  *Metformin* | GastroPlus | No | Healthy adult; CKD | (55) |
| Effect of CKD and DDI on systemic PK of OCT/ MATE substrate ^c^  *Metformin, cimetidine* | PK-Sim | No | Healthy adult; CKD | (56) |
| Effect of CKD and P-gp mediated DDI on systemic PK ^c^  *Dabigatran (etexilate), verapamil, quinidine* | Simcyp | No | Healthy adult; CKD | (57) |
| Effect of CKD on systemic PK for drug that undergoes hepatic metabolism and renal excretion  *Bisoprolol* | GastroPlus | No | Healthy adult; CKD | (58) |
| Effect of CKD on systemic PK for non-renally eliminated drugs  *Sildenafil, repaglinide, and telithromycin* | Simcyp | No | Healthy adult; CKD | (59) |
| Review of research to assess effect of CKD on systemic drug PK. Case study with PBPK  *Clarithromycin* | Not specified | Not specified | Healthy adult; CKD | (60) |
| Effect of CKD on systemic PK for non-renally eliminated drug  *Irinotecan and metabolite SN-38* | Napp nonlinear regression analysis  program | Not specified | Healthy adult; CKD | (61) |
| Review of systems data for CKD and application to case studies  *Paroxetine, diltiazem, repaglinide* | Simcyp | No | Healthy adult; CKD | (62) |
| Effect of CKD on systemic PK for CYP substrate  *Orteronel* | Simcyp | No | Healthy adult; CKD | (63) |
| Complex disease-drug-drug interaction in CKD patients with CYP3A/P-gp inhibitor ^c^  *Rivaroxaban, erythromycin* | SAAMII/ Simcyp | No | Healthy adult; CKD | (64) |
| Effect of ethnicity, organ impairment and PGx on PK for CYP3A4/ OATP1B substrate  *Simeprevir* | Simcyp | No | Healthy adult; hepatitis C virus (HCV) infected patients (Caucasian, Japanese, Chinese); CKD | (65) |
| Effect of CKD on CES/ UGT/ P-gp substrate  *Dabigatran etexilate, dabigatran, dabigatran 1-O-acylglucuronide* | PK-Sim | No | Healthy adult; CKD | (66) |
| Effect of cUTI on PK in paediatrics  *Ciprofloxacin* | PK-Sim | Unclear | Healthy adult; paediatrics with cUTI | (67) |
| Complex disease-drug-drug interaction in CKD patients with CYP3A/UGT2B7 substrate ^c^  *Mirabegron, itraconazole, desipramine* | Simcyp | No | Healthy adult; CKD | (68) |
| Complex disease-drug-drug interaction in CKD patients with CYP3A/ P-gp inhibitors ^c^  *Rivaroxaban* | Matlab | No | Healthy adult; CKD | (69) |
| Effect of CKD on renally excreted drug  *Glycopyrronium* | Simcyp | No | Healthy adult; CKD | (70) |
| Effect of combinations of ethnicity, age (paediatric), and renal function on PK of CYP3A/ P-gp substrate  *Cyclosporine* | Simcyp | No | Healthy adult (Caucasian, Japanese); paediatric; CKD; | (71) |
| Complex disease-drug-drug interaction in CKD and hepatic impairment patients with CYP/ OATP1B substrate ^c^  *Pemafibrate* | Simcyp | No | Healthy adult; CKD; hepatic impairment | (72) |
| Complex disease-drug-drug interaction in CKD patients with CYP3A4 inhibitor ^c^  *Teneligliptin* | Simcyp | No | Healthy adult (Caucasian, Japanese, Chinese); CKD; | (73) |
| Effect of CKD, hepatic impairment, and DDI on PK of CYP3A4 substrate ^c^  *Bosutinib* | Simcyp | No | Healthy adult; CKD; hepatic impairment | (74) |
| Effect of renal and hepatic impairment, and age (paediatric) on PK of renally excreted drug  *Vancomycin* | Simcyp | No | Healthy adult; CKD; hepatic impairment; paediatric | (75) |
| Complex disease-drug-drug interaction in CKD and hepatic impairment patients with CYP/ OATP1B substrate ^c^  *Pemafibrate* | Simcyp | No | Healthy adult; CKD; hepatic impairment | (76) |
| Simulation of PK in CKD patients for CYP3A substrate  *Crizotinib* | Simcyp | No | Healthy adult; CKD | (77) |
| Effect of obesity, CKD, hepatic impairment or cancer on PK of MRI contrast agent  *Gadofosveset* | Simcyp | No | Healthy adult; obesity; CKD, hepatic impairment; oncology patients | (78) |
| Effect of obesity, CKD, hepatic impairment or cancer on PK of MRI contrast agent  *Gadoteric acid* | Simcyp | No | Healthy adult; obesity; CKD, hepatic impairment; oncology patients | (79) |
| Dose adjustment for patients with CKD, hepatic impairment, and CYP3A4 polymorphism  *Quinine, ritonavir-boosted*  *lopinavir* | Matlab | No | Healthy adult; CKD | (80) |
| Effect of DDI, CKD and hepatic impairment on PK for CYP substrate ^c^  *Tofacitinib* | Simcyp | No | Healthy adult; CKD; hepatic impairment | (81) |
| Simulated drug exposure in lung in COVID-19 patients without or with CKD  *Chloroquine, hydroxychloroquine, azithromycin* | Simcyp | No | Healthy adult; CKD | (82) |
| Simulated PK in CKD of renally excreted drug  *Lamivudine* | Simcyp | No | Healthy adult; CKD | (83) |
| Complex disease-drug-drug interaction in CKD patients with CYP3A/ P-gp inhibitors ^c^  *Rivaroxaban* | Not specified | No | Healthy adult; CKD | (84) |
| Effect of age (geriatric), ethnicity, CYP genotype, CKD and hepatic impairment on PK of CYP/ UGT substrate  *Olanzapine* | Simcyp | No | Healthy adult (Caucasian, Chinese, Japanese), geriatric, CKD, hepatic impairment | (85) |
| Effect of sex, age (geriatric), obesity, ethnicity (Caucasian, Chinese, Japanese, South African), Cancer, hepatic impairment, CKD and DDI on PK of CYP2C8/ 3A4 substrate  *Dabrafenib* | Simcyp | No | Healthy adult (Caucasian, Chinese, Japanese), obese, geriatric, cancer, CKD, hepatic impairment | (86) |
| Effect of sex, age (geriatric), obesity, ethnicity (Caucasian, Chinese, Japanese, South African), Cancer, hepatic impairment, CKD and DDI on PK of CYP substrates  *Afatanib, erlotinib, gefitinib* | Simcyp | No | Healthy adult (Caucasian, Chinese, Japanese, South African), obese, geriatric, cancer, CKD, hepatic impairment | (87) |
| Effect of DDI, CKD and hepatic impairment on PK for drug that is metabolised in liver (CYP3A4) and renally excreted ^c^  *Gepotidacin* | Simcyp | No | Healthy adult; CKD | (88) |
| Predicting pharmacokinetics in patients with CKD  *Gabapentin, gentamicin, vancomycin sotalol, atenolol, pindolol, acebutolol* | PK-Sim | No | Healthy adult; CKD | (89) |
| PBPK/PD for effect of DDI and renal impairment on adverse events  *Dabigatran, ritonavir/cobicistat* | Simcyp | No | Healthy adult; CKD | (90) |
| Effect of CKD on PK  *Palbociclib* | Simcyp | No | Healthy adult; CKD | (91) |
| Effect of CKD on PK  *Anonymised drugs from IQ member companies* | Simcyp | No | Healthy adult; CKD | (92) |
| Effect of DDI, CKD and hepatic impairment on PK for CYP3A4/ P-gp substrate  *Rivaroxaban* | PK-Sim | No | Healthy adult; CKD; hepatic impairment | (93) |
| Effect of CKD on PK in Chinese population, for renally cleared drugs  *Ceftazidime, cefodizime, vancomycin, cefuroxime* | Simcyp | No | Healthy adult; CKD (Caucasian and Chinese) | (94) |
| Dose regimen design in obese subjects with varying degrees of CKD  *Telavancin* | GastroPlus | No | Obese subjects with or without CKD | (95) |
| PK in paediatric subjects with or without CKD  *Ceftazidime* | GastroPlus | No | Paediatric subjects with or without CKD | (96) |
| PK in heart failure and CKD  *Captopril* | Simcyp | No | Healthy adult, chronic heart failure, CKD | (97) |
| PK in CKD  *Teicoplanin* | Simcyp | No | Healthy adult; CKD | (98) |
| Dose optimisation in specific covid-19 patients  *Hydroxychloroquine* | Simcyp | No | Healthy adult; geriatric; hepatic impairment; CKD; pregnant women; paediatrics | (99) |
| Effect of intrinsic/extrinsic factors on repurposed COVID‐19 drugs  *Azithromycin, atazanavir, baloxavir, darunavir, lopinavir, remdesivir, ritonavir, acalabrutinib, baricitinib, ruxolitinib, dexamethasone, siltuximab, emapalumab, tocilizumab, chloroquine, hydroxychloroquine, dapagliflozin* | Simcyp | No | Healthy adult Caucasian, Japanese and Chinese; hepatic impairment; CKD | (100) |
| PK of drug and metabolites in CKD and hepatic impairment  *Selegiline and metabolites* | Simcyp | No | Healthy adult; CKD, hepatic impairment; paediatrics | (101) |
| PK in cancer, CKD and hepatic impairment  *Alectinib, Ruxolitinib, and Panobinostat* | PK-Sim | No | Healthy adult; cancer, CKD and hepatic impairment | (102) |
| Effect of CKD and/ or DDI on PK in adult and paediatric  *Apixaban, rifampicin, ketoconazole* | Simcyp | No | Healthy adult/ paediatric; CKD adult/ paediatric | (103) |

^a^ Included simulation of intra-tubule and/or intra-cellular concentrations of drug; ^b^ Simulated impact of urine flow and/ or pH; ^c^ Simulated impact of DDI; CKD Chronic kidney disease; cUTI Complex urinary tract infection; DDI drug-drug interactions; NSAID Non-steroidal anti-inflammatory drugs; PGx Pharmacogenomics; PK pharmacokinetics;

Table S3 Additional references of articles that were not included in the analysis of PBPK modelling trends ^a^

| Inclusion/ Exclusion criteria | Reference |
| --- | --- |
| Relates to human | (104-111) |
| Modelling applied only to in vitro or ex vivo systems | (112) |
| Only relevant to CKD patients on haemodialysis | (113, 114) |
| Modelling does not include pharmacokinetics application (e.g., modelling of kidney (patho)-physiological processes) | (115) |

^a^ Refer to Table S1 for inclusion/ exclusion criteria

# Observed clinical pharmacokinetic studies for ganciclovir in subjects with normal renal function

Table S4 Clinical pharmacokinetic studies for ganciclovir in subjects with normal renal function

| Route of administration | Dose  [Infusion duration] | Demographic | Number of subjects (Number Male) | Ethnicity (Study location(s)) | Age (years) | Weight (kg) | eGFR (CL_CR_) (mL/min) | PK Endpoints  [Average or individual data] | Reference |
| --- | --- | --- | --- | --- | --- | --- | --- | --- | --- |
| Intravenous  *Data were used for model refinement* | 5 mg/ kg  [1 h] | Healthy | 8 (4) | Caucasian  (Germany, England) | 44.5 ± 13.5 [22-56] | 71.3 ± 12.4  [52.4-88.5] | (93 ± 16) | Cp-t; AUC_0-∞_; CL_IV_; t_1/2_; V_d_; V_ss_; CL_R_ [Av] | (116, 117) |
|  |  | HIV,CMV | 8 (7) | Caucasian  (Germany, England) | 36.9 ± 6.01 [29-48] | 74.7 ± 7.4 [63.7-85.1] | (104 ± 17) | AUC_0-∞_; CL_IV_; t_1/2_; V_d_; V_ss_; CL_R_ [Av] |  |
|  |  | HIV,CMV | 18 (16) | (US) | [23-41] | - | - | Cp-t; AUC_0-∞_; CL_IV_; t_1/2_; V_d_; V_ss_ [Av] | (118) |
|  |  | HIV,CMV | 18 (15) | (US) | 35.2 ± 8 [22-51] | 74.7 ± 9.8 | >70 | Cp-t; AUC_0-∞_; CL_IV_; t_1/2_; V_d_ [Av] | (119) |
|  | 2.5 mg/ kg  [1 h] | HIV,CMV | 1 (1) [no.174] ^a^ | (US) | 34 | 75 | - | Cp-t | (120) |
|  | 5 mg/ kg  [1 h] | HIV,CMV | 1(1) [no.110] ^a^ | (US) | 43 | 78.7 | - |  |  |
|  |  | HIV,CMV | 12 (11) | (US) | 34.4 ± 7.65 | 59.3 ± 15.5 | - |  |  |
| Oral ^b^  *Data were used for verification of the refined model* | 360 mg | HIV,CMV | 18 (15) | (US) | 35.2 ± 8 [22-51] | (74.7 ± 9.8) | >70 | Cp-t; AUC_0-∞_; t_1/2_; C_max_, T_max_ [Av] | (119) |
|  | 900 mg | Healthy | 8 (6) | 2 Black, 6 Caucasian  (Germany, England) | 40 ± 13 | 74 ± 14 | (93 ± 16) | Cp-t; C_max_; T_max_; AUC_0-∞_; CL/F; t_1/2_; V_d_/F [Av]  FDA: CL_R_ | (116, 117) |
|  |  | HIV,CMV | 8 (7) | Caucasian  (Germany, England) | 37 ± 6 | 75 ± 7 | (104 ± 17) |  |  |
|  | 450 mg QD 3days  875 mg QD 3days  1750 mg QD 3days  2625 mg QD 3days | HIV,CMV | 20 (16) | 13 Caucasian, 6 Black, 1 Hispanic | 34 [23-46] | 73.4 [58 – 95] | (116.1 [81.4-154]) | Cp-t; C_max_; T_max_; AUC_0-24_; t_1/2_; [Av] | (121) ^c^ |

Data are reported as mean ± standard deviation [range]; AUC Area under the plasma concentration time curve; Av Average data reported; BMI Body mass index; C_max_ Maximum plasma concentration; CL_IV_ Clearance after intravenous administration; CL_R_ Renal excretion clearance; Cp-t Plasma concentration-time profile; HIV,CMV subjects seropositive for human immunodeficiency virus or cytomegalovirus; Ind Individual data reported; QD once daily; T_max_ Time at which maximum plasma concentration is observed; V_d_ apparent volume of distribution; V_ss_ volume of distribution at steady state; ^a^ Subject ID as reported in original report; ^b^  Oral dose as valganciclovir, after a meal (fed state); ^c^ Subject information is for ‘intent-to-treat’ group (n=20), as information for group completing study was not provided

Table S5 Clinical pharmacokinetic parameters for ganciclovir in subjects with normal renal function after IV administration of ganciclovir^a^

| Dose [Infusion duration] | Number of subjects | AUC_0-inf_ (mg.h/L) | CL_IV_ (L/h) | t_1/2_ (h) | V_d_ (L) | V_ss_ (L) | CL_R_ (L/h) | fe | Reference |
| --- | --- | --- | --- | --- | --- | --- | --- | --- | --- |
| 5 mg/ kg [1h] | 8 | 25.4 ± 4.3 | 14.3 ± 2.3 | 3.3 ± 0.5 | 68.4 ± 14.6 | 46.5 ± 12.1 | 12.2 ± 2.6 | - | (116, 117) |
| 5 mg/ kg [1h] | 8 | 25.4 ± 3.7 | 15.1 ± 1.7 | 3.2 ± 0.4 | 67.6 ± 3.9 | 46.4 ± 3.4 | 13.8 ± 3.2 | - |  |
| 5 mg/ kg [1h] | 18 | 22.1 ± 3.2 | 16.2 ± 2.5 | 3.3 ± 0.3 | 76.3 ± 11.9 | 52.5 ± 7 | - | - | (118) |
| 5 mg/ kg [1h] | 18 | 25.1 ± 3.8 | 15.2 ± 2.3 | 3.7 ± 0.6 | 79.2 ± 11.2 | - | - | - | (119) |

^a^ values are reported as mean ± standard deviation; AUC_0-inf_ area under the ganciclovir plasma concentration-time curve extrapolated to infinity; CL_IV_ intravenous plasma clearance; CL_R_ renal excretion clearance; fe fraction of dose excreted in urine; t_1/2_ half-life of terminal phase of plasma concentration-time profile; V_d_ apparent volume of distribution; V_ss_ volume of distribution at steady-state

Table S6 Clinical pharmacokinetic parameters for ganciclovir in subjects with normal renal function after oral administration of valganciclovir ^a, b^

| Valganciclovir Dose (mg) | Number of subjects | AUC_0-inf_ (mg.h/L) | AUC_0-24_ (mg.h/L) | t_1/2_ (h) | CL_R_ (L/h) | C_max_ (mg/L) | T_max_ (h) | CL/F (CL_po_) (L/h) | V_d_/F (L) | Reference |
| --- | --- | --- | --- | --- | --- | --- | --- | --- | --- | --- |
| 900 | 8 | 28.1 ± 5.8 | - | 3.5 ± 0.8 | 14.9 ± 3.6 | 5.8 ± 1.7 | 2 ± 1 | 24.1 ± 5.8 | 121 ± 29 | (116, 117) |
| 900 | 8 | 27.1 ± 3.5 | - | 3.8 ± 0.5 | 14.2 ± 2.3 | 5.7 ± 1.1 | 1.9 ± 0.4 | 24.2 ± 3.1 | 134 ± 27 | (116, 117) |
| 360 | 18 | 10.8 ± 1.9 | - | 3.7 ± 0.6 | - | 3 ± 0.8 | 1 ± 0.3 | - | - | (119) |
| 450 ^c^ | 20 | - | 12.7 ± 1.9 | 3.8 ± 0.8 | - | 3.3 ± 1.1 | 1.5 ^◊^ | - | - | (121) |
| 875 ^c^ | 20 | - | 24.8 ± 3.7 | 4.1 ± 0.7 | - | 6.1 ± 1.7 | 1.5 ^◊^ | - | - | (121) |
| 1750 ^c^ | 20 | - | 49.4 ± 8.9 | 4.3 ± 0.6 | - | 11.2 ± 2.8 | 2 ^◊^ | - | - | (121) |
| 2625 ^c^ | 20 | - | 74.1 ± 12.6 | 4.4 ± 0.5 | - | 15.4 ± 4.3 | 2 ^◊^ | - | - | (121) |

^a^ values are reported as mean ± standard deviation, unless otherwise specified; ^b^ Oral dose as valganciclovir, after a meal (fed state); ^c^ once daily dose over 3 days; ◊ value represents median; AUC_0-inf_ area under the ganciclovir plasma concentration-time curve extrapolated to infinity; AUC_0-24_ area under the ganciclovir plasma concentration-time curve up to 24h; C_max_ maximum observed concentration in plasma; CL/F (CL_po_) oral plasma clearance; CL_R_ renal excretion clearance; t_1/2_ half-life of terminal phase of plasma concentration-time profile; T_max_ time at which the maximum concentration in plasma was observed; V_d_/F apparent volume of distribution

# Observed clinical pharmacokinetic studies and data for ganciclovir in subjects with impaired renal function

Clinical studies and pharmacokinetic data for ganciclovir in subjects with normal and impaired renal function, taken only from reports that included subjects with impaired renal function, are listed in Table S7, Table S8, Table S9, and Table S10. These studies involved both intravenous administration of ganciclovir or oral administration of valganciclovir. Majority of subjects administered intravenous ganciclovir received a 5 mg/kg dose and were included in the analysis; data from two subjects that received different intravenous dose were excluded. All subjects receiving valganciclovir received 900 mg oral dose.

Pharmacokinetic parameters, such as AUC and CL, derived from non-compartmental analysis were preferentially collated. However, due to scarcity of available data in subjects with renal impairment, the pharmacokinetic parameters obtained from a 2-compartment model based analysis following intravenous administration of ganciclovir were included in the collated clinical data (120).

Table S7 Clinical pharmacokinetic studies for ganciclovir in subjects with impaired renal function, with corresponding information for subjects with normal renal function in those studies

| **Route of administration** | **Dose** | **Demographic** | **Number of subjects (Number Male)** | **Ethnicity (Study location(s))** | **Age**  **(years)** | **Weight (kg)** | **CLcr (mL/min)** | **SCr**  **(µmol/L)** | **PK Endpoints [Average or individual data]** | **Reference** |
| --- | --- | --- | --- | --- | --- | --- | --- | --- | --- | --- |
| **Intravenous** | 5 mg/kg ^a^ | HIV,CMV | 14 (13) | (US) | 36.1 ± 8.9 [19 – 55] | 59.4 ± 15.4 [40.0 – 85.0] | - | 67.6 ± 35.5 [17.7 – 132.6] | CL_IV_ ^b^, AUC ^b^ | (120) |
|  |  | Various CKD | 8 (6) | (US) | 38.9 ± 11.4 [23 – 55] | 65.7 ± 6.2 [60 .0– 78.8] | - | 301.7 ± 227.6 [123.8 – 804.6] | CL_IV_ ^b^, AUC ^b^ |  |
| **Oral ^c^** | 900 mg | Healthy | 8 (6) | 6 Caucasian, 2 Black (Germany, England) | 38.3 ± 13.0 [22-56] | 79.3 ± 11.3 [68.3-101.9] | 96.3 ± 15.9 [73-116] | - | Cp-t; AUC_0-∞_; CL/F; t_1/2_; CL_R_ | (116, 117) |
|  |  | HIV,CMV | 8 (7) | Caucasian (Germany, England) | 36.9 ± 6.01 [29-48] | 74.7 ± 7.4 [63.7-85.1] | 104.3 ± 17.3 [71-121] | - | Cp-t; AUC_0-∞_; CL/F; t_1/2_; CL_R_ |  |
|  |  | Mild CKD | 6 (3) | Caucasian (Germany, England) | 59.5 ± 5.4 [52-67] | 68.1 ± 6.0 [61.3-79.0] | 61.2 ± 6.3 [51-67] | - | Cp-t; AUC_0-∞_; CL/F; t_1/2_; CL_R_ |  |
|  |  | Moderate CKD | 6 (6) | 1 Black, 5 Caucasian (Germany, England) | 46.3 ± 19.1 [26-73] | 78.7 ± 9.4 [67.5-89.5] | 39.3 ± 10.3 [26-50] | - | Cp-t; AUC_0-∞_; CL/F; t_1/2_; CL_R_ |  |
|  |  | Severe CKD | 6 (6) | 1 Black, 5 Caucasian (Germany, England) | 52.0 ± 4.7 [46-60] | 74.1 ± 3.7 [68.8-7737] | (12.7 ± 2.1 [11-16]) | - | Cp-t; AUC_0-∞_; CL/F; t_1/2_; CL_R_ |  |

Data are reported as mean ± standard deviation [range]; AUC Area under the plasma concentration time curve; C_max_ Maximum plasma concentration; CL_IV_ Clearance after intravenous administration; CL_R_ Renal excretion clearance; Cp-t Plasma concentration-time profile; HIV,CMV subjects seropositive for human immunodeficiency virus or cytomegalovirus; Ind Individual data reported; QD once daily; T_max_ Time at which maximum plasma concentration is observed; V_d_ apparent volume of distribution; ^a^ One subject with normal renal function had dose of 2.5 mg/kg, and one subject with impaired renal function had dose of 1.25mg/kg; ^b^ reported PK parameters are from analysis using a 2-compartment model; Oral dose as valganciclovir, after a meal (fed state); ^c^ Subject information is for ‘intent-to-treat’ group (n=20), as information for group completing study was not provided

Table S8 Demographic for individuals with normal and impaired renal function, and corresponding ganciclovir pharmacokinetic parameters following 1 h intravenous infusion of 5 mg/kg ganciclovir (120) ^a^

| Subject ID | Renal Function | Age (y) | Gender | Weight (kg) | SCr (µmol/ L) | AUC (mg.h/L) | CL_IV_ (L/h) |
| --- | --- | --- | --- | --- | --- | --- | --- |
| 31 | Normal | 40 | M | 46.6 | 17.7 | 38.7 | 6.01 |
| 54 | Normal | 40 | M | 63 | 132.6 | 14.9 | 21.21 |
| 71 | Normal | 19 | F | 51.9 | 123.8 | 73.6 | 3.52 |
| 110 | Normal | 43 | M | 78.7 | 114.9 | 33.8 | 11.66 |
| 233 | Normal | 31 | M | 85 | 79.6 | 51 | 8.31 |
| 305 | Normal | 31 | M | 83.1 | 79.6 | 33 | 12.61 |
| 334 | Normal | 31 | M | 40 | 70.7 | 23 | 8.71 |
| 23 | Normal | 27 | M | 49 | 44.2 | 12 | 20.40 |
| 9a ^b^ | Normal | 37 | M | 45.1 | 44.2 | 12.2 | 18.48 |
| 9b ^b^ | Normal | 37 | M | 45.1 | 44.2 | 16.5 | 13.67 |
| 28 | Normal | 29 | M | 58 | 35.4 | 21.7 | 13.36 |
| 3 | Normal | 40 | M | 63 | 35.4 | 15.2 | 20.71 |
| 4 | Normal | 45 | M | 48.2 | 70.7 | 11.5 | 20.97 |
| 104 | CKD | 39 | M | 60 | 123.8 | 95.2 | 13.68 |
| 126 | CKD | 33 | M | 63.3 | 221.1 | 105 | 3.17 |
| 149 | CKD | 55 | F | 60 | 309.5 | 138.9 | 3.00 |
| 243 | CKD | 49 | F | 65 | 406.7 | 173.4 | 2.16 |
| 246 | CKD | 33 | M | 78.8 | 291.8 | 74.4 | 1.87 |
| 6 | CKD | 50 | M | 62 | 132.6 | 27.7 | 5.30 |
| 7 | CKD | 29 | M | 69 | 123.8 | 55.6 | 11.20 |

^a^ Excludes two subjects that received different doses (2.5 mg/kg and 1.25mg/kg); ^b^ Subject 9 received ganciclovir on two separate occasions.

AUC Area under the plasma concentration time curve; CL_IV_ Clearance after intravenous administration; CKD Chronic kidney disease; SCr Serum creatinine

Table S9 Creatinine clearance and ganciclovir pharmacokinetic parameters following oral administration of 900mg valganciclovir for subjects with normal and impaired renal function (116, 117). Data are listed as mean ± standard deviation

| Group | CL_CR_ (mL/min) | CL/F (L/h) | CL_R_ (L/h) | AUC_0-inf_ (mg.h/L) | t_1/2_ (h) |
| --- | --- | --- | --- | --- | --- |
| HIV,CMV | 104.3 ± 17.3 | 24.24 ± 3.15 | 14.16 ± 2.27 | 27.1 ± 3.52 | 3.83 ± 0.498 |
| Healthy ^a^ | 96.3 ± 15.9 | 24.78 ± 6.94 | 24.78 ± 2.63 | 27.8 ± 6.95 | 3.46 ± 0.657 |
| Healthy ^b^ | 96.3 ± 6.3 | 22.8 ± 2.05 | 22.8 ± 2.77 | 28.6 ± 2.86 | 3.57 ± 1.00 |
| Mild CKD | 61.2 ± 6.3 | 14.94 ± 5.98 | 14.94 ± 3.57 | 50.5 ± 23.23 | 4.85 ± 1.36 |
| Moderate CKD | 39.3 ± 10.3 | 8.16 ± 3.92 | 8.16 ± 1.61 | 99.7 ± 54.84 | 10.2 ± 4.39 |
| Severe CKD | 12.7 ± 2.1 | 2.7 ± 0.675 | 2.7 ± 0.488 | 252 ± 63 | 21.8 ± 5.23 |

^a^ Group “2G”, as reported in Table 1 of (117); ^b^ Group “2UK”, as reported in (117)

Table S10 Individual creatinine clearance (CL_CR_) and ganciclovir oral clearance (CL/F) following oral administration of 900mg valganciclovir for subjects with normal and impaired renal function (116, 117).

| CL_CR_ (mL/min) | CL/F (L/h) |
| --- | --- |
| 115.01 | 24.13 |
| 106.94 | 24.72 |
| 101.14 | 19.81 |
| 87.61 | 36.94 |
| 87.46 | 23.53 |
| 81.85 | 25.62 |
| 72.91 | 20.55 |
| 66.85 | 23.53 |
| 64.89 | 12.66 |
| 64.86 | 19.21 |
| 62.63 | 16.98 |
| 55.94 | 10.87 |
| 51.03 | 7.3 |
| 50.13 | 8.64 |
| 49.43 | 13.4 |
| 44.74 | 11.77 |
| 37.37 | 7.45 |
| 29.32 | 4.77 |
| 25.96 | 3.57 |
| 16.56 | 3.72 |
| 14.77 | 1.79 |
| 13.2 | 3.13 |
| 11.19 | 2.09 |
| 11.18 | 2.98 |

# Structure of permeability-limited kidney model (“MechKiM”) in Simcyp v19r1


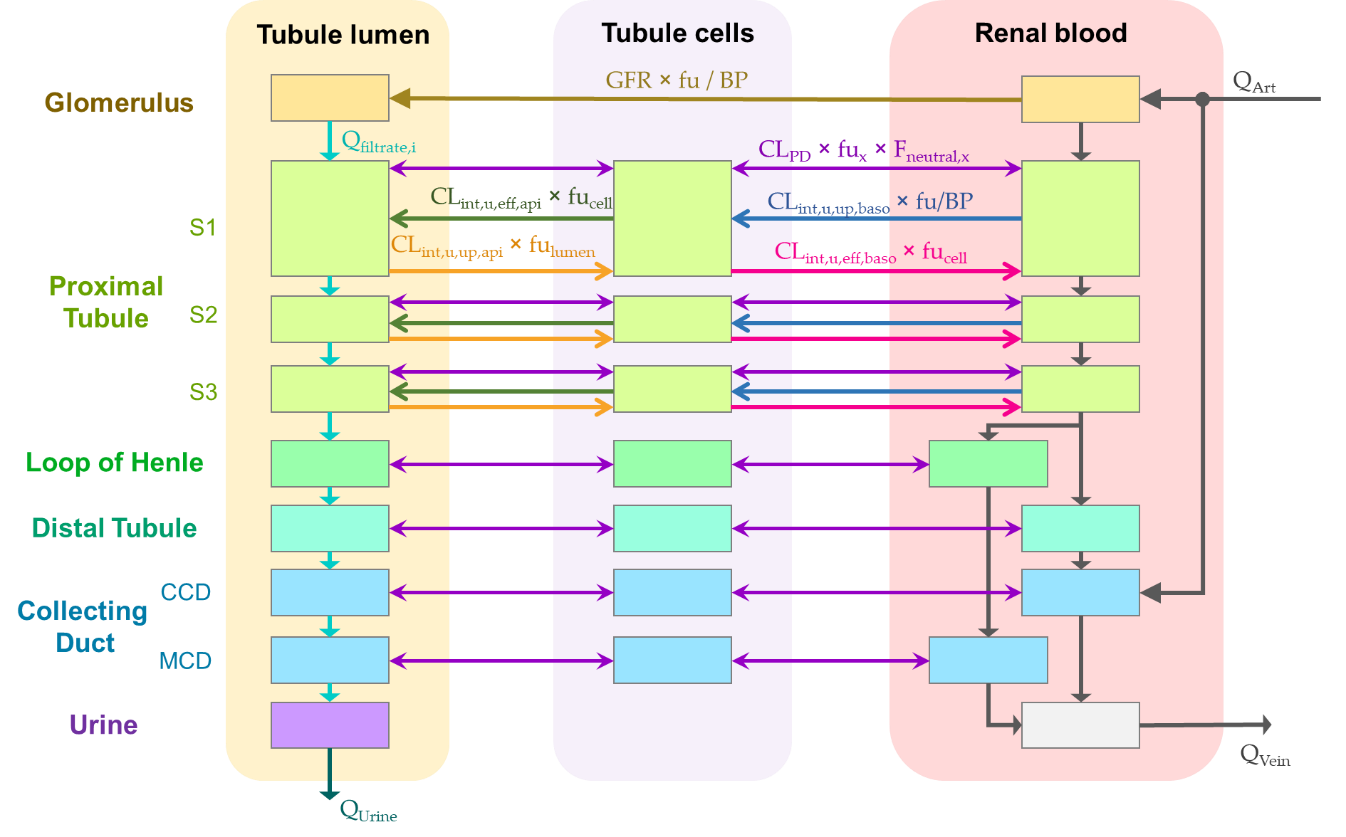


Figure S1 Schematic diagram of compartmental structure of the permeability-limited kidney model (“MechKiM” (10)) in Simcyp v19r1. Boxes represent the biological spaces and model states (well-stirred compartments). Arrows identify inter-compartment transfer rate constants as follows: Grey = blood flow rate, Brown = Glomerular filtration rate (GFR), Cyan = Tubular filtrate flow rate for i^th^ tubular region (Q_filtrate_,_i_), Purple = Passive permeability clearance (CL_PD_), Blue = intrinsic uptake transport clearance on basolateral membrane of proximal tubule cells (CL_int,u,up,baso_) [i.e., OAT1 for gansiclovir model], Green = intrinsic efflux transport clearance on apical membrane of proximal tubule cells (CL_int,u,eff,api_) [i.e., MATE1 for gansiclovir model], Orange = intrinsic uptake transport clearance on apical membrane of proximal tubule cells (CL_int,u,up,api_), Pink = intrinsic efflux transport clearance on basolateral membrane of proximal tubule cells (CL_int,u,eff,baso_). Proximal tubule is represented by three sub-region compartments (S1, S2 and S3) that are identical with exception of the tubular flow rates (Q_filtrate_,_i_), with intrinsic clearances for proximal tubule (e.g., CL_PD_, CL_int,u,up,baso_, CL_int,u,eff,api_, etc) for each compartment being 1/3 of the corresponding values scaled to total proximal tubule (see footnote ­***d*** in Table S11). Collecting duct is represented by two non-equal sub-compartments for the cortical (CCD) and medullary (MCD) regions, respectively. Passive permeability (CL_PD_) is assumed to be driven by the unbound and unionised (neutral) drug concentration in the x^th^ compartment, whereas the driving concentrations for transporter intrinsic clearance (CL_int,u,up_ and CL_int,u,eff_) are the unbound concentration in the x^th^ compartment. The permeability-limited kidney model is linked with the whole-body PBPK model through the arterial (Q_Art_) and venous (Q_Vein_) blood flows. Blood flow within the kidney incorporates bypasses to mimic anatomical arrangement of the renal circulatory system. For ganciclovir, the passive permeability is assumed to be negligible (i.e., CL_PD_ = 0 mL/min), as is transport in the direction of reabsorption (i.e., CL_int,u,up,api_ and CL_int,u,eff,baso_ = 0 mL/min). The model also has capability to incorporate metabolism in the proximal tubule cells (not shown). BP blood-to-plasma ratio; fu_x_ fraction unbound in x^th^ compartment; F_neutral,x_ fraction unionised (neutral) in x^th^ compartment; Q_urine_ urine flow rate. Figure redrawn and adapted from (13) under [CC BY 4.0 licence](https://creativecommons.org/licenses/by/4.0/).

# Ganciclovir PBPK parameters

Table S11 Parameters of ganciclovir PBPK model, implemented in Simcyp simulator (v19r1). Values were initially taken from (38) and references therein, and revisions noted in the Table and footnotes.

| **Parameter [units]** | **Value [%CV]** | **Comments** |
| --- | --- | --- |
| Mol Weight (g/mol) | 255.230 | (38) |
| log P | 1.66 | (38) |
| Compound Type | Ampholyte | (38) |
| pKa 1 (acid) | 9.4 | (38) |
| pKa 2 (base) | 2.2 | (38) |
| Blood to plasma ratio (BP) | 1 | (38) |
| Fraction unbound in plasma (fu) | 0.98 | (38)  Major binding protein: albumin  Reference albumin concentration: 45g/L ^a^ |
| Absorption model for valganciclovir | *First-order, operational model* | (38)  Mechanistic description of metabolism of the prodrug, valganciclovir, to the active moiety, ganciclovir, was beyond scope of the study; therefore an operational, first order, absorption model with lag time (TLag) was deemed sufficient as “fit-for-purpose” in the ganciclovir PBPK model, following principles of model parsimony (122). |
| Fraction absorbed (fa) | 0.61 [30%] |  |
| Lag time (h) | 0.31 [30%] |  |
| Absorption rate constant (ka) [1/h] | 2.56 [30%] |  |
| Distribution model | Whole-body PBPK | Perfusion-limited tissue compartments, with exception of kidney |
| Tissue to Plasma partition coefficients (Kp) | *Predicted using Rodgers et al method*  (123, 124)*, with a universal empirical scalar of 0.33 ^c^* | Empirical Kp scalar was revised from 0.5 in (38) to 0.33 in current study |
| Adipose | 0.861 |  |
| Bone | 1.229 |  |
| Brain | 1.255 |  |
| Gut | 1.019 |  |
| Heart | 0.489 |  |
| Kidney | 0.630 |  |
| Liver | 0.867 |  |
| Lung | 0.347 |  |
| Muscle | 0.626 |  |
| Skin | 0.699 |  |
| Spleen | 0.639 |  |
| Pancreas | 0.895 |  |
| Unbound hepatic metabolic intrinsic clearance in (CLint,u) [µL/min/mg microsomal protein] ^d^ | 0.49 [30%] | Refined by reverse translation in current study using clinical data following intravenous administration. See main text and Section 10 of supplemental material. Negligible impact on the model for oral valganciclovir (typical predicted fraction escaping hepatic metabolism was 0.98) |
| Permeability-limited kidney model (Figure S1) |  |  |
| Fraction unbound in kidney cells (fu_cell_) | 0.229 | Predicted using Rodgers and Rowland equations for intracellular water (123, 124) |
| Renal organic anion transporter (OAT)1 unbound intrinsic clearance (CLint,u) [µL/min/million proximal tubule cells] ^d^ | 6 | Refined by reverse translation in current study using clinical data following intravenous administration. See main text and Section 10 of supplemental material.  *Defines uptake at basolateral membrane* |
| Renal multidrug and toxin extrusion (MATE)1 unbound intrinsic clearance (CLint,u) [µL/min/million proximal tubule cells] ^d^ | 1 | Arbitrary value as parameter not identifiable in current analyses. See main text and Section 10 of supplemental material.  *Defines efflux at apical membrane* |
| Passive permeability across nephron tubular cells [mL/min/million tubule cells] ^d^ | 0 | (38)  Assumed negligible |

^a^ Individual fu recalculated on basis of albumin concentration in that individual and calculation of dissociation constant (K_D_) from the reference fu and reference albumin concentration ([HSA]), assuming one binding site, no binding to other plasma constituents, and no change in K_D_ between subjects ($fu=\frac{1}{1+\frac{[HSA]}{K_{D}}}$ );

^b^ Hybrid parameter taking into account passive permeability and villous blood supply (125);

^c^ Scaling factor for tissue Kp was reported as 0.5 in (38), but was set to 0.33 in the file obtained from the Simcyp online repository, so both values were tested against intravenous clinical data (not shown), with 0.33 providing the best performance. Scaling factor of 0.33 suggests over-prediction of Kp using Rodgers and Rowland (123, 124) method, and therefore listed Kp values for specific tissues may also be biased, particularly as they cannot be independently verified. However, optimised Kp values resulted in overall good predictive performance of the Vss.

^d^ Scaled to organ level using virtual subjects’ individual physiological scaling factors (for specific values see Table 1 in main text). Individual variability and/or population differences in PTCPGK directly affect total relative transporter abundance in kidney for the individual, through the physiological scaling of transporter abundance (126). The scaling equation for OAT1 intrinsic clearance (CL_int,u,OAT1_) for the i^th^ individual (CL_int,u,OAT1,i_) is:

$Kidney {CL}_{int,u,OAT1,i} \left[ {{mL}/{min}}/{2 kidneys} \right]={CL}_{int,u,OAT1,ref} \left[ {{mL}/{min}}/{{10}^{6} PTC} \right]\times{RelAbund}_{i}\times{PTCPGK}_{i}\left[ {{10}^{6}PTC}/{g kidney} \right]\times{KW}_{i}\left[ g \right]$

where subscript “i” and “ref” indicate the individual and reference subjects, RelAbund is the relative transporter abundance, PTCPGK is the number of proximal tubule cells per gram kidney, and KW is kidney weight.

When PTCPGK is decreased in CKD according to INH, but relative abundance parameters for transporter relative to healthy are not changed (as in “INH:PTCPGK” population), then the total kidney CL_int,u,T_ for OAT and MATEs will be reduced according to INH. INH change in relative abundance (proportional to GFR) with no changes in PTCPGK results in the same outcome; therefore total kidney CL_int,u,T_ is the same for “INH:PTCPGK” and “INH: OAT1 and MATEs” populations.

# Sensitivity analysis

A post-hoc local, one-at-a-time, sensitivity analysis was performed for drug-specific input parameters of the final ganciclovir PBPK model, using the trial design of Czock et al 2002 in subjects with normal renal function (oral administration of valganciclovir) (116). Typically, local sensitivity analysis is performed by calculation of the normalised sensitivity coefficient, by determination of the partial derivative of outputs in relation to a specific input while all other inputs are fixed (127). Estimation of partial derivatives has not been demonstrated with the software platform. Sensitivity across a wider range for each drug-specific parameter, as listed in Table S11, was therefore explored. PBPK simulations of ganciclovir were performed after drug-specific parameters were increased and decreased by 25% and 50% from their final “baseline” values (i.e., after refinement), enabling calculation of apparent normalised sensitivity coefficients for those input parameters (NSC_App_; Equation S1).

| ${NSC}_{App}= \frac{\frac{Change in output}{Baseline Output}}{\frac{Change in input}{Baseline Input}}$ | Equation S1 |
| --- | --- |

NSC_App_ were calculated for key model outputs for ganciclovir (plasma AUC, plasma C_max_, CL_R_, medullary collecting duct tubule C_max_). The maximum apparent normalised sensitivity coefficient was used to classify parameters as exhibiting low (0.1 – 0.2), medium (0.2 – 0.5) or high (>0.5) sensitivity (122, 127).

All NSC_App_ are presented in Table S12. Sensitivity analysis results are also presented as spider plots (Figure S2) for those input parameters for which relative change in output parameter was > 5% over the range evaluated (i.e., where outputs exhibit sensitivity to the inputs). In addition, simulated concentration-time profiles in medullary collecting duct tubule for selected parameters are presented in Figure S3.

Uncertainty of sensitive parameters were each evaluated as being high (value could be a factor of 2 or higher), medium (value could be a factor between 0.3 and 2) or low (value could be a factor of 0.3 or lower), and the sensitivity and uncertainty together being used to evaluate reliability (122).

As expected, fa and k_a_ affected simulated plasma AUC and C_max_, and medullary collecting duct C_max_. Although k_a_ was categorised as having high sensitivity to medullary collecting duct C_max_, the NSC_App_ (0.3 – 0.54) were at the lower limit (0.5) for the high sensitivity category. fa was empirically defined in the previous version of the model (38) as a non-mechanistic model description of the conversion from valganciclovir (prodrug) to ganciclovir. Reported mean reported bioavailability data for ganciclovir following oral administration of valganciclovir across two clinical studies were consistent (range 0.59 – 0.61 (116, 117)), indicating low uncertainty for fa. The k_a_ was also an empirical parameter in the PBPK model, as mechanistic description of the valganciclovir absorption and conversion to ganciclovir was not considered necessary for the current application of the model. However, some miss-prediction of the plasma C_max_ was observed during verification of the ganciclovir model in healthy subjects due to inter-study variability in the pharmacokinetic data Figure S14 (see below). Therefore, the k_a_ parameter should be considered as having moderate uncertainty.

LogP, pKa (acid) and Kp scalar each had sensitivity toward C_max_ in plasma and medullary collecting duct (Figure S2, panel B) because these are key parameters for prediction of tissue Kp, via the Rodgers and Rowland equation (123, 124), and Kp is an important determinant of drug distribution in the current PBPK model. Most pKa measurements have a precision (standard deviation) much better than ±0.5 pKa units, while the structurally related acyclovir had pKa (acid) of 9.22±0.01, suggesting that the ganciclovir pKa (acid) parameter can be considered to have low uncertainty. The experimentally determined logP is consistent with QSAR predictions (<https://go.drugbank.com/drugs/DB01004> [accessed 1st July 2021]), and measurements are typically reproducible between laboratories e.g., (128). Thus uncertainty in logP should be considered low. Kp scalar parameter was optimised to 0.33 enable the model to fit the clinical plasma pharmacokinetic data in subjects with normal renal function, from a value of 1 which would indicate a perfect prediction using the Rodgers and Rowland method (123, 124). Therefore, Kp scalar can be considered as having moderate uncertainty.

BP and fu were sensitive parameters for the simulated pharmacokinetics of ganciclovir in plasma and the medullary collecting duct (Figure S2). fu is very close to 1, with values in this range typically being well-defined (i.e., low uncertainty). Similarly, BP value of 1 is supported by in vitro data indicating equal distribution of ganciclovir in erythrocytes and plasma (129), and therefore has low uncertainty.

The renal OAT1 CLint,u parameter exhibits moderate sensitivity to plasma AUC, CL_R_ and Cmax in the medullary collecting duct. While a high sensitivity might be expected for a renally secreted drug, ganciclovir has approximately equal contribution of secretion and filtration to the total renal secretion of ganciclovir. Nevertheless, this parameter was optimised against clinical data, and the effect of CKD on renal secretion is still a matter of ongoing research. As such, renal OAT1 CLint,u was assigned moderate uncertainty.

Overall, all parameters were considered having high or moderate reliability, with exception of ka that was categorised as low reliability (but on the borderline as moderate reliability). The uncertainty in this parameter arises predominantly from inter-individual variability (C_max_, T_max_, AUC) and apparent dose-nonlinearity (C_max_, AUC) in the pharmacokinetic data for ganciclovir following oral administration of valganciclovir (Figure S14, see below). Accounting for this inter-individual variability mechanistically within the PBPK framework would require extensive supporting data on both drug- and system- parameters, and may be considered for future work.

Figure S2. Local sensitivity analysis spider plots for drug-specific input parameters of the final ganciclovir PBPK model, using the trial design of Czock et al 2002 in subjects with normal renal function (oral administration of valganciclovir) (116). Each drug-specific parameter (P), as listed in Table S11 was increased and decreased by 25% and 50%, and the effects of these changes on ganciclovir area under the plasma concentration-time curve (AUC) [Panel A], maximum concentration in plasma (Cmax) [Panel B], and renal excretion clearance (CL_R_) [Panel C].

Figure S3. Local sensitivity analysis of simulated ganciclovir concentration-time profile for medullary collecting duct (MCD) for drug-specific input parameters of the final ganciclovir PBPK model, using the trial design of Czock et al 2002 in subjects with normal renal function (oral administration of valganciclovir) (116). Only the first 5h of simulation are shown. Each drug-specific parameter as listed in Table S11 was increased (“Forward”) and decreased (“Backward”) by 25%. Only input parameters that resulted in >10% change in concentration (at any timepoint) are presented.

Table S12 Sensitivity analysis categorisation for PBPK drug-specific input parameters using apparent normalised sensitivity coefficients (NSC_App_) for various pharmacokinetic parameters

| Sensitivity (NSC_App_ limits ^a^) | Pharmacokinetic parameters ^b^ | | | |
| --- | --- | --- | --- | --- |
|  | **Plasma AUC** | **Plasma Cmax** | **CLr** | **MCD Cmax** |
| **High**  **(>0.5)** | logP  fu  fa | logP  pKa (acid)  BP  fu  fa  Kp Scalar | fu | fa  ka  logP  pKa (acid) |
| **Moderate**  **(0.2 – 0.5)** | Renal OAT1 CLint,u | ka | BP  Renal OAT1 CLint,u | BP  Kp Scalar  Renal OAT1 CLint,u |
| **Low**  **(0.1-0.2)** | BP  Hepatic metabolic CLint,u |  |  | fu |

^a^ (122, 127); ^b^ Parameters defined in Table S11

|  | | Uncertainty | | |
| --- | --- | --- | --- | --- |
|  |  | Low | Moderate | High |
| Sensitivity | High | fa  logP | ka | - |
|  | Moderate | BP | Kp scalar  renal OAT1 CLint,u | - |
|  | Low | fu | - | - |

Figure S4. Assessment of PBPK model reliability for drug-specific input parameters. Sensitivity was evaluated for maximum concentration in the medullary collecting duct, as a primary pharmacokinetic parameter of interest to the current application of PBPK modelling (see Table S12). Reliability colour scheme: Green = high; Yellow = moderate; Orange = low; Red = very low

# Summary of relevant differences between PBPK population models

Table S13 Key systems parameters differing between the Healthy Volunteers and chronic kidney disease virtual populations of the Simcyp simulator (v19r1)

| Parameter | Virtual Population | | |
| --- | --- | --- | --- |
|  | Sim-Healthy Volunteers | Sim-RenalGFR_30-60 | Sim RenalGFR_less_30 |
| Age distribution ^a^ | User defined | Weibull | Weibull |
| CYP2C19 PM Frequency | 0.092 | 0.024 | 0.024 |
| CYP2C19 UM Frequency | 0.318 | 0 | 0 |
| Hepatic CYP3A4-CYP3A5 abundance correlation (baseline) | 62.775 | 45.47 | 34.86 |
| *Hepatic enzyme mean protein abundance (pmol/ mg protein)* | | | |
| CYP1A2 (EM) | 52 | 28.4 | 27.4 |
| CYP2A6 (EM) | 20 | 10.9 | 9.4 |
| CYP2B6 (EM) | 17 | 9.3 | 8 |
| CYP2B6 (PM) | 6 | 3.3 | 2.8 |
| CYP2C8 (EM) | 24 | 13.1 | 11.3 |
| CYP2C9 (EM) ^b^ | 73 | 39.9 | 34.5 |
| CYP2C9 (PM) ^b^ | 29 | 15.9 | 13.7 |
| CYP2C18 (EM) | 1 | 0.68 | 0.57 |
| CYP2C19 (EM) | 4.4 | 3.6 | 3 |
| CYP2C19 (UM) | 8.7 | 0 | 0 |
| CYP2D6 (EM) | 9.4 | 6 | 5.1 |
| CYP2D6 (UM) | 18.8 | 12 | 10.1 |
| CYP2E1 (EM) | 61 | 37.3 | 25.8 |
| CYP2J2 (EM) | 1.2 | 0.87 | 0.67 |
| CYP3A4 (EM) | 137 | 95.2 | 87.3 |
| CYP3A5 (EM) | 103 | 74.6 | 57.2 |
| *Serum Creatinine ^c^* | | | |
| Male Baseline 1 (µmol/L) | 76.5 | 152 | 300 |
| Male Baseline 1 CV (%) | 16.1 | 8 | 8 |
| Male Age Cut-Off (years) | 61 | 61 | 61 |
| Male Age Cut-off Baseline (µmol/L) | 81.2 | 143 | 300 |
| Male Age Cut-Off CV1 (%) | 27.4 | 8 | 8 |
| Male Age Cut-Off CV2 (%) | 21.2 | 8 | 8 |
| Female Baseline 1 (µmol/L) | 57 | 152 | 300 |
| Female Baseline CV (%) | 20.4 | 9 | 10 |
| Female Age Cut-Off 1 | 48 | 60 | 48 |
| Female Age Cut-Off Baseline 2 (µmol/L) | 66.2 | 148 | 300 |
| Female Age Cut-off 1 CV 1 (%) | 26.5 | 9 | 10 |
| Female Age Cut-off 1 CV 2 (%) | 22.8 | 9 | 10 |
| Female Age Cut-Off 2 | 78 | 75 | 78 |
| Female Age Cut-Off Baseline 3 (µmol/L) | 79.5 | 144 | 300 |
| Female Age Cut-Off 2 CV 1 (%) | 38.3 | 9 | 10 |
| Female Age Cut-Off 2 CV 2 (%) | 31.6 | 9 | 10 |
| *Other kidney parameters* | | | |
| GFR Cap Minimum Defined Population Limit (mL/min/1.73m^2^) | 90 | 30 | 15 |
| GFR Cap Maximum Defined Population Limit (mL/min/1.73m^2^) | 400 | 60 | 29.999 |
| Kidney Size Baseline ^d^ | 15.4 | 8.4 | 5.7 |
| Kidney Size BW Coeff ^d^ | 2.04 | 1.64 | 1.04 |
| Kidney Size BH Coeff ^d^ | 51.8 | 32.8 | 29.8 |
| Kidney blood flow ^e^ | Male: 19% of CO  Female: 17% of CO | Male: 19% of CO  Female: 17% of CO | Male: 19% of CO  Female: 17% of CO |
| *GI Tract enzyme mean protein abundance (pmol/ mg protein)* | | | |
| CYP2C19 UM | 4 | 0 | 0 |

^a^ Sim-Healthy Volunteers and Sim-Renal virtual populations are given different weight and height population distributions; ^b^ Population differences also applied to genotype-specific abundances; ^c^ See text below and Box S1 concerning distributions of serum creatinine; ^d^ $Kidney size = Kidney Size Baseline + Kidney Size BW Coeff\cdot BW +Kidney Size BH Coeff\cdot BH$; BH body weight; BH body height; Coeff coefficient; CV coefficient of variation; GFR glomerular filtration rate; PM Poor metabolisers; UM Ultra-metabolisers; ^e^ Cardiac output (CO) is assumed to decline linearly with age, with BSA also a covariate, therefore kidney blood flow in the Sim-Renal virtual populations are lower than for Sim-Healthy due to the older age distribution of the Sim-Renal virtual populations, although the fraction of cardiac output going to kidneys is assumed not to change.

To account for the asymmetrical distribution of serum creatinine, two coefficients of variation (CV) are provided for this parameter and relevant subjects. To allow readers to appreciate how serum creatinine parameter is simulated in Simcyp, the algorithm implemented in the software is described in Box S1 (130).

**Box S1. Algorithm for generation of serum creatinine (SCr) in virtual populations in Simcyp simulator in the i^th^ virtual subject**

IF Sex_i == Male

THEN

IF Age_i < Male Age Cut-off 1

THEN SCr_i ~ $\mathcal{N}$(Male Baseline 1, Male Baseline 1 * Male Baseline 1 CV)

ELSE

IF RandomNumber >= 0

THEN SCr_i ~ $\mathcal{N}$(Male Age Cut-off Baseline, Male Age Cut-off Baseline* Male Age Cut-Off CV1) SCr_i >= Male Age Cut-off Baseline

ELSE SCr_i ~ $\mathcal{N}$(Male Age Cut-off Baseline, Male Age Cut-off Baseline* Male Age Cut-Off CV2) SCr_i < Male Age Cut-off Baseline

END

END

ELSE [i.e., Sex==Female]

IF Age_i < Female Age Cut-off 1

THEN SCr_i ~ $\mathcal{N}$(Female Baseline 1, Female Baseline 1 * Female Baseline CV)

ELSEIF Female Age Cut-off 1 < Age_i < Female Age Cut-off 2

IF RandomNumber >= 0

THEN SCr_i ~ $\mathcal{N}$(Female Age Cut-Off Baseline 2 , Female Age Cut-Off Baseline 2 * Female Age Cut-Off 1 CV1) SCr_i >= Female Age Cut-Off Baseline 2

ELSE SCr_i ~ $\mathcal{N}$(Female Age Cut-Off Baseline 2 , Female Age Cut-Off Baseline 2 * Female Age Cut-Off 1 CV2) SCr_i < Female Age Cut-Off Baseline 2

END

ELSE

IF RandomNumber >= 0

THEN SCr_i ~ $\mathcal{N}$(Female Age Cut-Off Baseline 3 , Female Age Cut-Off Baseline 3 * Female Age Cut-Off 2 CV1) SCr_i >= Female Age Cut-Off Baseline 3

ELSE SCr_i ~ $\mathcal{N}$(Female Age Cut-Off Baseline 3 , Female Age Cut-Off Baseline 3 * Female Age Cut-Off 2 CV2) SCr_i < Female Age Cut-Off Baseline 3

END

END

Where Sex_i, Age_i and SCr_i are the sex (Male/Female), age (years) and serum creatinine (µmol/L) of the i^th^ virtual subject, and RandomNumber is a randomly generated number that can take positive and negative values. Remaining parameters are listed in TableS4.

Table S14 Examples of simulated regional tubular filtrate flow rates ^a^ (mL/min) for healthy volunteer, geriatric and chronic kidney disease subjects with normal (1 mL/min) or low (0.1 mL/min) urine flow rate.

| **Tubule Region** | **Healthy volunteers ^b^** | | **Geriatric ^b^** | | **Chronic kidney disease ^c^** | |
| --- | --- | --- | --- | --- | --- | --- |
|  | **Normal urine flow** | **Low urine flow** | **Normal urine flow** | **Low urine flow** | **Normal urine flow** | **Low urine flow** |
| Proximal tubule (S1) ^d^ | 120.0 | 120.0 | 66.0 | 66.0 | 50.0 | 50.0 |
| Proximal tubule (S2) | 94.4 | 94.2 | 52.0 | 51.8 | 39.5 | 39.3 |
| Proximal tubule (S3) | 68.8 | 68.4 | 38.0 | 37.7 | 28.9 | 28.5 |
| Loop of Henle | 43.2 | 42.7 | 24.1 | 23.5 | 18.4 | 17.8 |
| Distal tubule | 24.0 | 23.3 | 13.6 | 12.9 | 10.5 | 9.76 |
| Cortical collecting duct | 11.6 | 10.7 | 6.77 | 5.95 | 5.35 | 4.53 |
| Medullary collecting duct | 6.28 | 5.42 | 3.89 | 3.03 | 3.17 | 2.32 |

^a^ Flow rates listed are those entering the respective tubular region, corresponding to “Q_filtrate,I_” in Figure S1. The flow rate leaving the respective tubular region is equal to the flow rate entering the subsequent tubular region. i.e., the flow rate across each compartment reduces in the direction of flow, corresponding to the physiological process of fluid reabsorption that occurs along the nephron (10); ^b^ Values listed are for female virtual subjects (male values are slightly higher, e.g., GFR in male healthy volunteer was 130 mL/min); ^c^ Renal function (glomerular filtration and secretion) are 40% of healthy volunteer. This is the most severe decline in glomerular filtration rate (50 mL/min) permitted in the Simcyp software platform when urine flow is also varied; ^d^ Flow rate entering the nephron at proximal tubule (S1) is equal to glomerular filtration rate; S1, S2, and S3 refer to equal-length sub-segments 1, 2, and 3 of the proximal tubule, respectively

^
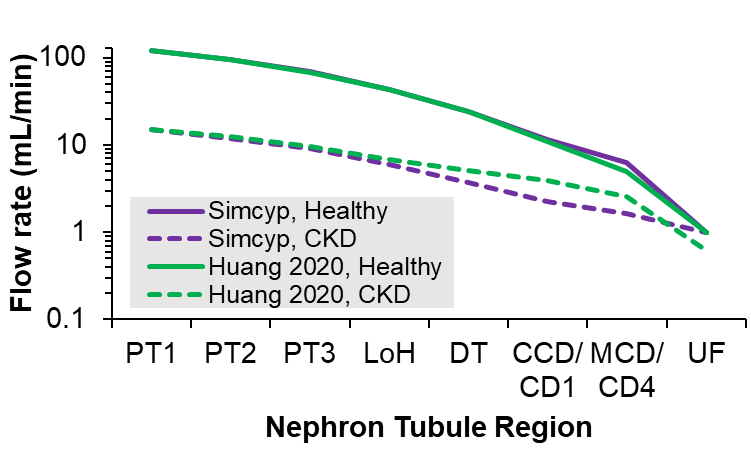
^

Figure S5 Filtrate flow rates in healthy volunteers and severe chronic kidney disease (CKD) patients in different regions of the nephron tubule, based on the models from Huang and Isoherranen 2020 (41) and the Simcyp virtual populations. In the Simcyp virtual populations, urine flow rate is assumed to be 1 mL/min in healthy and CKD populations. PT1/2/3 Proximal tubule regions 1/2/3; LoH Loop of Henle; DT Distal tubule; CCD/ MCD Cortical/ Medullary collecting duct; CD1/4 Collecting ducts regions 1/4 of Huang and Isoherranen model; UF Urine flow rate

# Distributions and covariances of simulated systems parameters from CKD population models

To compare the distributions between the populations a pragmatic, non-parametric approach was used. First, the sum of the ranked values (Rank_i_ ; within the entire sample of simulated virtual subjects), were calculated for each population (subscript x), designated U_X_ (Equation S2). Next, the ratio of summed rank (R_U_; Equation S3) between the virtual population of interest and the Healthy Volunteers population (as a reference) was calculated. An R_U_ of 1 would signify no difference between the distributions of the population of interest and the reference population for that particular parameter. R_U_ exceeding or lower than 1 indicate the parameter tends to have higher or lower values in the population of interest compared with reference population, respectively.

| $U_{X}= \sum{Rank}_{i}$ | Equation S2 |
| --- | --- |
| $R_{U,X}=\frac{U_{X}}{U_{Reference}}$ | Equation S3 |

In addition, the quartile coefficient of variation (QCV; %) was used as a descriptive statistic for the overall variability of the entire sample of simulated virtual subjects (Equation S4)

| $QCV=\frac{Q3 - Q1}{Q2}$ | Equation S4 |
| --- | --- |

Where Q1, Q2 and Q3 are the first, second and thirds quartiles, respectively.

Figure S6 Distributions of simulated systems parameters for 1000 simulated subjects from Simcyp library populations “Sim-Healthy Volunteers”, “Sim-RenalGFR_30-60” and “Sim-RenalGFR_less_30”. CKD Chronic kidney disease; GFR Glomerular filtration rate; Hct Haematorcit; HSA Plasma concentration of human serum albumin; OAT1 Organic anion transporter 1; PT1 Proximal tubule, segment1; PTCPGK proximal tubule cells per gram kidney; QCV Quartile based Coefficient of Variation; R_U,moderate_ (or R_U,Severe_) Ratio of sum of ranks between “Sim-RenalGFR_30-60” (or “Sim-RenalGFR_less30”) and “Sim-Healthy Volunteers”, respectively; SCr serum creatinine concentration


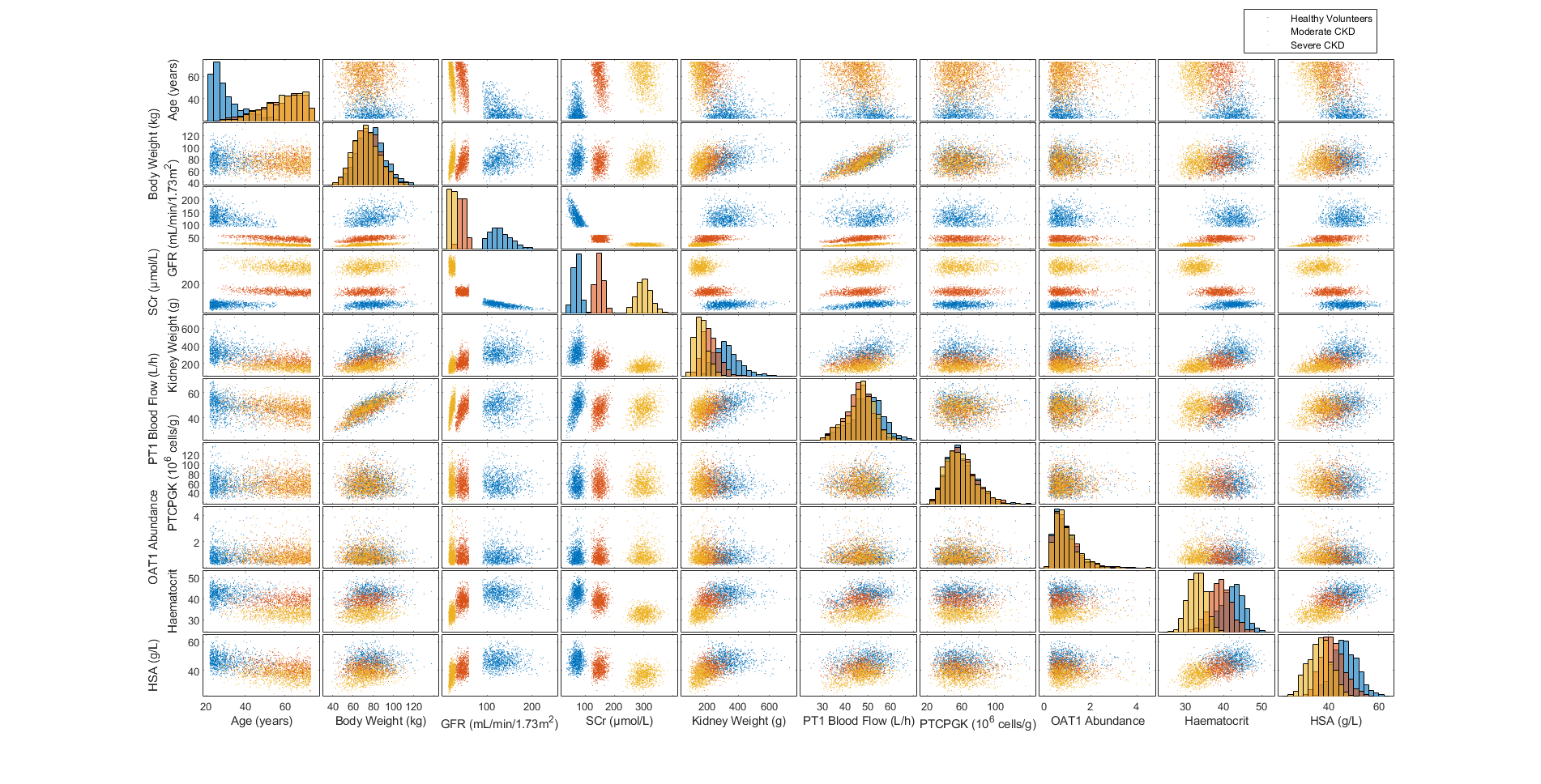


Figure S7 Pairwise relationships between simulated systems parameters for 1000 simulated subjects from Simcyp library populations “Sim-Healthy Volunteers”, “Sim-RenalGFR_30-60” and “Sim-RenalGFR_less_30”. CKD Chronic kidney disease; GFR Glomerular filtration rate; Hct Haematorcit; HSA Plasma concentration of human serum albumin; OAT1 Organic anion transporter 1; PT1 Proximal tubule, segment1; PTCPGK proximal tubule cells per gram kidney; SCr serum creatinine concentration

# Simulation of ganciclovir pharmacokinetics using literature PBPK model parameters (IV)

^^

Figure S8 Simulated gancicolivir plasma concentration-time profiles, following intravenous administration of ganciclovir, using literature PBPK model parameters (38) with comparison with observed data from literature studies. For each clinical study, 100 trials were simulated. The 5th and 95th percentiles of the simulated concentrations at each time-point for all virtual individuals were calculated (green shaded area). For studies that the observed data were reported as mean profiles, the mean concentration for all time-points were calculated for each trial, and subsequently the 5^th^ and 95^th^ percentiles of these 100 Trial Means were calculated (magenta shaded area). References and study design are listed in Table S4. C_plasma_ concentration of ganciclovir in plasma; HIV/CMV subjects seropositive for human immunodeficiency virus or cytomegalovirus; HV healthy volunteers.

Figure S9 Simulated gancicolivir plasma concentration-time profiles, following intravenous administration of ganciclovir, using literature PBPK model parameters (38) with comparison with observed data from literature studies, presented on semi-log plot. For each clinical study, 100 trials were simulated. The 5th and 95th percentiles of the simulated concentrations at each time-point for all virtual individuals were calculated (green shaded area). For studies that the observed data were reported as mean profiles, the mean concentration for all time-points were calculated for each trial, and subsequently the 5^th^ and 95^th^ percentiles of these 100 Trial Means were calculated (magenta shaded area). References and study design are listed in Table S4. C_plasma_ concentration of ganciclovir in plasma; HIV/CMV subjects seropositive for human immunodeficiency virus or cytomegalovirus; HV healthy volunteers.

Figure S10. Predicted pharmacokinetic parameters for ganciclovir, following intravenous administration of ganciclovir, using literature PBPK model parameters (38) with comparison with observed data. 100 trials of each clinical study was simulated with the mean (i.e., “Trial mean”) and standard deviation (i.e., “Trial StDev”) of each trial calculated; next the median and 5^th^ and 95^th^ percentiles of these 100 Trial Means ± StDev were calculated for each parameter and study. References and study design are listed in Table S4, observed data listed in Table S5. AUC_0-inf_ area under the ganciclovir plasma concentration-time curve extrapolated to infinity; CL_IV_ intravenous plasma clearance; CL_R_ renal excretion clearance; HIV/CMV subjects seropositive for human immunodeficiency virus or cytomegalovirus; HV healthy volunteers; t_1/2_ half-life of terminal phase of plasma concentration-time profile; V_d_ apparent volume of distribution; V_ss_ volume of distribution at steady-state.

Table S15 Prediction metrics for simulated gancicolivir pharmacokinetic parameters, following intravenous administration of ganciclovir, using literature PBPK model parameters (38) ^a^

|  | AFE | GMFE | RMSE |
| --- | --- | --- | --- |
| AUC_0-inf_ | 0.99 | 1.06 | 1.58 |
| CL_IV_ | 1.13 | 1.13 | 2.03 |
| t_1/2_ | 0.83 | 1.21 | 0.62 |
| V_d_ | 0.90 | 1.11 | 8.73 |
| V_ss_ | 1.11 | 1.11 | 5.69 |
| CL_R_ | 1.31 | 1.31 | 4.04 |

^a^ For each clinical study, 100 trials were simulated. For studies that reported parameters as mean values, the mean of each trial was calculated for the virtual subjects within the trial, and subsequently the median of these 100 Trial Means were calculated. References and study design are listed in Table S4, observed data listed in Table S5. AFE Average fold error (Equation 1 in main text); AUC_0-inf_ area under the ganciclovir plasma concentration-time curve extrapolated to infinity; CL_IV_ intravenous plasma clearance; CL_R_ renal excretion clearance; fe fraction of dose excreted in urine; GMFE Geometric fold error (Equation 2 in main text); HIV/CMV subjects seropositive for human immunodeficiency virus or cytomegalovirus; HV healthy volunteers; RMSE Root mean square error (Equation 3 in main text); t_1/2_ half-life of terminal phase of plasma concentration-time profile; V_d_ apparent volume of distribution; V_ss_ volume of distribution at steady-state.

# Refinement of ganciclovir PBPK model (elimination parameters)

Parameterisation of elements relating to elimination in the ganciclovir model was performed in two steps. While this step-wise/ iterative approach to model parameter identification within PBPK framework is recognised under best-practice concepts (131), a common limitation is that formal estimation of uncertainty around parameter estimates (e.g., standard error, %CV, confidence intervals) are not easily obtained (132). A post-hoc sensitivity analysis, along with uncertainty analysis, was therefore also performed to evaluate model reliability (Supplementary material, Section 6).

In the first step, an apparent intrinsic hepatic microsomal metabolic clearance (CL_int_; µL/min/mg microsomal protein) was estimated by back-calculation from CL_IV_ (corrected for CL_R_) using the well-stirred liver model, using the ‘Retrograde Reverse Translation Tool’ of the Simcyp Simulator (130). The assumed ganciclovir CL_IV_ and CL_R_ were 15.4 L/h (weighted mean in subjects with normal renal function, Table S5) and 13.0 L/h (weighted mean in subjects with normal renal function). Here the “Sim-Healthy Volunteers” population was used, with a representative trial design with age range 18 to 65 years, and proportion of females of 0.192, consistent with all subjects from which weighted mean CL_IV_ was derived (116-119). The estimated ‘Additional Clearance (Liver)’ (i.e., not attributed to any specific enzyme) CL_int_ was 0.49 µL/min/mg microsomal protein.

In the second step, the renal organic anion transporter (OAT)1 CL_int_ was optimised using a manual step-wise parameter search; here the median and 5^th^ - 95^th^ percentiles of CL_R_, fe and CL_IV_ 100 virtual individuals, resulting in an optimised transporter CL_int_ of 6 µL/min/million proximal tubule cells (Table S16).

Clinical pharmacokinetic studies with intravenous administration of ganciclovir in subjects with normal renal function were re-simulated with the revised ganciclovir PBPK model, with the results compared to the observed clinical data. As shown in Figure S11, Figure S12 and Figure S13, the simulated CL_R_ was no longer over-predicted (AFE = 1.0), and GMFE < 1.25 for all pharmacokinetic parameters (Table S17).

Table S16 Manual step-wise parameter search to optimise ganciclovir CL_int,OAT1,kidney_ parameter using PBPK simulations of 100

virtual subjects with normal renal function

| **Simulation #** | **1** | **2** | **3** | **4** | **5** | **Target from observed data** |
| --- | --- | --- | --- | --- | --- | --- |
| **CL_int,OAT1,kidney_ (µL/min/million PTC)** | **1** | **20** | **10** | **5** | **6** |  |
| **CL_R_ (L/h)** | | | | | | |
| **Median** | 8.4 | 22.6 | 16.1 | 12.3 | 13.1 | 13.0 ^a^  (12.2-13.8) |
| **5^th^ – 95^th^ percentile** | 6.3 – 13.6 | 12.5 – 41.4 | 9.7 – 29.3 | 8.0 – 20.5 | 8.4 – 22.4 |  |
|  |  |  |  |  |  |  |
| **fe** | | | | | | |
| **Median** | 0.83 | 0.94 | 0.91 | 0.88 | 0.89 | 0.88 ^b^  (0.85-0.91) |
| **5^th^ – 95^th^ percentile** | 0.72 – 0.91 | 0.84 – 0.97 | 0.80 – 0.96 | 0.76 – 0.95 | 0.77 – 0.95 |  |
|  |  |  |  |  |  |  |
| **CL_IV_ (L/h)** | | | | | | |
| **Median** | 10.1 | 24.1 | 17.5 | 14.3 | 15.2 | 15.4 ^c^  (14.3 - 16.2) |
| **5^th^ – 95^th^ percentile** | 8.0 – 15.1 | 14.6 – 43.6 | 11.6 – 31.5 | 9.6 – 22.6 | 9.9 – 24.6 |  |

^a^ Weighted mean of values reported by (116);

^b^ Weighted mean of fe calculated using values reported by from (116), fe = CL_R_/ CL_IV_;

^c^ Weighted mean of values reported by (116, 118, 119); CL_int,OAT1,kidney_ intrinsic clearance for OAT1 in kidney; CL­_IV_ plasma clearance after intravenous administration;

CL_R_ renal excretion clearance; fe fraction of dose excreted in urine; PBPK physiologically based pharmacokinetic; PTC proximal tubule cells

Figure S11 Predicted pharmacokinetic parameters for ganciclovir, following intravenous administration of ganciclovir, using refined PBPK model elimination parameters with comparison with observed data. 100 trials of each clinical study was simulated with the mean (i.e., “Trial mean”) and standard deviation (i.e., “Trial StDev”) of each trial calculated; next the median and 5^th^ and 95^th^ percentiles of these 100 Trial Means ± StDev were calculated for each parameter and study. References and study design are listed in Table S4, observed data listed in Table S5. AUC_0-inf_ area under the ganciclovir plasma concentration-time curve extrapolated to infinity; CL_IV_ intravenous plasma clearance; CL_R_ renal excretion clearance; fe fraction of dose excreted in urine; HIV/CMV subjects seropositive for human immunodeficiency virus or cytomegalovirus; HV healthy volunteers; t_1/2_ half-life of terminal phase of plasma concentration-time profile; V_d_ apparent volume of distribution; V_ss_ volume of distribution at steady-state.

Figure S12 Simulated gancicolivir plasma concentration-time profiles, following intravenous administration of ganciclovir, using refined PBPK model elimination parameters, with comparison with observed data from literature studies. For each clinical study, 100 trials were simulated. The 5th and 95th percentiles of the simulated concentrations at each time-point for all virtual individuals were calculated (green shaded area). For studies that the observed data were reported as mean profiles, the mean concentration for all time-points were calculated for each trial, and subsequently the 5^th^ and 95^th^ percentiles of these 100 Trial Means were calculated (magenta shaded area). References and study design are listed in Table S4. C_plasma_ concentration of ganciclovir in plasma; HIV/CMV subjects seropositive for human immunodeficiency virus or cytomegalovirus; HV healthy volunteers.

Figure S13 Simulated gancicolivir plasma concentration-time profiles, following intravenous administration of ganciclovir, using refined PBPK model elimination parameters, with comparison with observed data from literature studies, presented on semi-log plot. For each clinical study, 100 trials were simulated. The 5_th_ and 95_th_ percentiles of the simulated concentrations at each time-point for all virtual individuals were calculated (green shaded area). For studies that the observed data were reported as mean profiles, the mean concentration for all time-points were calculated for each trial, and subsequently the 5^th^ and 95^th^ percentiles of these 100 Trial Means were calculated (magenta shaded area). References and study design are listed in Table S4. C_plasma_ concentration of ganciclovir in plasma; HIV/CMV subjects seropositive for human immunodeficiency virus or cytomegalovirus; HV healthy volunteers.

Table S17 Prediction metrics for simulated gancicolivir pharmacokinetic parameters, following intravenous administration of ganciclovir, using refined PBPK model elimination parameters ^a^

|  | AFE | GMFE | RMSE |
| --- | --- | --- | --- |
| AUC_0-inf_ | 1.09 | 1.09 | 2.82 |
| CL_IV_ | 0.99 | 1.03 | 0.65 |
| t_1/2_ | 0.89 | 1.12 | 0.42 |
| V_d_ | 0.87 | 1.14 | 10.34 |
| V_ss_ | 1.11 | 1.11 | 5.69 |
| CL_R_ | 1.01 | 1.07 | 0.93 |

^a^ For each clinical study, 100 trials were simulated. For studies that reported parameters as mean values, the mean of each trial was calculated for the virtual subjects within the trial, and subsequently the median of these 100 Trial Means were calculated. References and study design are listed in Table S4, observed data listed in Table S5. AFE Average fold error (Equation 1 in main text); AUC_0-inf_ area under the ganciclovir plasma concentration-time curve extrapolated to infinity; CL_IV_ intravenous plasma clearance; CL_R_ renal excretion clearance; fe fraction of dose excreted in urine; GMFE Geometric fold error (Equation 2 in main text); HIV/CMV subjects seropositive for human immunodeficiency virus or cytomegalovirus; HV healthy volunteers; RMSE Root mean square error (Equation 3 in main text); t_1/2_ half-life of terminal phase of plasma concentration-time profile; V_d_ apparent volume of distribution; V_ss_ volume of distribution at steady-state.


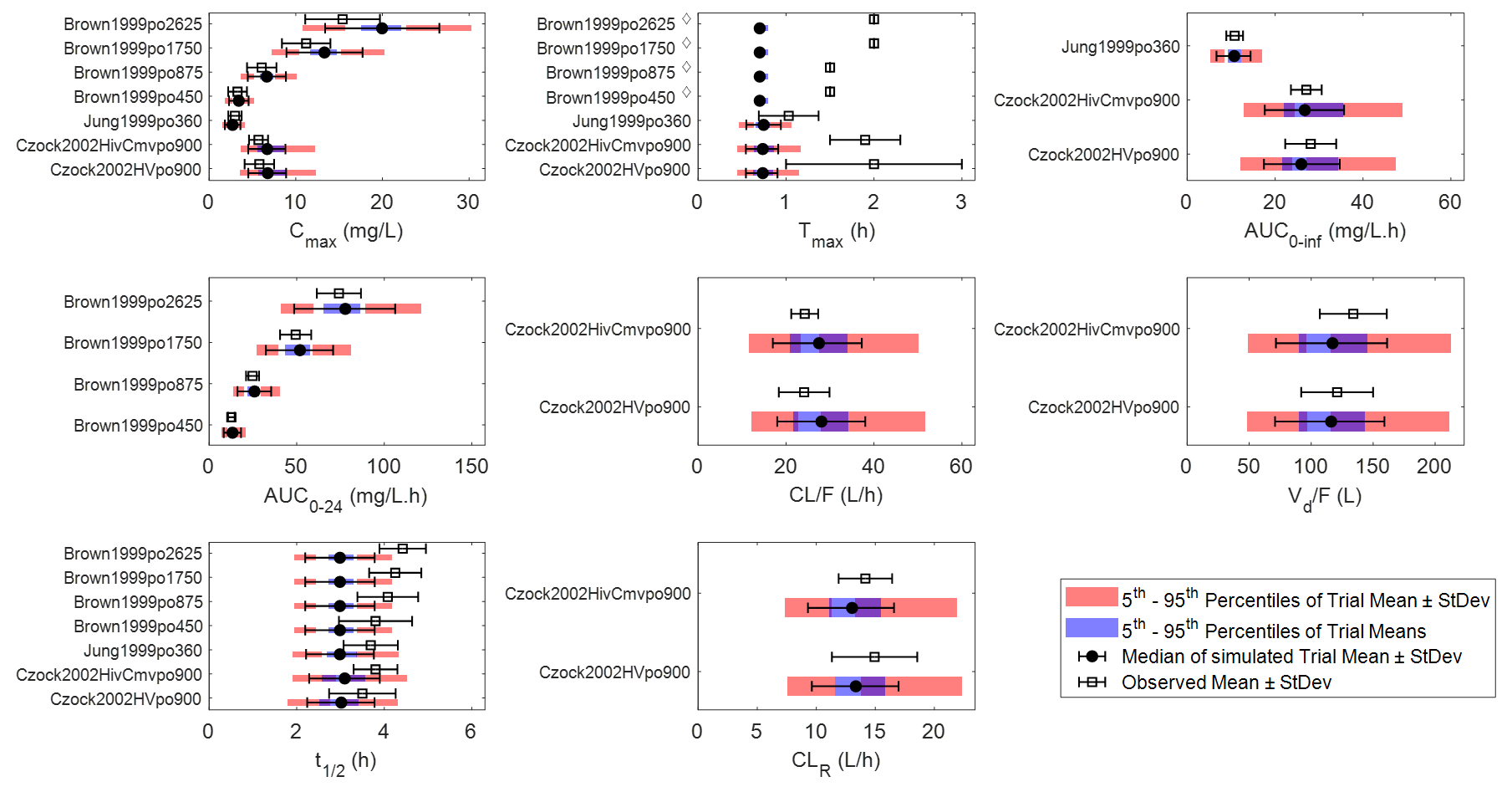


Figure S14 Comparison of predicted pharmacokinetic parameters for ganciclovir after oral administration of valganciclovir using refined PBPK model and observed data in subjects with normal renal function. Median and 5th - 95th percentile ranges of clinical trial means ± standard deviation (StDev) were calculated from 100 virtual trials per clinical study. ◊ Simulated and observed median for trials were calculated and reported, due to availability of data. Study designs and observed data are listed in Table S4 and Table S6, respectively. AUC_0-inf_ area under the ganciclovir plasma concentration-time curve extrapolated to infinity; AUC_0-24_ area under the ganciclovir plasma concentration-time curve up to 24h; C_max_ maximum observed concentration in plasma; CL/F oral plasma clearance; CL_R_ renal excretion clearance; t_1/2_ half-life of terminal phase of plasma concentration-time profile; T_max_ time at which the maximum concentration in plasma was observed; V_d_/F apparent volume of distribution.

Table S18 Assessment of PBPK predictions of ganciclovir pharmacokinetic parameters for specific clinical studies, following oral administration of valganciclovir, using refined PBPK model elimination parameters, assessed against 99.998% confidence interval around the geometric mean for observed data (133) ^a^

| Valganciclovir dose ^b^ (Reference) | C_max_ | T_max_ | AUC_0-inf_ | AUC_0-t_ | CL/F | Vd/f | t_1/2_ | CL_R_ |
| --- | --- | --- | --- | --- | --- | --- | --- | --- |
| 900 mg  (116, 117) | Yes (95%) | No (0%) | Yes (97%) | N/A | Yes (96%) | Yes (98%) | Yes (94%) | Yes (100%) |
| 900 mg  (116, 117) | Yes (81%) | No (0%) | Yes (82%) | N/A | Yes (69%) | Yes (86%) | No (46%) | Yes (94%) |
| 360 mg  (119) | Yes (97%) | No (49%) | Yes (96%) | N/A | N/A | N/A | No (30%) | N/A |
| 450 mg ^c^  (121) | Yes (100%) | N/A | N/A | Yes (90%) | N/A | N/A | No (27%) | N/A |
| 875 mg ^c^  (121) | Yes (100%) | N/A | N/A | Yes (91%) | N/A | N/A | No (1%) | N/A |
| 1750 mg ^c^  (121) | Yes (79%) | N/A | N/A | Yes (96%) | N/A | N/A | No (0%) | N/A |
| 2625 mg ^c^  (121) | Yes (51%) | N/A | N/A | Yes (96%) | N/A | N/A | No (0%) | N/A |
| Overall | 7/7 | 0/3 | 3/3 | 4/4 | 2/2 | 2/2 | 1/7 | 2/2 |

^a^ For each clinical study, 100 trials were simulated. For studies that reported parameters as mean values, the mean of each trial was calculated for the virtual subjects within the trial. “Yes” indicates that the median of these 100 Trial Means were within the 99.998% confidence interval around the geometric mean for observed data. Values in parentheses are the percentage of the 100 Trial Means that were within the 99.998% confidence interval around the geometric mean for observed data; ^b^ Study designs and observed data are listed in Table S5 and Table S7, respectively; ^c^ once daily dose over 3 days. AUC_0-inf_ area under the ganciclovir plasma concentration-time curve extrapolated to infinity; AUC_0-24_ area under the ganciclovir plasma concentration-time curve up to 24h; C_max_ maximum observed concentration in plasma; CL/F oral plasma clearance; CL_R_ renal excretion clearance; t_1/2_ half-life of terminal phase of plasma concentration-time profile; T_max_ time at which the maximum concentration in plasma was observed; V_d_/F apparent volume of distribution.

Table S19 Prediction metrics for simulated ganciclovir pharmacokinetic parameters, following oral administration of valganciclovir, using refined PBPK model elimination parameters ^a^

|  | AFE | GMFE | RMSE |
| --- | --- | --- | --- |
| C_max_ | 1.12 | 1.15 | 1.98 |
| T_max_ | 0.47 | 2.14 | 1.01 |
| AUC_0-inf_ | 0.97 | 1.03 | 1.26 |
| AUC_0-24_ | 1.05 | 1.05 | 2.27 |
| CL/F | 1.15 | 1.15 | 3.60 |
| V_d_/F | 0.92 | 1.09 | 12.37 |
| t_1/2_ | 0.77 | 1.30 | 0.98 |
| CL_R_ | 0.91 | 1.10 | 1.38 |

^a^ For each clinical study, 100 trials were simulated. For studies that reported parameters as mean values, the mean of each trial was calculated for the virtual subjects within the trial, and subsequently the median of these 100 Trial Means were calculated. Study designs and observed data are listed in Table S5 and Table S7, respectively. AFE Average fold error (Equation 1 in main text); AUC_0-inf_ area under the ganciclovir plasma concentration-time curve extrapolated to infinity; AUC_0-24_ area under the ganciclovir plasma concentration-time curve up to 24h; C_max_ maximum observed concentration in plasma; CL/F oral plasma clearance; CL_R_ renal excretion clearance; GMFE Geometric fold error (Equation 2 in main text); RMSE Root mean square error (Equation 3 in main text); t_1/2_ half-life of terminal phase of plasma concentration-time profile; T_max_ time at which the maximum concentration in plasma was observed; V_d_/F apparent volume of distribution.

# Simulation of ganciclovir systemic pharmacokinetics in subjects with normal and impaired renal function, using different population models for renal impairment


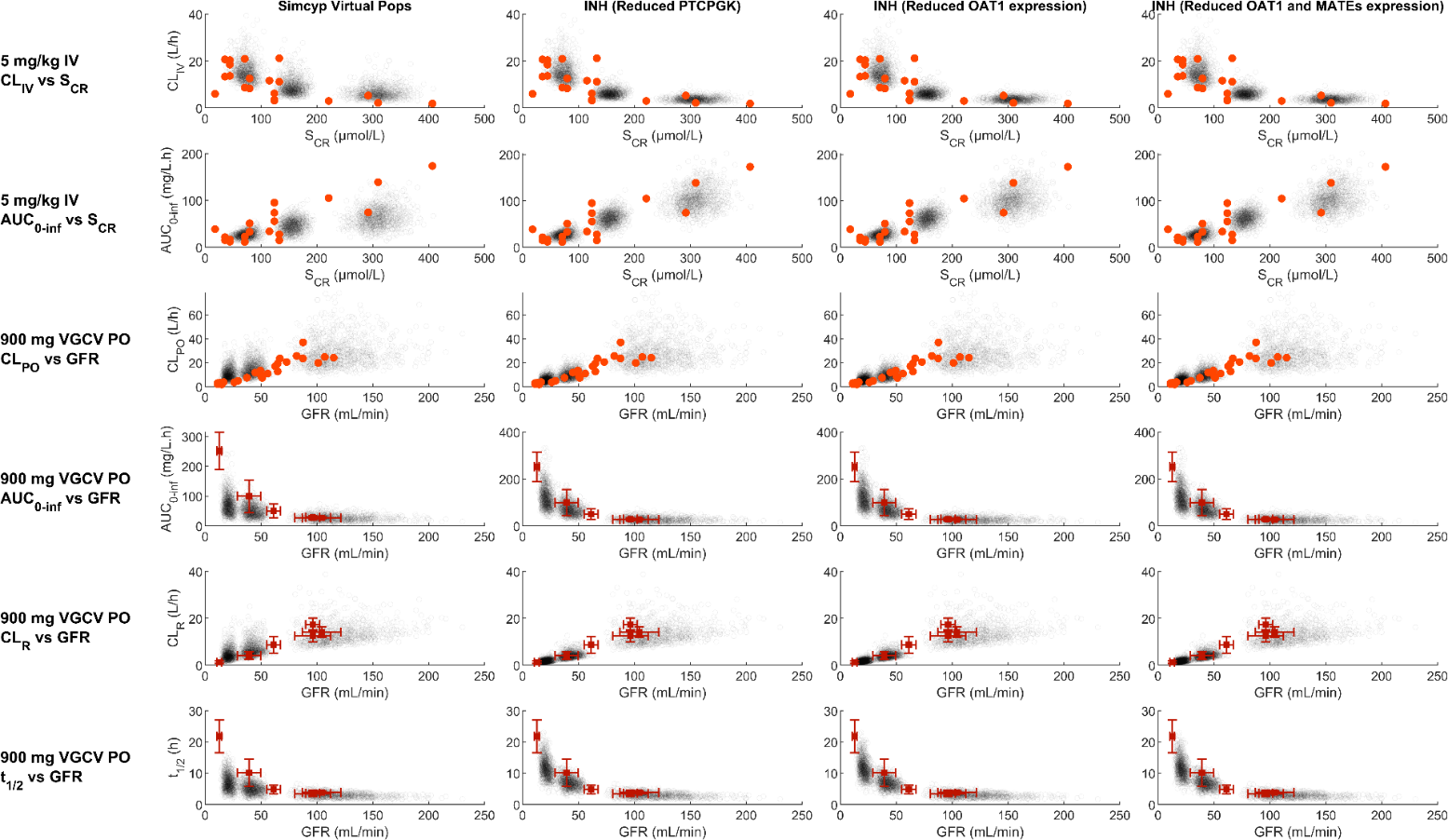


Figure S15 Simulated pharmacokinetic parameters and glomerular filtration rate (GFR) or serum creatinine concentration (S_CR­_), in subjects with normal and impaired renal function, using various renal impairment population models without (“Simcyp Virtual Pops”) and with (“INH …”) changes in systems parameters designed to mimic the effects on secretion in accordance with the intact nephron hypothesis (INH) (Table 1 in main text). Simulations (grey open circles) were performed with 1000 virtual subjects from each of the normal renal function (GFR > 90 mL/min), moderate renal impairment (30 mL/min < GFR < 60 mL/min) and severe renal impairment (GFR < 30mL/min) populations, following the overall design of specific clinical studies (Table S7). Observed clinical data presented are for individuals (bright red filled circles) or mean ± standard deviation (dark red filled squares and error bars). AUC_0-inf_ area under the ganciclovir plasma concentration-time curve extrapolated to infinity; CL_PO_ oral plasma clearance; CL_IV_ Clearance after intravenous administration; CL_R_ renal excretion clearance; GFR glomerular filtration rate; INH Intact nephron hypothesis; IV intravenous administration; MATE Multidrug and toxin extrusion; OAT1 Organic anion transporter 1; PO oral administration; PTCPGK Proximal tubule cellularity per gram kidney; t_1/2_ half-life of terminal phase of plasma concentration-time profile; VGCV valganciclovir.

# Simulated ganciclovir concentrations in lumen of nephron sub-regions

Initially, the median simulated ganciclovir concentrations in the lumen of each of nephron sub-region were compared, with a simulation of 100 virtual subjects from the ‘Healthy Volunteers’ population. The highest simulated ganciclovir tubular concentrations were in the medullary collecting duct (Figure S16).

Figure S16 Simulated ganciclovir concentrations in nephron tubules sub-regions (C_Tubule_) in the Healthy Volunteer virtual population, following single dose oral administration of 900 mg valganciclovir. Lines each represent median of all virtual subjects from 100 trials of the study design of (116, 117). PT1/2/3 Proximal tubule segment 1/2/3; HL Henle’s Loop; DT Distal Tubule; CortCD Cortical collecting duct; MeduCD Medullary collecting duct.

Figure S17 Distributions of selected systems parameters from a simulation of 1000 virtual subjects from each of the Healthy Volunteers (HV), North European Caucasian (NEurC) and Geriatric virtual populations. Virtual populations were not altered from those provided with the Simcyp simulator (v19r1).

Figure S18 Individual simulated maximum ganciclovir concentration in medullary collecting duct tubule (Medu-CD,Tubule) after single 900 mg oral dose of valganciclovir in various virtual populations with normal (‘Healthy’) or impaired (‘CKD’) renal function. Open circles represent values for 1000 virtual subjects from each population; grey shaded area represents range of reported aqueous solubility for water and physiological conditions (134). “Relative Kidney Organic Anion Transporter 1 (OAT1)” refers to the relative protein abundance of OAT1 transporter in kidney. CKD Chronic kidney disease; Filt. Flow Filtrate flow rate; GFR Glomerular filtration rate; Hct Haematorcit; HSA Plasma concentration of human serum albumin; PT1 Proximal tubule, segment1; PTCPGK proximal tubule cells per gram kidney; SCr serum creatinine concentration.


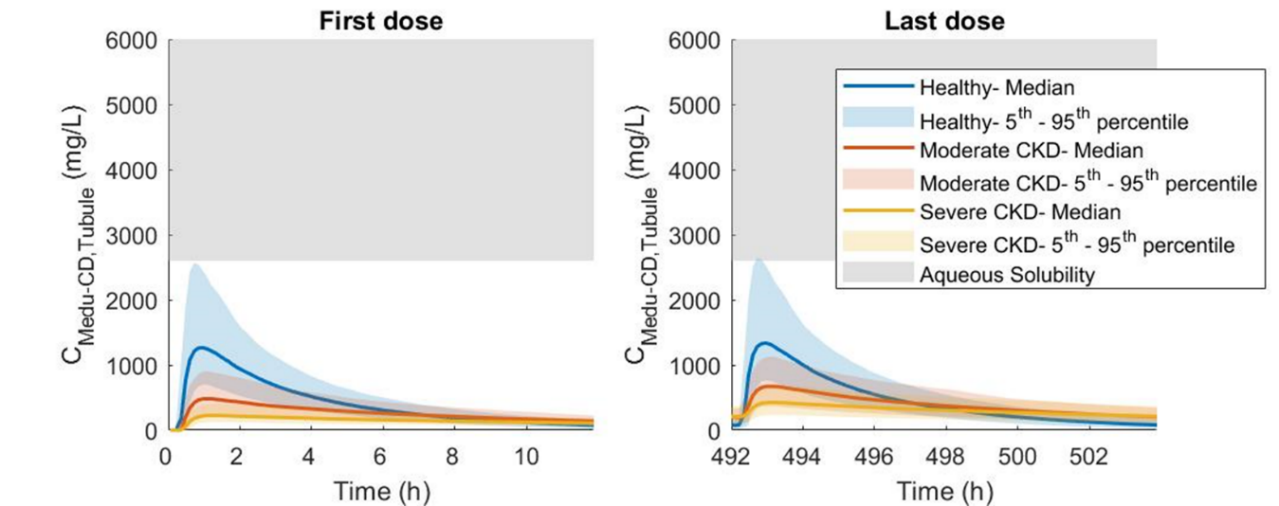


Figure S19 Simulated medullary collecting duct tubular concentration (C_Medu-CD,Tubule_) profiles of ganciclovir after first and last dose during 21 days of twice daily 900 mg oral dose of valganciclovir in virtual populations with normal or impaired renal function. The impaired renal function population was based on intact nephron hypothesis, implemented through reduction of PTCPGK in proportion to GFR. Median, and 5^th^ and 95 percentiles were calculated from 500 virtual subjects for each population. Grey shaded area represents range of reported aqueous solubility for water and physiological conditions (134). CKD Chronic kidney disease; PTCPGK Proximal tubule cells per gram kidney.

Figure S20 Simulated tubular concentration (C_Tubule_) profiles of ganciclovir in various regions of the nephron after last dose, following 21 days of twice daily 900 mg oral dose of valganciclovir. Simulations were performed in Healthy Volunteer virtual populations with wither normal (1 mL/min), moderate (0.5 mL/min) or low (0.1 mL/min) urine flow (UF) rates, respectively. Median, and 5^th^ and 95 percentiles were calculated from 200 virtual subjects for each urine flow rate scenario. Grey shaded area in each panel represents range of reported aqueous solubility for water and physiological conditions (134).

# References

1. Huang W, Czuba LC, Isoherranen N. Mechanistic PBPK Modeling of Urine pH Effect on Renal and Systemic Disposition of Methamphetamine and Amphetamine. J Pharmacol Exp Ther. 2020;373(3):488-501. doi: 10.1124/jpet.120.264994.

2. Matsuzaki T, Scotcher D, Darwich AS, Galetin A, Rostami-Hodjegan A. Towards Further Verification of Physiologically-Based Kidney Models: Predictability of the Effects of Urine-Flow and Urine-pH on Renal Clearance. J Pharmacol Exp Ther. 2019;368(2):157-68. doi: 10.1124/jpet.118.251413.

3. Scotcher D, Arya V, Yang X, Zhao P, Zhang L, Huang SM, Rostami-Hodjegan A, Galetin A. A Novel Physiologically Based Model of Creatinine Renal Disposition to Integrate Current Knowledge of Systems Parameters and Clinical Observations. CPT Pharmacometrics Syst Pharmacol. 2020;9(6):310-21. doi: 10.1002/psp4.12509.

4. Scotcher D, Arya V, Yang X, Zhao P, Zhang L, Huang SM, Rostami-Hodjegan A, Galetin A. Mechanistic Models as Framework for Understanding Biomarker Disposition: Prediction of Creatinine-Drug Interactions. CPT Pharmacometrics Syst Pharmacol. 2020;9(5):282-93. doi: 10.1002/psp4.12508.

5. Li Z, Litchfield J, Tess DA, Carlo AA, Eng H, Keefer C, Maurer TS. A Physiologically Based in Silico Tool to Assess the Risk of Drug-Related Crystalluria. J Med Chem. 2020;63(12):6489-98. doi: 10.1021/acs.jmedchem.9b01995.

6. Huang W, Isoherranen N. Development of a Dynamic Physiologically Based Mechanistic Kidney Model to Predict Renal Clearance. CPT Pharmacometrics Syst Pharmacol. 2018;7(9):593-602. doi: 10.1002/psp4.12321.

7. Emami Riedmaier A, Burt H, Abduljalil K, Neuhoff S. More Power to OATP1B1: An Evaluation of Sample Size in Pharmacogenetic Studies Using a Rosuvastatin PBPK Model for Intestinal, Hepatic, and Renal Transporter-Mediated Clearances. J Clin Pharmacol. 2016;56(Suppl 7):S132-42. doi: 10.1002/jcph.669.

8. Tang-Liu DD, Tozer TN, Riegelman S. Dependence of renal clearance on urine flow: a mathematical model and its application. J Pharm Sci. 1983;72(2):154-8. doi: 10.1002/jps.2600720215.

9. Tang-Liu DD, Tozer TN, Riegelman S. Urine flow-dependence of theophylline renal clearance in man. J Pharmacokinet Biopharm. 1982;10(4):351-64. doi: 10.1007/BF01065168.

10. Neuhoff S, Gaohua L, Burt H, Jamei M, Li L, Tucker GT, Rostami-Hodjegan A. Accounting for transporters in renal clearance: towards a mechanistic kidney model (Mech KiM). In: Sugiyama Y, Steffansen B, editors. Transporters in Drug Development. New York: Springer; 2013. p. 155-77.

11. Rhee SJ, Lee HA, Lee S, Kim E, Jeon I, Song IS, Yu KS. Physiologically Based Pharmacokinetic Modeling of Fimasartan, Amlodipine, and Hydrochlorothiazide for the Investigation of Drug-Drug Interaction Potentials. Pharm Res. 2018;35(12):236. doi: 10.1007/s11095-018-2511-5.

12. Yakovleva T, Sokolov V, Chu L, Tang W, Greasley PJ, Peilot Sjögren H, Johansson S, Peskov K, Helmlinger G, Boulton DW, Penland RC. Comparison of the urinary glucose excretion contributions of SGLT2 and SGLT1: A quantitative systems pharmacology analysis in healthy individuals and patients with type 2 diabetes treated with SGLT2 inhibitors. Diabetes Obes Metab. 2019;21(12):2684-93. doi: 10.1111/dom.13858.

13. Burt H, Neuhoff S, Almond L, Gaohua L, Harwood M, Jamei M, Rostami-Hodjegan A, Tucker G, Rowland-Yeo K. Metformin and cimetidine: Physiologically based pharmacokinetic modelling to investigate transporter mediated drug–drug interactions. Eur J Pharm Sci. 2016;88:70-82.

14. Posada MM, Bacon JA, Schneck KB, Tirona RG, Kim RB, Higgins JW, Pak YA, Hall SD, Hillgren KM. Prediction of Renal Transporter Mediated Drug-Drug Interactions for Pemetrexed Using Physiologically Based Pharmacokinetic Modeling. Drug Metab Dispos. 2015;43(3):325-34. doi: 10.1124/dmd.114.059618.

15. Ball K, Jamier T, Parmentier Y, Denizot C, Mallier A, Chenel M. Prediction of renal transporter-mediated drug-drug interactions for a drug which is an OAT substrate and inhibitor using PBPK modelling. Eur J Pharm Sci. 2017;106:122-32. doi: 10.1016/j.ejps.2017.05.055.

16. Mori K, Saito R, Nakamaru Y, Shimizu M, Yamazaki H. Physiologically based pharmacokinetic-pharmacodynamic modeling to predict concentrations and actions of sodium-dependent glucose transporter 2 inhibitor canagliflozin in human intestines and renal tubules. Biopharm Drug Dispos. 2016;37(8):491-506. doi: 10.1002/bdd.2040.

17. Posada MM, Cannady EA, Payne CD, Zhang X, Bacon JA, Pak YA, Higgins JW, Shahri N, Hall SD, Hillgren KM. Prediction of Transporter-Mediated Drug-Drug Interactions for Baricitinib. Clin Transl Sci. 2017;10(6):509-19. doi: 10.1111/cts.12486.

18. Nishiyama K, Toshimoto K, Lee W, Ishiguro N, Bister B, Sugiyama Y. Physiologically-Based Pharmacokinetic Modeling Analysis for Quantitative Prediction of Renal Transporter-Mediated Interactions Between Metformin and Cimetidine. CPT Pharmacometrics Syst Pharmacol. 2019;8(6):396-406. doi: 10.1002/psp4.12398.

19. Worley RR, Yang X, Fisher J. Physiologically based pharmacokinetic modeling of human exposure to perfluorooctanoic acid suggests historical non drinking-water exposures are important for predicting current serum concentrations. Toxicol Appl Pharmacol. 2017;330:9-21. doi: 10.1016/j.taap.2017.07.001.

20. Chou WC, Lin Z. Bayesian evaluation of a physiologically based pharmacokinetic (PBPK) model for perfluorooctane sulfonate (PFOS) to characterize the interspecies uncertainty between mice, rats, monkeys, and humans: Development and performance verification. Environ Int. 2019;129:408-22. doi: 10.1016/j.envint.2019.03.058.

21. Ogungbenro K, Aarons L. Physiologically based pharmacokinetic modelling of methotrexate and 6-mercaptopurine in adults and children. Part 2: 6-mercaptopurine and its interaction with methotrexate. J Pharmacokinet Pharmacodyn. 2014;41(2):173-85. doi: 10.1007/s10928-014-9355-3.

22. Ogungbenro K, Aarons L. Physiologically based pharmacokinetic modelling of methotrexate and 6-mercaptopurine in adults and children. Part 1: methotrexate. J Pharmacokinet Pharmacodyn. 2014;41(2):159-71.

23. Liu SN, Desta Z, Gufford BT. Probenecid-Boosted Tenofovir: A Physiologically-Based Pharmacokinetic Model-Informed Strategy for On-Demand HIV Preexposure Prophylaxis. CPT Pharmacometrics Syst Pharmacol. 2020;9(1):40-7. doi: 10.1002/psp4.12481.

24. De Sousa Mendes M, Hirt D, Urien S, Valade E, Bouazza N, Foissac F, Blanche S, Treluyer JM, Benaboud S. Physiologically-based pharmacokinetic modeling of renally excreted antiretroviral drugs in pregnant women. Br J Clin Pharmacol. 2015;80(5):1031-41. doi: 10.1111/bcp.12685.

25. Ali I, Guidone D, Nicolazzo JA, Brouwer KLR. Impact of reduced P-glycoprotein function on digoxin concentrations in patients with dementia. Br J Clin Pharmacol. 2019;85(10):2351-9. doi: 10.1111/bcp.14049.

26. Brightman FA, Leahy DE, Searle GE, Thomas S. Application of a generic physiologically based pharmacokinetic model to the estimation of xenobiotic levels in human plasma. Drug Metab Dispos. 2006;34(1):94-101. doi: 10.1124/dmd.105.004838.

27. Balazki P, Schaller S, Eissing T, Lehr T. A Quantitative Systems Pharmacology Kidney Model of Diabetes Associated Renal Hyperfiltration and the Effects of SGLT Inhibitors. CPT Pharmacometrics Syst Pharmacol. 2018;7(12):788-97. doi: 10.1002/psp4.12359.

28. Demin O, Jr., Yakovleva T, Kolobkov D, Demin O. Analysis of the efficacy of SGLT2 inhibitors using semi-mechanistic model. Front Pharmacol. 2014;5:218. doi: 10.3389/fphar.2014.00218.

29. Britz H, Hanke N, Taub ME, Wang T, Prasad B, Fernandez É, Stopfer P, Nock V, Lehr T. Physiologically Based Pharmacokinetic Models of Probenecid and Furosemide to Predict Transporter Mediated Drug-Drug Interactions. Pharm Res. 2020;37(12):250. doi: 10.1007/s11095-020-02964-z.

30. Mori-Anai K, Tashima Y, Nakada T, Nakamaru Y, Takahata T, Saito R. Mechanistic evaluation of the effect of sodium-dependent glucose transporter 2 inhibitors on delayed glucose absorption in patients with type 2 diabetes mellitus using a quantitative systems pharmacology model of human systemic glucose dynamics. Biopharm Drug Dispos. 2020;41(8-9):352-66. doi: 10.1002/bdd.2253.

31. Chapa R, Li CY, Basit A, Thakur A, Ladumor MK, Sharma S, Singh S, Selen A, Prasad B. Contribution of Uptake and Efflux Transporters to Oral Pharmacokinetics of Furosemide. ACS Omega. 2020;5(51):32939-50. doi: 10.1021/acsomega.0c03930.

32. Zake DM, Kurlovics J, Zaharenko L, Komasilovs V, Klovins J, Stalidzans E. Physiologically based metformin pharmacokinetics model of mice and scale-up to humans for the estimation of concentrations in various tissues. PLoS ONE. 2021;16(4):e0249594. doi: 10.1371/journal.pone.0249594.

33. Chou WC, Lin Z. Development of a Gestational and Lactational Physiologically Based Pharmacokinetic (PBPK) Model for Perfluorooctane Sulfonate (PFOS) in Rats and Humans and Its Implications in the Derivation of Health-Based Toxicity Values. Environ Health Perspect. 2021;129(3):37004. doi: 10.1289/ehp7671.

34. Cheong EJY, Teo DWX, Chua DXY, Chan ECY. Systematic Development and Verification of a Physiologically Based Pharmacokinetic Model of Rivaroxaban. Drug Metab Dispos. 2019;47(11):1291-306. doi: 10.1124/dmd.119.086918.

35. Hsu V, de LT Vieira M, Zhao P, Zhang L, Zheng JH, Nordmark A, Berglund EG, Giacomini KM, Huang S-M. Towards quantitation of the effects of renal impairment and probenecid inhibition on kidney uptake and efflux transporters, using physiologically based pharmacokinetic modelling and simulations. Clin Pharmacokinet. 2014;53(3):283-93. doi: 10.1007/s40262-013-0117-y.

36. Rhee SJ, Chung H, Yi S, Yu KS, Chung JY. Physiologically Based Pharmacokinetic Modelling and Prediction of Metformin Pharmacokinetics in Renal/Hepatic-Impaired Young Adults and Elderly Populations. Eur J Drug Metab Pharmacokinet. 2017;42(6):973-80. doi: 10.1007/s13318-017-0418-x.

37. Scotcher D, Jones CR, Galetin A, Rostami-Hodjegan A. Delineating the Role of Various Factors in Renal Disposition of Digoxin through Application of Physiologically Based Kidney Model to Renal Impairment Populations. J Pharmacol Exp Ther. 2017;360(3):484-95. doi: 10.1124/jpet.116.237438.

38. Hsueh CH, Hsu V, Zhao P, Zhang L, Giacomini KM, Huang SM. PBPK Modeling of the Effect of Reduced Kidney Function on the Pharmacokinetics of Drugs Excreted Renally by Organic Anion Transporters. Clin Pharmacol Ther. 2018;103(3):485-92. doi: 10.1002/cpt.750.

39. Follman KE, Morris ME. Prediction of the Effects of Renal Impairment on Clearance for Organic Cation Drugs that Undergo Renal Secretion: A Simulation-Based Study. Drug Metab Dispos. 2018;46(5):758-69. doi: 10.1124/dmd.117.079558.

40. Fuhr LM, Hanke N, Meibohm B, Lehr T. Effective Removal of Dabigatran by Idarucizumab or Hemodialysis: A Physiologically Based Pharmacokinetic Modeling Analysis. Clin Pharmacokinet. 2020;59(6):809-25. doi: 10.1007/s40262-019-00857-y.

41. Huang W, Isoherranen N. Novel Mechanistic PBPK Model to Predict Renal Clearance in Varying Stages of CKD by Incorporating Tubular Adaptation and Dynamic Passive Reabsorption. CPT Pharmacometrics Syst Pharmacol. 2020. doi: 10.1002/psp4.12553.

42. Bergman A, Bi YA, Mathialagan S, Litchfield J, Kazierad DJ, Pfefferkorn JA, Varma MVS. Effect of Hepatic Organic Anion-Transporting Polypeptide 1B Inhibition and Chronic Kidney Disease on the Pharmacokinetics of a Liver-Targeted Glucokinase Activator: A Model-Based Evaluation. Clin Pharmacol Ther. 2019;106(4):792-802. doi: 10.1002/cpt.1419.

43. Takita H, Scotcher D, Chinnadurai R, Kalra PA, Galetin A. Physiologically-Based Pharmacokinetic Modelling of Creatinine-Drug Interactions in the Chronic Kidney Disease Population. CPT Pharmacometrics Syst Pharmacol. 2020;9(12):695-706. doi: 10.1002/psp4.12566.

44. Li J, Kim S, Sha X, Wiegand R, Wu J, LoRusso P. Complex disease–, gene–, and drug–drug interactions: impacts of renal function, CYP2D6 phenotype, and OCT2 activity on veliparib pharmacokinetics. Clin Cancer Res. 2014;20(15):3931-44.

45. Huang W, Nakano M, Sager J, Ragueneau-Majlessi I, Isoherranen N. Physiologically Based Pharmacokinetic Model of the CYP2D6 Probe Atomoxetine: Extrapolation to Special Populations and Drug-Drug Interactions. Drug Metab Dispos. 2017;45(11):1156-65. doi: 10.1124/dmd.117.076455.

46. Ghoneim AM, Mansour SM. The Effect of Liver and Kidney Disease on the Pharmacokinetics of Clozapine and Sildenafil: A Physiologically Based Pharmacokinetic Modeling. Drug Des Devel Ther. 2020;14:1469-79. doi: 10.2147/dddt.S246229.

47. Takubo H, Taniguchi T, Iwanaga K, Nomura Y. Evaluation of the changes in exposure to thiol compounds in chronic kidney disease patients using the PBPK model. Xenobiotica. 2020:1-9. doi: 10.1080/00498254.2020.1805816.

48. Sayama H, Takubo H, Komura H, Kogayu M, Iwaki M. Application of a physiologically based pharmacokinetic model informed by a top-down approach for the prediction of pharmacokinetics in chronic kidney disease patients. AAPS J. 2014;16(5):1018-28.

49. You X, Wu W, Xu J, Jiao Z, Ke M, Huang P, Lin C. Development of a Physiologically Based Pharmacokinetic Model for Prediction of Pramipexole Pharmacokinetics in Parkinson's Disease Patients With Renal Impairment. J Clin Pharmacol. 2020;60(8):999-1010. doi: 10.1002/jcph.1593.

50. Ye L, Ke M, You X, Huang P, Lin C. A Physiologically Based Pharmacokinetic Model of Ertapenem in Pediatric Patients With Renal Impairment. J Pharm Sci. 2020;109(9):2909-18. doi: 10.1016/j.xphs.2020.06.010.

51. Yee KL, Li M, Cabalu T, Sahasrabudhe V, Lin J, Zhao P, Jadhav P. Evaluation of Model-Based Prediction of Pharmacokinetics in the Renal Impairment Population. J Clin Pharmacol. 2018;58(3):364-76. doi: 10.1002/jcph.1022.

52. Zhou L, Tong X, Sharma P, Xu H, Al-Huniti N, Zhou D. Physiologically based pharmacokinetic modelling to predict exposure differences in healthy volunteers and subjects with renal impairment: Ceftazidime case study. Basic Clin Pharmacol Toxicol. 2019;125(2):100-7. doi: 10.1111/bcpt.13209.

53. Tan ML, Zhao P, Zhang L, Ho YF, Varma MVS, Neuhoff S, Nolin TD, Galetin A, Huang SM. Use of Physiologically Based Pharmacokinetic Modeling to Evaluate the Effect of Chronic Kidney Disease on the Disposition of Hepatic CYP2C8 and OATP1B Drug Substrates. Clin Pharmacol Ther. 2019;105(3):719-29. doi: 10.1002/cpt.1205.

54. Follman KE, Morris ME. Simulation-Based Analysis of the Impact of Renal Impairment on the Pharmacokinetics of Highly Metabolized Compounds. Pharmaceutics. 2019;11(3). doi: 10.3390/pharmaceutics11030105.

55. Almukainzi M, Gabr R, Abdelhamid G, Löbenberg R. Mechanistic understanding of the effect of renal impairment on metformin oral absorption using computer simulations. Journal of Pharmaceutical Investigation. 2017;47(2):151-61. doi: 10.1007/s40005-017-0307-y.

56. Hanke N, Türk D, Selzer D, Ishiguro N, Ebner T, Wiebe S, Müller F, Stopfer P, Nock V, Lehr T. A Comprehensive Whole-Body Physiologically Based Pharmacokinetic Drug-Drug-Gene Interaction Model of Metformin and Cimetidine in Healthy Adults and Renally Impaired Individuals. Clin Pharmacokinet. 2020. doi: 10.1007/s40262-020-00896-w.

57. Doki K, Neuhoff S, Rostami-Hodjegan A, Homma M. Assessing Potential Drug-Drug Interactions Between Dabigatran Etexilate and a P-Glycoprotein Inhibitor in Renal Impairment Populations Using Physiologically Based Pharmacokinetic Modeling. CPT Pharmacometrics Syst Pharmacol. 2019;8(2):118-26. doi: 10.1002/psp4.12382.

58. Li GF, Wang K, Chen R, Zhao HR, Yang J, Zheng QS. Simulation of the pharmacokinetics of bisoprolol in healthy adults and patients with impaired renal function using whole-body physiologically based pharmacokinetic modeling. Acta Pharmacol Sin. 2012;33(11):1359-71. doi: 10.1038/aps.2012.103.

59. Zhao P, Vieira MdL, Grillo JA, Song P, Wu TC, Zheng JH, Arya V, Berglund EG, Atkinson AJ, Sugiyama Y. Evaluation of exposure change of nonrenally eliminated drugs in patients with chronic kidney disease using physiologically based pharmacokinetic modeling and simulation. J Clin Pharmacol. 2012;52(S1):91S-108S.

60. Tortorici MA, Cutler DL, Hazra A, Nolin TD, Rowland‐Yeo K, Venkatakrishnan K. Emerging areas of research in the assessment of pharmacokinetics in patients with chronic kidney disease. J Clin Pharmacol. 2015;55(3):241-50.

61. Fujita K, Masuo Y, Okumura H, Watanabe Y, Suzuki H, Sunakawa Y, Shimada K, Kawara K, Akiyama Y, Kitamura M, Kunishima M, Sasaki Y, Kato Y. Increased Plasma Concentrations of Unbound SN-38, the Active Metabolite of Irinotecan, in Cancer Patients with Severe Renal Failure. Pharm Res. 2016;33(2):269-82. doi: 10.1007/s11095-015-1785-0.

62. Rowland Yeo K, Aarabi M, Jamei M, Rostami-Hodjegan A. Modeling and predicting drug pharmacokinetics in patients with renal impairment. Expert Rev Clin Pharmacol. 2011;4(2):261-74.

63. Lu C, Suri A, Shyu WC, Prakash S. Assessment of cytochrome P450-mediated drug-drug interaction potential of orteronel and exposure changes in patients with renal impairment using physiologically based pharmacokinetic modeling and simulation. Biopharm Drug Dispos. 2014;35(9):543-52. doi: 10.1002/bdd.1919.

64. Grillo JA, Zhao P, Bullock J, Booth BP, Lu M, Robie‐Suh K, Berglund EG, Pang KS, Rahman A, Zhang L. Utility of a physiologically–based pharmacokinetic (PBPK) modeling approach to quantitatively predict a complex drug–drug–disease interaction scenario for rivaroxaban during the drug review process: implications for clinical practice. Biopharm Drug Dispos. 2012;33(2):99-110.

65. Snoeys J, Beumont M, Monshouwer M, Ouwerkerk-Mahadevan S. Elucidating the Plasma and Liver Pharmacokinetics of Simeprevir in Special Populations Using Physiologically Based Pharmacokinetic Modelling. Clin Pharmacokinet. 2017;56(7):781-92. doi: 10.1007/s40262-016-0476-2.

66. Moj D, Maas H, Schaeftlein A, Hanke N, Gómez-Mantilla JD, Lehr T. A Comprehensive Whole-Body Physiologically Based Pharmacokinetic Model of Dabigatran Etexilate, Dabigatran and Dabigatran Glucuronide in Healthy Adults and Renally Impaired Patients. Clin Pharmacokinet. 2019;58(12):1577-93. doi: 10.1007/s40262-019-00776-y.

67. Balbas-Martinez V, Michelet R, Edginton AN, Meesters K, Trocóniz IF, Vermeulen A. Physiologically-Based Pharmacokinetic model for Ciprofloxacin in children with complicated Urinary Tract Infection. Eur J Pharm Sci. 2019;128:171-9. doi: 10.1016/j.ejps.2018.11.033.

68. Konishi K, Minematsu T, Nagasaka Y, Tabata K. Application of a physiologically based pharmacokinetic model for the prediction of mirabegron plasma concentrations in a population with severe renal impairment. Biopharm Drug Dispos. 2019;40(5-6):176-87. doi: 10.1002/bdd.2181.

69. Xu R, Ge W, Jiang Q. Application of physiologically based pharmacokinetic modeling to the prediction of drug-drug and drug-disease interactions for rivaroxaban. Eur J Clin Pharmacol. 2018;74(6):755-65. doi: 10.1007/s00228-018-2430-8.

70. Higashimori M, Ishikawa K, Gillen M, Zhou D. Physiologically Based Pharmacokinetic Modelling of Glycopyrronium in Patients With Renal Impairment. J Pharm Sci. 2020. doi: 10.1016/j.xphs.2020.03.014.

71. Yoon S, Yi S, Rhee SJ, Lee HA, Kim Y, Yu KS, Chung JY. Development of a physiologically-based pharmacokinetic model for cyclosporine in Asian children with renal impairment. Transl Clin Pharmacol. 2019;27(3):107-14. doi: 10.12793/tcp.2019.27.3.107.

72. Ogawa SI, Shimizu M, Yamazaki H. Plasma concentrations of pemafibrate with co-administered drugs predicted by physiologically based pharmacokinetic modeling in virtual populations with renal/hepatic impairment. Xenobiotica. 2020;50(9):1023-31. doi: 10.1080/00498254.2019.1709133.

73. Nakamaru Y, Emoto C, Shimizu M, Yamazaki H. Human pharmacokinetic profiling of the dipeptidyl peptidase-IV inhibitor teneligliptin using physiologically based pharmacokinetic modeling. Biopharm Drug Dispos. 2015;36(3):148-62. doi: 10.1002/bdd.1928.

74. Ono C, Hsyu PH, Abbas R, Loi CM, Yamazaki S. Application of Physiologically Based Pharmacokinetic Modeling to the Understanding of Bosutinib Pharmacokinetics: Prediction of Drug-Drug and Drug-Disease Interactions. Drug Metab Dispos. 2017;45(4):390-8. doi: 10.1124/dmd.116.074450.

75. Emoto C, Johnson TN, McPhail BT, Vinks AA, Fukuda T. Using a Vancomycin PBPK Model in Special Populations to Elucidate Case-Based Clinical PK Observations. CPT Pharmacometrics Syst Pharmacol. 2018;7(4):237-50. doi: 10.1002/psp4.12279.

76. Ogawa SI, Shimizu M, Yamazaki H. Modelled plasma concentrations of pemafibrate with co-administered typical cytochrome P450 inhibitors clopidogrel, fluconazole or clarithromycin predicted by physiologically based pharmacokinetic modelling in virtual populations. Xenobiotica. 2020:1-10. doi: 10.1080/00498254.2020.1793030.

77. Tan W, Yamazaki S, Johnson TR, Wang R, O'Gorman MT, Kirkovsky L, Boutros T, Brega NM, Bello A. Effects of Renal Function on Crizotinib Pharmacokinetics: Dose Recommendations for Patients with ALK-Positive Non-Small Cell Lung Cancer. Clin Drug Investig. 2017;37(4):363-73. doi: 10.1007/s40261-016-0490-z.

78. Spanakis M, Marias K. In silico evaluation of gadofosveset pharmacokinetics in different population groups using the Simcyp® simulator platform. In Silico Pharmacol. 2014;2(1):2. doi: 10.1186/s40203-014-0002-x.

79. Spanakis M, Kontopodis E, Van Cauter S, Sakkalis V, Marias K. Assessment of DCE-MRI parameters for brain tumors through implementation of physiologically-based pharmacokinetic model approaches for Gd-DOTA. J Pharmacokinet Pharmacodyn. 2016;43(5):529-47. doi: 10.1007/s10928-016-9493-x.

80. Saeheng T, Na-Bangchang K, Siccardi M, Rajoli RKR, Karbwang J. Physiologically-Based Pharmacokinetic Modeling for Optimal Dosage Prediction of Quinine Coadministered With Ritonavir-Boosted Lopinavir. Clin Pharmacol Ther. 2020;107(5):1209-20. doi: 10.1002/cpt.1721.

81. Tse S, Dowty ME, Menon S, Gupta P, Krishnaswami S. Application of Physiologically Based Pharmacokinetic Modeling to Predict Drug Exposure and Support Dosing Recommendations for Potential Drug-Drug Interactions or in Special Populations: An Example Using Tofacitinib. J Clin Pharmacol. 2020. doi: 10.1002/jcph.1679.

82. Rowland Yeo K, Zhang M, Pan X, Ban Ke A, Jones HM, Wesche D, Almond LM. Impact of Disease on Plasma and Lung Exposure of Chloroquine, Hydroxychloroquine and Azithromycin: Application of PBPK Modeling. Clin Pharmacol Ther. 2020. doi: 10.1002/cpt.1955.

83. Fischetti B, Shah K, Taft DR, Berkowitz L, Bakshi A, Cha A. Real-World Experience With Higher-Than-Recommended Doses of Lamivudine in Patients With Varying Degrees of Renal Impairment. Open Forum Infect Dis. 2018;5(10):ofy225. doi: 10.1093/ofid/ofy225.

84. Ismail M, Lee VH, Chow CR, Rubino CM. Minimal Physiologically Based Pharmacokinetic and Drug-Drug-Disease Interaction Model of Rivaroxaban and Verapamil in Healthy and Renally Impaired Subjects. J Clin Pharmacol. 2018;58(4):541-8. doi: 10.1002/jcph.1044.

85. Polasek TM, Tucker GT, Sorich MJ, Wiese MD, Mohan T, Rostami-Hodjegan A, Korprasertthaworn P, Perera V, Rowland A. Prediction of olanzapine exposure in individual patients using physiologically based pharmacokinetic modelling and simulation. Br J Clin Pharmacol. 2018;84(3):462-76. doi: 10.1111/bcp.13480.

86. Rowland A, van Dyk M, Hopkins AM, Mounzer R, Polasek TM, Rostami-Hodjegan A, Sorich MJ. Physiologically Based Pharmacokinetic Modeling to Identify Physiological and Molecular Characteristics Driving Variability in Drug Exposure. Clin Pharmacol Ther. 2018;104(6):1219-28. doi: 10.1002/cpt.1076.

87. van Dyk M, Rowland A. Physiologically-based pharmacokinetic modeling as an approach to evaluate the effect of covariates and drug-drug interactions on variability in epidermal growth factor receptor kinase inhibitor exposure. Translational Cancer Research. 2017;6:S1600-S12.

88. Hossain M, Tiffany C, Raychaudhuri A, Nguyen D, Tai G, Alcorn H, Jr., Preston RA, Marbury T, Dumont E. Pharmacokinetics of Gepotidacin in Renal Impairment. Clin Pharmacol Drug Dev. 2020;9(5):560-72. doi: 10.1002/cpdd.807.

89. Malik PRV, Yeung CHT, Ismaeil S, Advani U, Djie S, Edginton AN. A Physiological Approach to Pharmacokinetics in Chronic Kidney Disease. J Clin Pharmacol. 2020;60 Suppl 1:S52-s62. doi: 10.1002/jcph.1713.

90. Lingineni K, Farhan N, Kim S, Gordon LA, Kumar P, Penzak S, Hadigan C, George JM, Brown JD, Schmidt S. Quantitative Benefit-Risk Assessment of P-gp Mediated Drug-Drug Interactions (DDIs) of Dabigatran Co-Administered with Pharmacokinetic Enhancers in Patients with Renal Impairment. Clin Pharmacol Ther. 2020. doi: 10.1002/cpt.2087.

91. Yu Y, Hoffman J, Plotka A, O'Gorman M, Shi H, Wang D. Palbociclib (PD-0332991) pharmacokinetics in subjects with impaired renal function. Cancer Chemother Pharmacol. 2020;86(6):701-10. doi: 10.1007/s00280-020-04163-4.

92. Heimbach T, Chen Y, Chen J, Dixit V, Parrott N, Peters SA, Poggesi I, Sharma P, Snoeys J, Shebley M, Tai G, Tse S, Upreti VV, Wang YH, Tsai A, Xia B, Zheng M, Zhu AZX, Hall S. Physiologically-Based Pharmacokinetic Modeling in Renal and Hepatic Impairment Populations: A Pharmaceutical Industry Perspective. Clin Pharmacol Ther. 2020. doi: 10.1002/cpt.2125.

93. Willmann S, Coboeken K, Kapsa S, Thelen K, Mundhenke M, Fischer K, Hügl B, Mück W. Applications of Physiologically Based Pharmacokinetic Modeling of Rivaroxaban-Renal and Hepatic Impairment and Drug-Drug Interaction Potential. J Clin Pharmacol. 2021;61(5):656-65. doi: 10.1002/jcph.1784.

94. Cui C, Li X, Liang H, Hou Z, Tu S, Dong Z, Yao X, Zhang M, Zhang X, Li H, Zuo X, Liu D. Physiologically based pharmacokinetic model of renally cleared antibacterial drugs in Chinese renal impairment patients. Biopharm Drug Dispos. 2021;42(1):24-34. doi: 10.1002/bdd.2258.

95. Wu W, Ke M, Ye L, Lin C. Application of physiologically based pharmacokinetic modeling to predict the pharmacokinetics of telavancin in obesity with renal impairment. Eur J Clin Pharmacol. 2021:1-10. doi: 10.1007/s00228-020-03072-y.

96. Zhou J, You X, Ke M, Ye L, Wu W, Huang P, Lin C. Dosage Adjustment for Ceftazidime in Pediatric Patients With Renal Impairment Using Physiologically Based Pharmacokinetic Modeling. J Pharm Sci. 2021;110(4):1853-62. doi: 10.1016/j.xphs.2021.02.001.

97. Rasool MF, Ali S, Khalid S, Khalid R, Majeed A, Imran I, Saeed H, Usman M, Ali M, Alali AS, AlAsmari AF, Ali N, Asiri AM, Alasmari F, Alqahtani F. Development and evaluation of physiologically based pharmacokinetic drug-disease models for predicting captopril pharmacokinetics in chronic diseases. Sci Rep. 2021;11(1):8589. doi: 10.1038/s41598-021-88154-2.

98. Emoto C, Johnson TN, Yamada T, Yamazaki H, Fukuda T. Teicoplanin physiologically based pharmacokinetic modeling offers a quantitative assessment of a theoretical influence of serum albumin and renal function on its disposition. Eur J Clin Pharmacol. 2021. doi: 10.1007/s00228-021-03098-w.

99. Zhang M, Yao X, Hou Z, Guo X, Tu S, Lei Z, Yu Z, Liu X, Cui C, Chen X, Shen N, Song C, Qiao J, Xiang X, Li H, Liu D. Development of a Physiologically Based Pharmacokinetic Model for Hydroxychloroquine and Its Application in Dose Optimization in Specific COVID-19 Patients. Front Pharmacol. 2020;11:585021. doi: 10.3389/fphar.2020.585021.

100. Pilla Reddy V, El-Khateeb E, Jo H, Giovino N, Lythgoe E, Sharma S, Tang W, Jamei M, Rastomi-Hodjegan A. Pharmacokinetics under the COVID-19 storm. Br J Clin Pharmacol. 2020. doi: 10.1111/bcp.14668.

101. Puttrevu SK, Arora S, Polak S, Patel NK. Physiologically Based Pharmacokinetic Modeling of Transdermal Selegiline and Its Metabolites for the Evaluation of Disposition Differences between Healthy and Special Populations. Pharmaceutics. 2020;12(10). doi: 10.3390/pharmaceutics12100942.

102. Alsmadi MM. Physiologically-Based Pharmacokinetic Model for Alectinib, Ruxolitinib, and Panobinostat in The Presence of Cancer, Renal Impairment, and Hepatic Impairment. Biopharm Drug Dispos. 2021. doi: 10.1002/bdd.2282.

103. Xu R, Tang H, Chen L, Ge W, Yang J. Developing a physiologically based pharmacokinetic model of apixaban to predict scenarios of drug-drug interactions, renal impairment and pediatric populations. Br J Clin Pharmacol. 2021. doi: 10.1111/bcp.14743.

104. Hu R, McDonough AA, Layton AT. Functional implications of the sex differences in transporter abundance along the rat nephron: modeling and analysis. Am J Physiol Renal Physiol. 2019;317(6):F1462-f74. doi: 10.1152/ajprenal.00352.2019.

105. Geng W, Pang KS. Differences in excretion of hippurate, as a metabolite of benzoate and as an administered species, in the single-pass isolated perfused rat kidney explained. J Pharmacol Exp Ther. 1999;288(2):597-606.

106. Russel FG, Wouterse AC, van Ginneken CA. Physiologically based pharmacokinetic model for the renal clearance of phenolsulfonphthalein and the interaction with probenecid and salicyluric acid in the dog. J Pharmacokinet Biopharm. 1987;15(4):349-68.

107. Komiya I. Urine flow dependence of renal clearance and interrelation of renal reabsorption and physicochemical properties of drugs. Drug Metab Dispos. 1986;14(2):239.

108. Mayer JM, Hall SD, Rowland M. Relationship between lipophilicity and tubular reabsorption for a series of 5‐alkyl‐5‐ethylbarbituric acids in the isolated perfused rat kidney preparation. J Pharm Sci. 1988;77(4):359-64.

109. Follman KE, Dave RA, Morris ME. Effects of renal impairment on transporter-mediated renal reabsorption of drugs and renal drug-drug interactions: A simulation-based study. Biopharm Drug Dispos. 2018;39(4):218-31. doi: 10.1002/bdd.2128.

110. Felmlee MA, Dave RA, Morris ME. Mechanistic models describing active renal reabsorption and secretion: a simulation-based study. AAPS J. 2013;15(1):278-87.

111. Itoh N, Sawada Y, Sugiyama Y, Iga T, Hanano M. Kinetic analysis of rat renal tubular transport based on multiple-indicator dilution method. Am J Physiol. 1986;251(1 Pt 2):F103-14. doi: 10.1152/ajprenal.1986.251.1.F103.

112. Sakolish C, Chen Z, Dalaijamts C, Mitra K, Liu Y, Fulton T, Wade TL, Kelly EJ, Rusyn I, Chiu WA. Predicting tubular reabsorption with a human kidney proximal tubule tissue-on-a-chip and physiologically-based modeling. Toxicol In Vitro. 2020;63:104752. doi: 10.1016/j.tiv.2019.104752.

113. Doki K, Kuga K, Aonuma K, Ieda M, Homma M. Utilizing physiologically based pharmacokinetic modeling to predict theoretically conceivable extreme elevation of serum flecainide concentration in an anuric hemodialysis patient with cirrhosis. Eur J Clin Pharmacol. 2020;76(6):821-31. doi: 10.1007/s00228-020-02861-9.

114. Franchetti Y, Nolin TD. Application of Individualized PBPK Modeling of Rate Data to Evaluate the Effect of Hemodialysis on Nonrenal Clearance Pathways. J Clin Pharmacol. 2021. doi: 10.1002/jcph.1818.

115. Moss R, Thomas SR. Hormonal regulation of salt and water excretion: a mathematical model of whole kidney function and pressure natriuresis. Am J Physiol Renal Physiol. 2014;306(2):F224-48. doi: 10.1152/ajprenal.00089.2013.

116. Czock D, Scholle C, Rasche FM, Schaarschmidt D, Keller F. Pharmacokinetics of valganciclovir and ganciclovir in renal impairment. Clin Pharmacol Ther. 2002;72(2):142-50. doi: 10.1067/mcp.2002.126306.

117. US Food Drug Admin. Clinical Pharmacology and Biopharmaceutics Review; VALCYTE (valganciclovir HCI) Tablets [FDA application no, (NDA)021304]. 2001.

118. Anderson RD, Griffy KG, Jung D, Dorr A, Hulse JD, Smith RB. Ganciclovir absolute bioavailability and steady-state pharmacokinetics after oral administration of two 3000-mg/d dosing regimens in human immunodeficiency virus- and cytomegalovirus-seropositive patients. Clin Ther. 1995;17(3):425-32. doi: 10.1016/0149-2918(95)80107-3.

119. Jung D, Dorr A. Single-dose pharmacokinetics of valganciclovir in HIV- and CMV-seropositive subjects. J Clin Pharmacol. 1999;39(8):800-4. doi: 10.1177/00912709922008452.

120. Sommadossi JP, Bevan R, Ling T, Lee F, Mastre B, Chaplin MD, Nerenberg C, Koretz S, Buhles WC, Jr. Clinical pharmacokinetics of ganciclovir in patients with normal and impaired renal function. Rev Infect Dis. 1988;10 Suppl 3:S507-14. doi: 10.1093/clinids/10.supplement_3.s507.

121. Brown F, Banken L, Saywell K, Arum I. Pharmacokinetics of valganciclovir and ganciclovir following multiple oral dosages of valganciclovir in HIV- and CMV-seropositive volunteers. Clin Pharmacokinet. 1999;37(2):167-76. doi: 10.2165/00003088-199937020-00005.

122. IPCS. Characterization and application of physiologically based pharmacokinetic models in risk assessment. Geneva, Switzerland. h<ttp://www.who.int/ipcs/methods/harmonization/areas/pbpk_models.pdf:> World Health Organization, International Programme on Chemical Safety; 2010.

123. Rodgers T, Rowland M. Physiologically based pharmacokinetic modelling 2: predicting the tissue distribution of acids, very weak bases, neutrals and zwitterions. J Pharm Sci. 2006;95(6):1238-57.

124. Rodgers T, Leahy D, Rowland M. Physiologically based pharmacokinetic modeling 1: predicting the tissue distribution of moderate-to-strong bases. J Pharm Sci. 2005;94(6):1259-76.

125. Yang J, Jamei M, Yeo KR, Tucker GT, Rostami-Hodjegan A. Prediction of intestinal first-pass drug metabolism. Curr Drug Metab. 2007;8(7):676-84. doi: 10.2174/138920007782109733.

126. Scotcher D, Jones C, Posada M, Rostami-Hodjegan A, Galetin A. Key to opening kidney for in vitro-in vivo extrapolation entrance in health and disease: Part I: In vitro systems and physiological data. AAPS J. 2016;18(5):1067-81. doi: 10.1208/s12248-016-9942-x.

127. Peters SA. Variability, Uncertainty, and Sensitivity Analysis. In: Peters SA, editor. Physiologically‐Based Pharmacokinetic (PBPK) Modeling and Simulations2012. p. 161-81.

128. Wenlock MC, Potter T, Barton P, Austin RP. A method for measuring the lipophilicity of compounds in mixtures of 10. J Biomol Screen. 2011;16(3):348-55. doi: 10.1177/1087057110396372.

129. Perrottet N, Beguin A, Meylan P, Pascual M, Manuel O, Buclin T, Biollaz J, Decosterd LA. Determination of aciclovir and ganciclovir in human plasma by liquid chromatography-spectrofluorimetric detection and stability studies in blood samples. J Chromatogr B Analyt Technol Biomed Life Sci. 2007;852(1-2):420-9. doi: 10.1016/j.jchromb.2007.01.045.

130. Certara UK Limited (Simcyp Division). Simcyp Version 19 Help Pages Sheffield, UK2019.

131. Ke A, Barter Z, Rowland-Yeo K, Almond L. Towards a Best Practice Approach in PBPK Modeling: Case Example of Developing a Unified Efavirenz Model Accounting for Induction of CYPs 3A4 and 2B6. CPT Pharmacometrics Syst Pharmacol. 2016;5(7):367-76. doi: 10.1002/psp4.12088.

132. Tsamandouras N, Rostami‐Hodjegan A, Aarons L. Combining the ‘bottom up’and ‘top down’approaches in pharmacokinetic modelling: fitting PBPK models to observed clinical data. Br J Clin Pharmacol. 2015;79(1):48-55.

133. Abduljalil K, Cain T, Humphries H, Rostami-Hodjegan A. Deciding on success criteria for predictability of pharmacokinetic parameters from in vitro studies: an analysis based on in vivo observations. Drug Metab Dispos. 2014;42(9):1478-84. doi: 10.1124/dmd.114.058099.

134. US Food Drug Admin. Drug Labeling-Package Insert: CYTOVENE (Ganciclovir sodium) Injection [FDA application no, (NDA) 019661]. <https://wwwaccessdatafdagov/drugsatfda_docs/label/2018/019661s037lblpdf> [Accessed 26/06/2020]. 2018.
